# ‘The only chance of a normal weight life’: A qualitative analysis of online forum discussions about bariatric surgery

**DOI:** 10.1371/journal.pone.0206066

**Published:** 2018-10-25

**Authors:** Mikaela Willmer, Martin Salzmann-Erikson

**Affiliations:** Department of Health and Caring Sciences, University of Gävle, Gävle, Sweden; Toronto General Hospital Research Institute, CANADA

## Abstract

**Background:**

The only effective weight loss treatment for severe obesity is bariatric surgery, with Roux-en-Y gastric bypass being the most common method. Patients often have unrealistic expectations of surgery and expect a “miracle cure” even though the procedure requires major lifelong lifestyle changes. Most patients access information about the procedure online, and come into contact with others who have had the surgery.

**Objective:**

The objective of this study was to describe shared values, feelings, and thoughts among visitors to a web-based forum for those undergoing bariatric surgery.

**Methods:**

In this cross-sectional observation study using qualitative contents analysis, the material consisted of an online discussion forum thread about bariatric surgery, with 498 posts. These were saved in a document, read and re-read. Through coding of meaningful units of text, themes were established.

**Results:**

Four themes were constructed during data analysis: a) A new life—anticipating dramatic changes of body and mind; b) Negotiating the system and playing the waiting game; c) A means to an end—managing the pre-operative diet; and d) Managing the attitudes of others. Posters described the process of bariatric surgery as a journey, riddled with roadblocks, setbacks and trials, but also with joy and expectations of a new life.

**Conclusion:**

Professionals who encounter this group should be aware of their need for support throughout the process, and investigate the possibility of both pre- and postoperative support groups, either online or face-to-face. The results also show that the posters on the forum had very high, and often unrealistic, expectations on how the surgery would change their lives. It is important for those who encounter this group before surgery to be aware of this tendency and to take measures to ensure that patients undergo the surgery with realistic expectations.

## Introduction

Obesity (commonly defined as a Body Mass Index (BMI) of 30 and above) has increased rapidly in most of the world’s nations during the last few decades. Severe obesity (BMI of 35 and above) has also increased. Whilst the prevalence of all types of obesity is highest in small island nations in Polynesia and Micronesia, where up to 30% of the population is categorized as severely obese, most of the world’s individuals with severe obesity live in high-income English-speaking countries (27%, or 50 million people), followed by 14% or 26 million people in the Middle East and north Africa [1].

Obesity has been shown to be a risk factor for, amongst other things, type 2 diabetes, cardiovascular disease, gallbladder disease, infertility, osteoarthritis and some cancers [2–4]. There are also strong associations between obesity and some forms of mental ill-health, such as depression and anxiety [5,6]. However, this may be at least partly due to the considerable stigmatization of obesity in Western society [7].

Conventional treatment of obesity commonly focuses on decreasing energy intake and/or increasing energy expenditure. However, this approach has been shown to have moderate long-term effects, especially for those with severe obesity [8]. The difficulty for most individuals lies not in losing clinically meaningful amounts of weight, but rather in maintaining the lower body weight long-term [9].

Bariatric surgery (also known as obesity surgery or metabolic surgery) is commonly defined as any surgery performed on the stomach or intestines of a patient with obesity in order to induce weight loss. There are a number of surgical methods and techniques within this concept, the most common of which currently is Roux-en-Y gastric bypass (RYGB) [10]. Bariatric procedures are performed all over the world, with the highest number of surgeries relative to the size of the population being performed in Kuwait, Belgium, Israel, Argentina, France and Sweden [10].

For individuals with a BMI of 35 or above, bariatric surgery has been shown to be the only method that results in major long-term weight loss, with a mean 25% weight loss after 20 years in RYGB patients [11–13]. It also results in improvements, and often remission, of type 2 diabetes, with a systematic review and meta-analysis reporting the remission rate to be 80% 36 months after surgery [14]. In addition to this, bariatric surgery has been shown to result in substantial long-term improvements in cardiovascular risk factors, Non-alcoholic Fatty Liver Disease, and musculoskeletal pain [15].

When it comes to improvements in mental and psychosocial health and Health-Related Quality of Life, studies have generally shown although bariatric surgery results in improvements, these are often transitory and follow the trajectory of weight loss, with gradual changes for the worse occurring as weight loss slows down and/or regain starts. Herpertz et al found this to be the case for anxiety, depression, and self-esteem in their sample [16], and Karlsson et al found similar long-term results for depression, anxiety, general mood and social interaction [17].

Before performing the surgery, most operating units require some degree of weight loss. This is usually achieved with Low Calorie Diets or Very Low Calorie Diets (VLCD), where the patient wholly or partly subsists on meal replacements which provide 600–800 kcal/day [18]. A systematic review and meta-analysis has shown that preoperative weight loss results in shorter operating times and improved weight loss a year after surgery [19]. There are very few studies exploring diet adherence in this group, but logically, not all patients are able to adhere to the diet and thus fail to achieve the desired weight loss.

Regardless of which surgical method is used, bariatric surgery requires the patient to make major and life-long dietary changes, including eating small, nutrient-dense and frequent meals, eating slowly and chewing well, avoiding drinking with food, and avoiding energy-dense and nutrient-poor foods such as sweets, snacks and sugar-sweetened beverages [20,21].

Many previous studies have shown the importance of support groups following bariatric surgery, mainly in terms of improved weight loss [19]. The possible benefits of pre-operative support are less well researched, especially in a context where patients cannot be forced to attend meetings or interventions in order for their surgery to be covered by insurance (as is often the case in the US, but not in countries such as Sweden, where healthcare is publicly funded). However, studies show that patients often have unrealistic expectations of weight loss and the changes they will undergo following the procedure. Kaly et al showed that preoperative surgery candidates would rate a 49% excess weight loss as ‘disappointing’, although this would be considered a successful outcome medically [22]. Shearer showed in his doctoral thesis that surgery candidates expected the procedure and subsequent weight loss to significantly improve their quality of life, and that some, despite being informed to the contrary, believed that the surgery would be a ‘miracle cure’ [23]. As preoperative support groups are rarely, if ever, offered in Europe, it seems likely that patients would turn to the Internet as an important source of information before surgery.

This is supported by the fact that studies have found that the Internet has become the most frequently used source of health-related information [24,25]. There are few specific studies about Internet use specifically amongst the bariatric surgery patient population, but an American study from 2005 showed that almost all of the 127 participants had searched for information about the procedure on the Internet [26]. A study by Koball et al, investigating how patients interacted in Facebook groups following surgery, found that the most common topics were seeking recommendations, commenting on changes since surgery, and lending support to other members [27].

The interest in bariatric surgery remains strong, and the patient population continues to grow. As preoperative support groups are rare, and there is a great need for information and support before surgery. Topics of conversation about bariatric surgery on the Internet may provide caregivers with valuable insights into the need for preoperative support and inform future intervention studies aiming to prepare bariatric patients for surgery and postoperative life in the best way possible. Thus, the aim of this study was to describe shared values, feelings, and thoughts among forum posters visiting a web-based forum for those considering and/or undergoing bariatric surgery.

## Methods

This study was conducted in the naturalistic tradition, applying the tenets of qualitative research in order to meet the aim [28–30]. Sandelowski argued that the naturalistic tradition is a “generic orientation to inquiry” whereas it is not bound to philosophical and/or theoretical connotations [30]. Subsequently, we followed the epistemology of naturalistic inquiry and used the qualitative descriptive method as described by Sandelowski [30]. Our goals of using qualitative description was to gain a comprehensive summary of events in people’s narrative, and their history and events in life via their “testimonio” [31]. At the time when Sandelowski’s paper was published, qualitative data was mostly gathered in interviews or by using other traditional methods. In recent years, it has become more or less mainstream to collect data online [32–34]. The study is a strictly cross-sectional observation study of discussions held in a public online venue.

### Data collection

Google (http://www.google.com) was initially used to locate a suitable forum where discussions of interest were held. The search terms used were the Swedish translations of “Gastric bypass” and “online discussion forum” (“överviktskirurgi” and “diskussionsforum”). The criteria for forum inclusion was: a) publicly available without the need for a username and password to access the information; b) relevant for the purpose of the study; c) an active forum (>10 daily postings, >1.000 postings total, and >100 members); and d) written in a Scandinavian language. Based on the different forums that were identified, both authors agreed that one forum was especially suitable for the study, having been active for several years and thus likely to provide the most in-depth insight for the inquiry. Using a purposeful approach to gather the sample, the authors chose one large discussion thread containing 498 posts, based on its title and content. To facilitate data management, the first author temporarily copied and pasted the thread into a separate Word document to facilitate the forthcoming analysis. After omitting irrelevant information, the raw data comprised of 155 pages of text (S1 Dataset).

### Data analysis

The data were read and re-read as a part of the familiarization process. During the reading, analytic notes, ideas and clues were jotted down in the margin. Both authors took part in the analysis process and made use of our individual areas of expertise. The first author is a well-established researcher in obesity, although with little experience in qualitative analysis; while the second author is skilled in qualitative analysis but lacks deeper knowledge of the condition of obesity and of obesity surgery. Hence, our different areas of expertise were beneficial to an interactive discussion and advanced the analysis. The data were further coded as words and sections of text were highlighted with various colours based on their similarities and differences. For example, the raw data read “I am looking forward to the new me and my new life, can barely wait”. This was coded as “looking forward to the new me“, and “can barely wait”. Both codes were substance in the emergence of the first theme: “A new life—anticipating dramatic changes of body and mind“. The coded text was then grouped into preliminary categories. These were discussed by the two authors and slightly revised. Due to the qualitative nature of the study, a detailed proportion of the distribution of the posts associated with a theme were not counted, but the data were fairly equally distributed in each of the themes. Next, a summarizing and abstracted text was written for each category, and the text from the raw data that was felt to be the most representative was included in the results as excerpts. These excerpts were also discussed during the study, resulting in the replacement of some excerpts by others which were considered even more representative [35].

### Ethical considerations

A strictly archival and cross-sectional study design was adopted, meaning that we did not interact with any human subjects. We discussed the possibility of contacting each poster in order to obtain their consent to use the material. However, after screening the data, it was considered unfeasible, since many of the posters had been inactive for a long time and only used nicknames, which we were not able to reach by private message. Attempts to contact the forum administrator were made, but went unanswered. However, as this study is not considered to be human subject research, no ethical vetting from an ethics board was necessary. Despite this, ethical considerations were of central concern to the authors of the present study. Our intention was, as far as possible, to provide privacy, dignity and integrity for the posters in the discussion forum; and therefore we do not explicitly refer to the name of the forum from which the data was collected. We chose a Scandinavian forum and thus, all excerpts are translated into English and therefore not possible to find via a search engine. All username aliases were removed. In addition, data was only temporarily stored on the researchers’ computers to facilitate the analysis, and no record of the posters was filed [36–38]. During our analysis we deliberately chose excerpts that, whilst being reflective of the thoughts, values and opinions of the posters, were not exoticising nor sensational in nature.

## Results

The findings in this study offer a description of the communication, thoughts, values and feelings of forum posters who visit a web-based discussion forum when considering and/or undergoing bariatric surgery. Four themes were constructed during data analysis: a) A new life—anticipating dramatic changes of body and mind; b) Negotiating the system and playing the waiting game; c) A means to an end—managing the pre-operative diet; and d) Managing the attitudes of others.

### A new life—anticipating dramatic changes of the body and mind

When discussing the effects of undergoing bariatric surgery, posters in the forum often wrote about a “journey” that started with their current, overweight selves, would take them through the healthcare system to surgery and had its end goal in a ‘new life’. This ‘new life’ was often described as one in which they would emerge as different, and better, persons—happier, healthier and no longer doubting their ability to maintain a healthy weight.

*‘I thought I’d jump in here too*, *after many years of struggling with my weight I have now reached “the end” of this struggle and actually thought that maybe a GB is the last way out for me*, *the only chance of a normal weight life?’*

*‘I get nervous every now and then*, *but when I think about continuing the same way as it is now*, *no*, *I can*’*t take that road. I look forward to the new me and my new life*, *I can barely wait.’*

As can be inferred from the posts above, the process of anticipation involved a desire to, one day, have access to *a new life*. Moreover, this strong desire was considered the last option, or ‘the end of the road’, which the members had arrived at after many failed attempts to lose weight. The posts can also be interpreted as reflective of a restrictive perspective of life, where one’s weight status is seen as the one ‘master trait’ that shapes the writers’ entire existence. This restriction caused the posters to adopt a chronocentric way of thinking—that is, a polarization of certain time periods of life, where one specific period is considered vastly superior to all others. For the writers of the forum posts, this superior time period of their lives was expected to come in the future, after the surgery.

The posters also wrote about the ways in which they expected their bodies would change after surgery. These expectations mainly concerned the weight loss itself, and the subsequent relief from aches, pains and co-morbidities caused by their current weight. One poster wrote:

*‘I have a BMI of 39 and long for a lighter existence! Just think how unbelievably good it will feel afterwards!!! Can*’*t wait!’*

In addition to these expected improvements of physical appearance and the expectations of a higher quality of life, many posters believed that the weight loss would bring with it improvements of mental or emotional health problems, such as fatigue, depression and social anxiety.

However, this anticipation of dramatic—and positive—changes was not homogeneous. Some posts also expressed fear of being the exception to the rule—the one person who failed to lose weight even with bariatric surgery, the most drastic weight loss method currently in use. Nearly all the posters wrote about previous failed weight loss attempts, and how they had gradually come to consider bariatric surgery. Several posters felt that being unable to achieve satisfactory weight loss after surgery would be the ultimate failure on their part and would cause considerable mental distress. Fear of failure even made one poster hesitate about undergoing the procedure at all.

*‘Yes, I hope that I can reach a decision for the first step …I think everyone is so brave …I think the fear might be that I’m scared I won’t lose weight except during the liquid diet*, *and then I would feel like a complete failure.’*

*‘Have a fear of failure since you’ve done that so many times*, *but perhaps that’s a common feeling?’*

When these fears were expressed, either the original poster herself or another poster often reassured themselves and each other that this would not happen, the surgery would be successful and a better life would follow. On this and many other occasions, the posters freely gave and received mutual emotional support.

### Negotiating the system and playing the waiting game

Many posts were concerned with navigating the various twists and turns of the healthcare system in order to be cleared for surgery, placed on the waiting list, and finally undergoing the bariatric procedure. Again, the metaphor of ‘journey’ was frequently used to describe attempts to make their way past the various obstacles put in their path. Other members of the discussion forum weighed in with their respective experiences and opinions, forming a kind of alternative knowledge bank which all members used. For example, posters living in different county councils in the country compared BMI limits for surgery and discussed which were the most ‘strict’ and which seemed to be more lenient.

*‘If you are refused or not really depends on in which county you live*, *and what the criteria are for surgery in your county.’*

*‘From what I can tell after some Googling*, *you should have a BMI of 35 or more in order to be granted surgery in X county.:)’*

The first potential roadblock on the way to surgery often came in the shape of primary care physicians. These were described both as gatekeepers, doing their best to stop the posters from reaching the operating table, or as helpers who willingly wrote referrals, answered questions and gave helpful suggestions. For those who were unsuccessful in obtaining a referral from their primary care physician, two options, or ‘system hacks’ were commonly suggested by the others in the forums—writing their own referral (allowed in the Swedish healthcare system) or paying to have the procedure done in a private care facility. The former option was the most common one, and several posters reported having success after getting advice from others what to write and which phrases to use.

*‘My GP seems to be completely anti GP so now I turn to you*. *HOW do I write my own referral???’*

In this phase of negotiating the system, the posters also supported each other, providing advice and posted encouraging comments.

*‘Okay*, *that makes me sad for you …But try sending your own referral*, *but then you have to be very detailed and careful the way I’ve understood it*, *but it’s worth a try? Don’t give up yet if this is what you really want! Stand your ground and try again if you are unhappy with the decision!’*

The other option—paying for a private procedure—was also utilized by a few of the posters. One member stated that she took out an extended mortgage in order to pay for the surgery. Before being cleared for surgery, the posters wrote about going to various group information meetings, seeing dietitians, nurses and surgeons. Whilst some forum members wrote that these had been informative, many seemed to think that they didn’t cover much that they didn’t already know, either from the discussion forum itself or from speaking to others who had already undergone the procedure. The information meeting was often seen as a necessary step to take in order to get closer to surgery, rather than an opportunity to gain information.

*‘As long as you have been given the referral to the information meeting*, *the chances are good that you will get the surgery.’*

The next main concern for most posters was to get the final approval from the surgeon. Many seemed to approach this meeting as an exam or some other major trial that they may pass or fail. Thus, we interpret it as a *critical point of the process*. In order to face this critical point, they prepared themselves by asking for advice and reassurance from other forum members.

*‘Of course now I’m super nervous about the assessment and wondering if anyone feels like telling me what happens and what they ask? (…) I have cycled up and down 30 kilos the last ten years*, *so I’m just over the BMI limit and I have no illnesses (yet) and I’m so scared I’ll get a “no”*, *don’t want to say the wrong things.’*

This critical point in the journey was sometimes also described as the final destination, or at least a longer stop than what they had wished for. One member wrote a long post about how her previous eating disorders prevented her from being approved for surgery, even though she herself felt that she was ‘cured’ and was now desperate for surgery.

*‘ …I’ve been waiting for a yes or a no since March last year*. *I’ve had to go to extra appointments with the dietitian to discuss an old eating disorder (binge eating). (…) I’m getting support from a counsellor in all this but he can’t get me past this roadblock. I weighed in at 142 kilos at the primary health care centre last year and now it’s gone up another 10 kilos.’*

Once the much-desired approval for surgery had been granted, the posters often had to wait for their surgery date. Many expressed impatience and frustration about this, and wanted their ‘new life’ to start right away.

### A means to an end—managing the pre-op very low calorie diet

Almost all the posters wrote about being required to lose some weight before surgery, most commonly with the help of VLCD. This was achieved by swapping all their regular meals for different brands of low-calorie meal replacement shakes, soups and pasta meals. Most of the members had previous experience of losing weight with VLCD, and approached this period with some reluctance and even trepidation. Many of the posts were concerned with different types and brands of meal replacements, how hungry the posters felt when on the diet, and how they were counting the days until their surgery. Aside from hunger, the posters also reported feeling dizzy, tired, angry and emotional whilst on the diet.

*‘I’m hungry most of the time but I try to tell myself that being hungry isn’t dangerous and it’s over soon*.*’*

*‘I’m not looking forward to the powder diet very much*, *I’ve done it so many times. 3 months is the record when did iTrim!’*

The posters had different ways of managing the pre-operative VLCD. Some tried to vary the products they consumed in order to alleviate the boredom of consuming the same things every day, and drank large volumes of water and diet soft drinks. Some also “cheated” on the diet by sometimes eating other kinds of food. However, the main coping mechanism seemed to be to reassure themselves and each other that this would be the last time they had to starve themselves with bad-tasting products in order to lose weight. Thus, it seemed that the knowledge that this was the last obstacle of their journey gave them the required stamina.

*‘It’s tough as heck but in a way it’s also easier since this time you know you’re not doing it “for nothing”. Keep telling myself that it’s the last time I lose weight*. *That makes it a little easier…’*

*‘Tomorrow it’s time to start the liquids. It doesn’t feel like fun at all*, *but I tell myself that it’s the last time and it’s going to go well.’*

These reminders that they were in fact not stuck in yet another cycle of yo-yo dieting, but on a linear journey towards their new lives, seemed to provide much encouragement and was a recurrent topic of conversation. However, ambivalence was also evident in some of the members’ posts—they ‘hope’ or ‘try to convince themselves’ that it was the last time, but seemed scared to whole-heartedly trust the surgery.

### Managing the attitudes of others

To some extent, the forum threads addressed the attitudes of the posters’ social networks outside the internet forum. They posted their thoughts of revealing themselves and what to expect. For example, one poster expressed anxiety about the reactions of those in her immediate surroundings, and turned to the forum in order to obtain advice on how to manage this issue.

*‘I’ve chosen to not go public with this*, *except to family and certain friends. What have you done*, *have you told many people?’*

Another poster replied:

*‘I’ve also chosen not to go public with what I’m about to do*, *except family and colleagues*, *will do it little by little.’*

Others seemed to agree that the best strategy was to adopt a discrete approach when it came to their planned surgery. Also, posters seemed to express ambivalence about the fact that the weight loss they were hoping for would make it impossible to hide the surgery from their surroundings, whilst they also expressed reluctance to make their decision public.

*‘The husband asked yesterday what I will say to people*, *it’s hardly possible to keep it a secret but I’m not going to proclaim it on Facebook*, *either.’*

Although most forum members omitted to give an explicit reason for their reluctance, it was clear based on other members’ posts that they were concerned about negative reactions in the form of patronising comments, or the view that the posters should lose weight the ‘traditional’ way. On some occasions, these fears seemed to be realised:

*‘My husband told his brother who didn’t seem to think it was a good idea. It doesn’t really matter of course*, *but it still feels bad*, *I know he won’t be the only one.’*

The posters used the forum as a safe space to discuss their feelings with others in similar situations, whom they clearly felt closer to than they did to the normal weight individuals around them.

On one occasion, this ‘safe space’ was threatened by a poster who criticised the decision to have surgery, instead proposing that weight loss could, and should, be achieved with energy restriction, increased physical activity and help from a psychologist.

*‘I have followed people in their weight loss struggle one woman weighed 140 kilos*, *she was my height she lost the weight herself today she is normal weight she changed the way she thought about food and learned to eat right (…) The person I know wasn’t given surgery*, *with a dietitian and psychologist*, *physician the person lost the weight and hasn’t gained all the kilos back again. I chose to do it by myself I don’t want surgery have learnt to eat in a better way and better food and move more.’*

This was immediately and aggressively challenged by the other forum posters, as in the following post:

*‘Of course it’s going to make me angry if you come into this thread and (mostly) talk about how you just need to change your way of thinking and eat right to lose weight. Don’t you think all of us here have tried that*, *any number of times? (…) Create your own thread about weight loss and discuss with others who do it your way. Instead of coming in here and potentially making people feel bad about taking or wanting to take this life-altering decision.’*

## Discussion

This study set out with the aim to describe shared values, feelings, and thoughts among forum posters who visit a web-based forum when considering and/or undergoing bariatric surgery. One of the most striking findings to emerge from the analysis is that the posters in many cases shared an idea that undergoing bariatric surgery would cause dramatic changes, both to their bodies and minds, and would in fact give them a whole ‘new life’. These very high, and in most cases unrealistic, expectations have also been found in previous studies, such as the doctoral thesis by Shearer already mentioned [23]. The findings of a study by Homer et al also found that bariatric surgery candidates had extreme and often unrealistic expectations on the results of their surgery, and that these were rooted in the stigma and discrimination that they experienced in their everyday lives [39]. Interestingly, the forum posters in our study wrote relatively little about perceived stigmatisation, although it is inherent in statements such as ‘wanting life as a fatty to end’. Many previous studies have demonstrated the extensive and deep-reaching stigmatisation of people with morbid obesity in all social arenas, including healthcare [40].

The findings of the present study show that the attainment of this ‘new life’ did not come easily. Rather, the posters’ posts on the discussion forum showed an intricate process of negotiation with the healthcare system. There are no national guidelines for bariatric surgery in Sweden, leading to different regions and counties applying their own BMI limits and other policies. Although there are no major differences in these, they clearly still create confusion and feelings of injustice amongst the posters, causing them to turn to each other for information and support. National guidelines for bariatric surgery might not change very much in practice for the patients, but would help to create a sense of equality and certainty which would be reassuring.

Once the first road block on the journey towards bariatric surgery had been negotiated, the posters had to face the next: the preparatory VLCD diet. Again, there were discrepancies between different parts of the country, with some being obliged to follow the diet for longer than others, and some being told they had to lose a certain amount of weight before surgery. Something that all the posters had in common was that they found this period difficult, both physically and emotionally. As has been previously mentioned, there is very little previous research on preoperative dietary adherence amongst bariatric surgery candidates in publicly funded healthcare settings. Reviews have been conducted on the efficacy of different weight loss methods, including low calorie diets, and found it to be satisfactory in the short term, but as has been reported by Gudzune et al, studies rarely report dietary adherence, as opposed to achieved weight loss [41]. A systematic review on preoperative weight loss by Ross et al showed very low attrition rates (approximately 8%), as well as mostly satisfactory preoperative weight loss and liver shrinkage [42]. However, the results of the present study indicate that this weight loss is achieved at the cost of considerable physical and emotional anguish. The current findings add substantially to our understanding of the importance of giving and receiving support from others in the same situation, implying that preoperative support groups—physical or web-based—may be valuable for those who are in the process of preparing for surgery.

Another interesting finding was that several of the posters in the discussion forum had encountered, or feared encountering, negative reactions and comments from friends, colleagues and family. We interpret this as an ambivalence regarding, on the one hand, wanting to lose as much weight as possible, and on the other hand, keeping the bariatric procedure secrete or ‘private’. Previous research, both qualitative and quantitative approaches, have found that social support is of vital importance for successful results of bariatric procedures [19,43].

Previous research on online behaviour before and after bariatric surgery is sparse. The study by Koball et al, on the contents of Facebook support groups, was performed using a quantitative approach, meaning that the most common topics of conversation could be identified by frequency and proportion, but not analysed in any deeper or qualitative manner [27]. A study by Atwood et al used the Social Support Behavior Code to categorise posts on a bariatric support group discussion forum, and found that informational and emotional support posts were the most common categories [44]. Both of these previous studies were conducted using material from the US, meaning that the results are influenced by the specific political and healthcare policy-related circumstances of that country. Nonetheless, it is interesting to see the similarities between their findings and that of the present study, such as the importance of emotional support, which was also a main feature of our results.

Bariatric surgery is commonly viewed as a linear, straight-forward process, prior to and after the surgery [45]. This view of undergoing bariatric surgery is strongly tied to an empiricist view and underpinned by cause-and-effect. However, in light of the results of the present study, we argue that undergoing bariatric surgery should rather be viewed as a journey. This can be compared to recent research in mental illness which, during the last decades, has evolved to view recovery *in* (rather than *from*) mental illness as a journey, rather than a linear process; a journey which includes hope and meaning, but which may also include doubts, fear and backlashes [46,47]. Even though posters may be focused on their own current issues, desires and doubts, the forum made it possible for them to ‘raise their gaze’ and see the bigger perspective by receiving support from others who are, at the very same time, in a different phase of their journeys. Thus, the posters could, through their posts, connect with each other as fellow travellers, even though they were at different points in their respective journeys. Those who had come further could help and support those who were nearer the beginning, and the posters’ differences in perspective, age, health status and occupation all contributed to a diverse and multifaceted discussion.

As shown in the results, the tone in the forum thread was generally very empathetic, kind and supportive. The only instance where the tone deviated from this norm was when someone wrote a post about losing weight in a conventional manner, through energy restriction and physical activity. They were then immediately attacked by other posters and told to leave. This outrage may be connected to the fact that the forum posters had experienced, or feared experiencing, similar criticism in ‘the real world’, from friends, relatives or colleagues, and believed the forum to be a place where they could feel safe in discussing surgery without having to worry about criticism.

### Methodological considerations

Even though this study has provided valuable information regarding the content of online discussion related to bariatric surgery, we acknowledge that it is not without limitations. Since we used a cross-sectional study design, we had limited ability to gain in-depth answers. The possibility to ask follow-up questions would probably have yielded additional insights into the process. However, the strength of analyzing data that were not intended for research in the first place has been argued to hold high levels of credibility [48,49]. Furthermore, as in all qualitative studies, we cannot make general statements outside the studied thread of posts and posters. However, as qualitative studies put focus upon transferability in terms of trustworthiness in the findings, it is up to the reader to get a sense of familiarization of the findings that connects the results to the degree of credibility [50].

Another limitation is that, as the Internet online discussion forum from which we collected our data gives posters the possibility of being anonymous, we have no way of knowing the participants’ ages, occupations, gender, education, or geographical location. These demographical variables would have enabled the reader to assess transferability more accurately. However, there was no ethical way for us to uncover the participants’ identities, and the very fact that they could post anonymously in the forum probably enabled them to be more candid about their thoughts and feelings.

### Conclusion

The aim of the present study was to describe shared values, feelings, and thoughts among forum posters visiting a web-based forum for those considering and/or undergoing bariatric surgery. The results show that the process of making a decision to undergo surgery, and navigating the healthcare system, the preoperative preparations and the reactions of friends and family, can be seen as a journey. As such, it is a process fraught with roadblocks, setbacks and struggles, during which the forum posters provide practical, emotional and social support for each other. Professionals who encounter this group should be aware of their need for support throughout the process, and investigate the possibility of both pre- and postoperative support groups, either online or face-to-face.

The results also show that, in accordance with previous studies, the posters on the forum had very high, and often unrealistic, expectations on how the surgery would change their lives. It is important for those who encounter this group before surgery to be aware of this tendency and to take measures to ensure that patients undergo the surgery with realistic expectations. This might be done in several ways–through oral and written information, simple summaries of research in the area, or through putting patients in contact with others who have undergone the procedure.

## Supporting information

S1 Dataset The de-identified discussion forum thread. (DOCX)
