# Supplementary material for: ‘The only chance of a normal weight life’: A qualitative analysis of online forum discussions about bariatric surgery
Source: PLoS One. 2018 Oct 25;13(10):e0206066. doi: 10.1371/journal.pone.0206066 (PMC6201906; doi:10.1371/journal.pone.0206066)
Supplement: S1 Dataset — (DOCX) [file pone.0206066.s001.docx]

**1**

Tirsdag 26 nov 2015 10:16

Hej alle de vidunderlige mennesker derude! :) 

Jeg har set en tråd om dette emne for 2015, men snart bliver det 2016 og måske er vi flere, der ønsker / håber / burde gøre nogen af ​​disse vægoperationer. 

Jeg selv var i VC, og en henvisning blev sendt til XXXXXX overvægtige enhed. Nu holder jeg alle tommelfingrene og tæerne for at komme derhen på informationsmødet og til sidst få en operation! : D 

Lige nu vejer jeg 117 kg ved 170 cm, hvilket giver et BMI på 40,5. 

Hvordan har du alle andre derude, har du kommet længere end mig? Har du måske allerede haft din driftstid? 

Du er velkommen til at dele din tur og erfaring, jeg vil gerne have nogle pepp, fakta og tanker fra dig.

**2**

Sø 29 nov 2015 20:19 # 1

God aften 

Jeg har været hos lægen og modtaget en henvisning til vægtoperation. Jeg har et BMI på 36, smerter i hofter, ryg, knæ og ankler. Jeg har altid været overvægtig, selv som barn. Der er både diabetes og hjerte-kar-sygdomme i familien. Hvor stor er risikoen for, at jeg vil blive nægtet en operation

**1**

Mandag 30 Nov 2015 13:16 # 2

Hej bananfly! 

Hvis du nægtes eller ej, afhænger meget af hvilket amt du bor, og hvad dit amt har til operationskrav. 

Bedre end det, jeg kan desværre ikke svare, men jeg håber virkelig, det bliver fint! 

Fortæl mig, hvordan det virker :)

**2**

Mandag 30 Nov 2015 16:49 # 3

Jeg bor i XXX , er der 35 der gælder her? XXX har 35, hvis jeg husker rigtigt

**1**

Mandag 30 Nov 2015 18:52 # 4

Hvad jeg kan se for en lille googlande er, at du skal have et BMI på 35 eller derover for at være berettiget til en operation i XXXX :)

**3**

Tue 1 Dec 2015 03:41 # 5

Hej alle :) hopper ind i tråden :) hehe

**1**

Tue 1 Dec 2015 08:13 # 6

Velkommen! :) 

Vil du også gennemgå denne proces?

**2**

Tue 1 Dec 2015 12:54 # 7

Lad os håbe det er 35! 
Jeg er ved at lave en Æske, men det er ikke i XXX , men det skal være "vores" dimensioner endnu? 

Har du modtaget en henvisningsbekræftelse 1 ?

**2**

Tue 1 Dec 2015 12:55 # 8

Hey! :) 
Hvordan går processen til dig? 
Har du været i VC? Hvad slags op vil du gøre?

**3**

Tue 1 Dec 2015 14:03 # 9

Hej! 
Jeg er lige begyndt, måske er det lidt anderledes for mig, når jeg er XXXX :) 
Har lige begyndt at diskutere det med mine læger og lignende, som sagde det, selvfølgelig, bør hjælpe så vente på et møde med en anden læge nu :)

**2**

Tue 1 Dec 2015 14:43 # 10

Okay, hvordan går det efter en så stor operation? Nå hjælper lægen og forstår! 
Er du overvægtig? 

Ved du, om du altid kan møde en læge, efter at vi har sendt henvisningen?

**3**

Tue 1 Dec 2015 14:54 # 11

Jeg er på kanon :) Det var lidt over et år siden operationen var færdig! 

Jeg er 176 til 113 kg! 

Jeg ved ikke rigtig, alt er taget hånd om af mine specialister, så jeg ved ikke, hvordan det virker! : /

**1**

Tue 1 Dec 2015 17:09 # 12

Ja, det skal være XXX målinger, der gælder selv da :) 

Nej ikke endnu :( men jeg håber, det kommer snart! :) 

Hvad angår dit spørgsmål, hvis du kan møde en læge umiddelbart efter en henvisning fra vc, ved jeg ikke rigtig, det kan være anderledes i forskellige amter. Her i XXXX kan du tilsyneladende komme til et informationsmøde i en gruppe og derefter møde en sygeplejerske. 

På noget andet tidspunkt kan du møde en læge.

**4**

Ons 2 dec 2015 22:47 # 13

Hej alle! Jeg vil også gerne komme ind her også! 

Jeg er 164 cm og vejen 108. 

Jeg har været i VC og modtaget en henvisning. Gå til informationssessionen i XXXX den 17. december. Nervøs og håber jeg er heldig nok til at stå op 

Har været overvægtig siden jeg var 5 år gammel. Er i dag XX . 

Håber 2016 bliver vores år! 

knus

**3**

Ons 2 dec 2015 23:47 # 14

At holde tommelfingeren :) er også 24 år gammel! 
Også venter på besked, eller jeg ved operationen vil ske, men ikke når :)

**4**

Torsdag 3 dec 2015 08:55 # 15

Tak! 

Va spændende! Venter føler mig lang og utålmodig, jeg tror haha. Hvornår fandt du ud af, at du fik en operation? Kan du vide det på informationsmødet?

**3**

Torsdag 3 dec 2015 16:24 # 16

Jeg ved det virkelig ikke. 
Jeg har kun møder med mine læger, da transplantationen tager sig af resten. Men har et møde den 21. december, det vil sandsynligvis ske i begyndelsen af ​​næste år!

**1**

Torsdag 3 dec 2015 16:55 # 17

Velkommen! :) 

Åh, hvad spændende og vidunderligt, at du allerede har haft tid til infotraff! : D 

Fortæl mig, hvordan det gik :)

**4**

Torsdag 3 dec 2015 19:55 # 18

Jeg vil helt sikkert skrive så snart jeg har været der 

Følelse uvirkeligt kan være i stand til at få en sådan operation! 

Håber, at andre vil modtage dine opkald til mødet. Alle andre der bor i XXXXX ? 

Ved nogen, hvor længe det er? Det sværeste er nok at ikke vide før du selv rejser dig op eller når det kommer væk. Kan det være fra 3 måneder tilbage til over 1 år? Hvad synes du?

**1**

Torsdag 3 dec 2015 22:01 # 19

Ventetiden er anderledes i forskellige amter, som jeg forstår ... Min far gjorde operationen i XXXX for tre år siden, det tog to måneder fra det første møde til kirurgi. 

Det skal altid være inden for sundhedsgarantien, men hvis alt går langsomt, kan det tage et år at afvente operationen med alle lægebesøg og derefter: /

**4**

Ons 9 dec 2015 08:46 # 20

Åh, hvor hurtigt har din far gjort? Nice det 

Ret for mig ville nok være 6m. For at kunne forberede sig mentalt og lære alt. Hvad synes du? 

Enhver, der har været på besøg / info møde nu?

**5**

Sø 13 dec 2015 05:47 # 21

Håber, at andre vil modtage dine opkald til mødet. Alle andre der bor i XXXXX ? 

Ved nogen, hvor længe det er? Det sværeste er nok at ikke vide før du selv rejser dig op eller når det kommer væk. Kan det være fra 3 måneder tilbage til over 1 år? Hvad synes du?

**5**

Sø 13 dec 2015 05:51 # 22

Hej, ny til familieliv, har ingen idé om, hvad jeg laver :) håber at de er i orden alligevel! Jeg er i øjeblikket interesseret i at gennemføre en GBP og har tid til informationsmøde, 17/12 i XXXXX ! Hold tommelfingeren, at jeg får chancen for at komme op! :)

**1**

Man 14 dec 2015 14:39 # 23

Velkommen her! 

Hvis du kun har modtaget påmindelsen til informationsmødet, er chancerne for, at du får en operation :) 

Jeg holder tommelfingeren for dig!

**5**

Man 14 dec 2015 15:59 # 24

Mange tak, ja jeg håber virkelig, jeg får chancen, jeg vil ikke tøve med i et sekund, som det føles som om det ville være en ny start i livet, hvilket måtte være nødvendigt. 

Mange tak! :)

**5**

Fre 18 dec 2015 17:55 # 25

Var du på mødet i går 4 ?

Hvad syntes du? :)

**4**

Fre 18 dec 2015 22:20 # 26

Heej! Hvor sjovt var vi på samme møde! : D 

Hvor mange vi var! Jeg er overrasket. Men det var okay. Meget meget du vidste allerede, men forstår at de skal gå igennem alt. Jeg føler mig peppered og at den er inden for rækkevidde nu 

Hvad syntes du om det?

**5**

Lør 19 dec 2015 14:10 # 27

Jeg troede tværtimod, at vi ville være flere, der syntes at være mange pårørende, tænkte jeg. Men som du sagde, vidste jeg allerede, men det var stadig rart at høre fra en læge og diætist. Ja, jeg føler mig utilfreds og længe efter det næste besøg, ville ønske de kunne gå hurtigere alligevel;) IVRIG! : D haha.

**6**

Lør 26 dec 2015 21:42 # 28

Hej hver julkendis ???? 

Jeg har ansøgt om at have en gastrisk bypass og har modtaget et første JA, og venter på at få den anden JA af en læge. Hvor passet tror du, jeg kan sætte mig op eller ej? God fortsættelse på dig ????

**7**

Man 28 dec 2015 15:49 # 29

Jeg må bede om, at operationen skyldes, at du ikke kan spise så meget som jeg har fået at vide af mine venner, at jeg vil gøre det, men jeg tror ikke det skal betales, jeg forklarer senere

**4**

Ons 6 jan 2016 16:25 # 30

Hej alle! Hvordan har du det? Har du hørt noget nyt? 

Har du hørt noget nyt 5 ? 

Jeg blev ringet op i går ved forårspausen. Var nødt til at vælge, hvor jeg ønskede at blive opereret. Det var hurtigere end at vente, og der er tilsyneladende ingen køer nu heller, så inden for to måneder bliver det! 

Jeg er nervøs og nu begynder at forberede og kontrollere pulver. På dette har jeg også modtaget et nyt job. Min første faste service! Sååå nervøs. Første dag i måneden 

Nu taler jeg meget. Håber det år okay. Ønsker at dette er en aktiv tråd! Jeg vil forsøge at blive i ofte. 

Kram dig selv!

**4**

Ons 6 jan 2016 16:28 # 31

Hej og velkommen! 

Hvor sjovt du laver så godt. Jeg er usikker på, hvordan det ser ud i andre landsting, men jeg føler her i XXXX at der er en ganske lille chance for at blive afvist så langt. Held og lykke! : D

**4**

Ons 6 jan 2016 16:31 # 32

Hej! Jeg synes, det er noget, du bør beslutte dig selv, hvis det er værd for dig at gennemgå en så stor aftale. Læs om det eller ring vc 

Der er mange andre muligheder forud for vægttab. Håber du fandt noget mere info om gbp. 

Held og lykke!

**5**

Sø 10 jan 2016 18:18 # 33

Hej 4 , lavet en ny konto, de er mig, der er gamle " 5 ": D 

Da jeg blev kaldt uforsigtigt, var det allerede 4/1, den virkelige snap fordi de sagde, at de ville kalde den første af den 11.. Men jo desto bedre er jeg så klar :) Hvilket hospital har du valgt? Jeg valgte XXXX . Håber at blive kaldt af fedmeholdet denne kommende uge.  
Jeg er glutenintolerant, så jeg har ret begrænsede valg, når de kommer til pulverdrikken, men Nutrilett vanille smag virker: D

**4**

Mandag den 11. januar 2016 14:05 # 34

Men vær cool! At være spændende for at se, om vi foregår på samme tid  tror du de kan ringe til dig allerede i denne uge? Eller får du et opkald? 

Ved du, hvad det er for et sundhedscertifikat at tage? 

Jeg valgte XXXX, når jeg bor her og hørte, at det er godt 

Ellers ville jeg have valgt XXXX, det virker godt og sikkert 

Åh ja, du er begrænset, jeg forstår. Find intet andet uden gluten eller noget du kan bestille? 

Jeg ved ikke hvad jeg skal bruge endnu, men jeg forsøgte at prøve Moodifast, som jeg aldrig prøvede. Men ja, nutrilette vanille og chokolade er okay 

Jeg er så nervøs og har haft nogle "kolde fødder" 

Ikke så sjovt. Ofte om aftenen tænker man meget om, hvorvidt det er sandt. Men i dag føles det rigtigt. Meget forvirrende jeg må sige haha. Har du noget sådan? 

Hug!

**8**

Man den 11 januar 2016 15:03 # 35

Ja, men jeg tror, ​​de kan gøre, som jeg forstod det, de kalder dem først fra fedmeholdet et stykke tid, og så sender de et opkald med den tid og forskellige papirer og oplysninger, som f.eks. Heslo-erklæringen. 

Jeg ved faktisk ikke, er for nylig blevet intolerant, så har ikke lært noget endnu, men de løser nok: D som nutrilett vanille, der er heldig;) 

Jasså, hvad tænker du på? 
Jeg har ramt det her nede i lang tid, men nu er jeg så forfærdeligt bombarderet, at jeg vil gøre det, så foretrækker jeg at gøre det i dag, helst i går;) 

Jeg troede, at jeg ville vælge XXXX først, men nu valgte jeg XXXXX i slutningen, måske fordi de har den længste på UP , jeg bor i XXXX , men vokset op i XXXX :)  

knus

**4**

Mandag 11 januar 2016, 22:34 # 36

Hvad spændende! Forhåbentlig kalder de os begge en uge derefter 

Hvad jeg virkelig tror jeg ved ikke, haha. Er meget stoffet jeg er bange for. Eller hvis du får vanskelige komplikationer. Mere sådan. Også spekulerer på, hvordan det drives, når du er gammel xD så godt. Lidt sådan. Lige nu er jeg også sikker og vil starte mit nye liv. Er de dumme før de går i seng? 

Ja ok, jeg forstår! Du bliver syg transport, så hvordan man kommer der er ikke noget problem. Forstå dit valg. De er kyndige! 

Forestil dig, at vi kan betjenes om to måneder! Helt skør. Hvordan viste ventetiden i XXXX ? 

Her i XXXX sagde hun, at der ikke var køer, og efter det næste møde med en kirurg kørte det bare

**Lizzy89**

Tue 12 Jan 2016 10:47 # 37

Ja, jeg venter ivrig efter! Men for hver dag der går, kommer vi til en nærmere dag: D 

Hehe okay ja, stoffet er aldrig sjovt, men samtidig er du i sikre hænder, så der er intet at bekymre sig om :) Og jeg tror ikke, de risikerer at blive gamle efter en sådan op, der ville blive udtalt risici de ville aldrig fungere efter denne metode: D Og komplikationer kan de være, men bare tage det for hvad det er og ser frem til, du klarer dem, hvis de skulle blive noget! Men hold dine tommelfinger ud af problemer!  

Ja, det er, XXXX lr XXXX følte lidt 50/50, men jeg føler, at mange mennesker, der arbejder i XXXX , følte mig som om jeg måske vil være lidt anonym an efterfølgende og ikke nødt til at tale med mange bekendtskaber, når de er nybrugt, så valget faldt på XXXX :) 

De har ingen ventetid, men som de sagde på mødet, er der et garanteret møde med holdet inden for 2 måneder og efter den garanterede Op inden for 2 måneder, hvis alt går som de skulle, så inden for 4 måneder så :)

**9**

Ons 13 jan 2016 09:27 # 38

Hej :) 

Håb betyder, hvis det er okay? 

Jeg er 30 år gammel, vejer 93 kg og er 154 cm. 

Gå til VC på fredag. 

Lidt nervøs, kan jeg sige.

**9**

Ons 13 jan 2016 09:29 # 39

Jeg ser, at der er flere mennesker, der går til XXXX / XXXX for at gøre deres vej op. Jeg bor selv i XXXXX .

**8**

Tue Jan 14, 2016 10:19 # 40

Hej ja jo jo jo jo jo bedre :)  

Okay, spændende, vil gå godt, jeg tror! :)  
Ja, jeg vil gøre mit navn i XXXX, hvis jeg er godkendt, vil sige. Venter på møde med fedmeholdet.

**8**

Tors 14 jan 2016 18:41 # 41

Fik tid til at mødes med holdet i dag, 26/1 er klokken! Længsel! : D

**9**

Fre jan 15, 2016 17:04 # 42

Jeg var på sundhedscentret i dag for at få hjælp med henvisning. Men ingen sendte det. Skal mødes med diætisten, fordi de ikke synes at være min 10 år gamle 

Overvægt i denne sammenhæng. Jeg begynder at give op.

**8**

Fre jan 15, 2016 21:11 # 43

Okay, hvor trist er jeg for din skyld ... Men prøv at sende en selvoptale, men så skal du være meget præcis og detaljeret som jeg forstod, men det var et forsøg værd? 

Giv ikke op hvis dette er det, du virkelig vil have! Stå på og prøv igen, hvis du ikke er tilfreds med beslutningen!  

Hold dine tommelfingre!

**8**

Sø 17 jan 2016 12:27 # 44

Har du hørt andet end 4 ?

**10**

Man 18 Jan 2016 19:48 # 45

Hej der! Håber jeg kan være her :) Jeg har tænkt på denne operation i mange år. Og i dag sendte jeg min egen præsentation til XXXXX her i XXXX . Enhver anden, der sendte en personlig besked?

**11**

Ons 20 jan 2016 19:04 # 46

Hej. 
Hænger på her. Sendt i egen præsentation til 3 steder på mandag, håber på hurtig henvisning bekræftelse! Er så vanvittig træt og kedelig hele tiden, bliver asocial og tænker bare på operation. 
Du føler virkelig, at du er kommet til et punkt, hvor du bare vil stoppe det fede liv. 

Hvor er alle fra?

**8**

Fre 22 jan 2016 11:40 # 47

Hej, forstå din følelse, håber du får et hurtigt svar!  

Jeg er fra XXXX i XXXX .. du? :)

**10**

Mandag 25 Jan 2016 09:15 # 48

Halløj! Sidste mandag sendte jeg min egen præsentation til XXXX og i dag ringede de til! Kom dit onsdag for at veje og måle. Føler allerede som et stort skridt undervejs: D

**8**

Mandag 25 Jan 2016 12:11 # 49

Hej! Hvad sjovt for dig! : D Jeg vil være der i morgen formiddag!

**10**

Mandag 25 Jan 2016 13:24 # 50

Er det da da? ????

**12**

Mandag 25 Jan 2016 13:41 # 51

Hej. Gå ind her, hvis det er okay. Har været i Storbritannien omkring tre uger siden for at få en henvisning og i sidste uge fik jeg svar og nu venter jeg på den første vurdering. længsel

**8**

Mandag 25 Jan 2016 17:14 # 52

Nej, imorgen møder de med fedmeholdet, som skal afgøre, om jeg får kirurgi, de vil veje og måle mig, og jeg vil møde alle i teamet, dvs. læger, sygeplejersker, diætister, psykologer og så videre. Mødet tager ca. 4 timer.  

10 , er den samme form for møde du skal være, er lr informationsmødet i den bane du skal?  

Sjov at vi begynder at få et par st her :)

**10**

Mandag 25 Jan 2016 17:24 # 53

Hvad spændende 8 . Nej, jeg vil bare gå der for at veje og måle. Vil kun tage 5 min. Siden jeg har indsendt min egen anmeldelse, har jeg ikke dokumenteret fra en diætist, så jeg ønskede at komme før de begynder noget :)

**8**

Man 25 jan 2016 21:39 # 54

Okay, det er altid en start! :)

Ja, de vil være spændende, bare håber jeg får en ja til operation! Så nervøs! Men jeg glæder mig meget til i morgen! : D 

Returnerer efter mødet: D

**10**

Man 25 jan 2016 21:55 # 55

Hold dine tommelfingre for dig :) Hvor lang tid har det taget for dig? Hvornår blev din henvisning sendt? Held og lykke til i morgen: D

**13**

Tirsdag 26 Jan 2016 13:34 # 56

Hej 

Jeg kigger på arbejde og kigger rundt, hvis jeg finder et godt forum om lys GBP. 
Der er ikke så meget, men fundet nogle her. 
Jeg ved ikke, om jeg er på det rigtige sted, eller hvis jeg skal ændre tråd til Gastric bypass 2015. Så fortæl mig, om du tror, ​​at 2015 passer mig bedre. 

Ved udgangen af ​​januar 2015 lavede jeg min årlige sundhedstjek på grund af min ADHD-medicin, jeg spiste. Overvægt Jeg vidste jeg var 135kg vist. men det mest bekymrende var, at jeg havde højt blodtryk og havde en forhøjet fedtmasse omkring leveren, der ikke var god og var på randen af ​​diabetes. Det kaldte mange advarselsklokke, at dette ikke vil ende godt, hvis jeg fortsætter som jeg gør.

Så jeg besluttede og startede godt, som jeg altid gør alt hvad angår mad og motion, men efter 2 uger slutter det altid som det har lavet et frit fald. Men dråben var i slutningen af ​​juni sommeren var kommet og alle gik i korte bukser og det begyndte at være dårligt vejr. Min søn var derefter 2 år gammel, og jeg indså, at jeg bare ville være hjemme og skjule   i bløde tøj og ikke gå ud eller tage sig af mig, fordi jeg skam mig for, hvordan jeg kiggede og min fedme.

Så den 15. september modtog jeg min private samtale og lærte mere om mit helbred. Vægtet 133 kg. Han sagde, at når du er gået ned 9 kg, vil jeg kræve   andet sidste hit. Købt pulver kost og kørte ganske strengt. I ugerne spiste jeg æg og pulver weekender hvor de sædvanlige går mad. 3. november havde jeg gået ned på min ni kilo. 24. november   fik   Jeg ringede til mit sidste opkald, hvor de lavede ordren, alle prøver blev taget og masser af oplysninger. Da jeg gik, fik jeg mit opkald til operation. De 16 var her. Husk da jeg stod på skalaen, og det viste, at jeg havde faldet 15 kg. 

nu   det har været omkring 5 uger   sen operation og er gået ned 25 kg + og jeg beklager ikke at gøre det. 
Den store udfordring er nu, hvor jeg er lidt bekymret for, at jeg spiser eller går ind for lidt mad. De siger, at du skal spise 6 gange om dagen og i ca. 20-30 minutter, men jeg kan ikke klare det. Det kan bare ikke gøres. Jeg har morgenmad klokken 8 og sen frokost mellem 11 og 12. Lad mig ikke få en snack mellem disse tider. har testet, men så kontanter var, at jeg ikke skyndte nogen frokost. Så ved jeg ikke, om jeg spiser ernæringsmæssigt, og så får jeg nok kcal.

Har følt svimmel nogle gange, når jeg står op hurtigt   og følte mig lidt sulten til frokosttid. Ellers frisk og frisk. 

Ville være taknemmelig for rådgivning om bare maden, fordi det er den, jeg finder svært at mestre. 

almindelig dag. 

morgenmad: ca. 1 dl tilbage + en macka eller kun 1,5 dl tilbage 
Mellis   intet 
Frokost Den skole tilbyder 
Mellis Ofte, gå tilbage. Let at have på arbejde. 
Aftensmad For det meste kartofler og nogle kødboller eller laks + 1mks creme fraiche 
aften snack   mos med smør og skinke eller æg.

**8**

Tors 28 jan 2016 17:04 # 57

Jeg ved ikke præcis, hvornår min henvisning blev sendt via VC, men fortæl mig, at de var i begyndelsen af ​​november, så var jeg på informationsmødet den 17/12 og derefter mødte holdet, så alting har virkelig gået meget hurtigt ! :) og i dag kaldte de XXXX og jeg fik et JA !!    så i midten af ​​marts driver jeg! Er så glad! Håber, de har det godt for jer!

**8**

Tors 28 jan 2016 17:13 # 58

Hej, sjovt at dele dine oplevelser, altid godt at høre, hvordan andre har det, og hvordan det virker. Desværre kan jeg ikke hjælpe dig med tips lige nu, fordi jeg endnu ikke har afsluttet operationen, men det jeg forstår er, at maden er meget vigtig, og at du spiser så godt som du kun kan. Måske vil du prøve at spise mindre ofte, så du får det rigtige antal mål om dagen, måske kan du øge beløbet så snart som? Og prøv også at spise så godt som muligt i henhold til tallerkenmodellen ...   Jeg har haft tid til midten af ​​marts, hvorefter jeg kan dele mine oplevelser

**10**

Torsdag 28 jan 2016 23:22 # 59

Jeg blev vejet og målt mig i går. Gå til informationsmødet den 25. februar! Så spændende: det og tillykke med det! Hvad er sjovt for dig: D

**8**

Fre 29 jan 2016 10:30 # 60

Tak, det føles rigtig godt: D: D  
Ja, men du er velkommen!   Tillykke med det, nu er du på ægte!

**10**

Man 1 feb 2016 18:21 # 61

Tak! Hvor længe efter informationsmødet fik du en besked?

**14**

Man 1 feb 2016 18:55 # 62

Med risikoen for at trække stemningen lidt ... Jeg har ventet på ja eller nej siden marts sidste år. Jeg har gået meget ud på et diætistmøde for at diskutere en gammel spiseforstyrrelse (hot stool). Bestyrelsen var ikke tilfreds med diætisten ja, men nu står jeg på ventelisten for at tale med en kirurg, før bestyrelsen kan træffe en beslutning. Jeg vil blive kaldt i det tidligste august til dette, og så vil sygesikringen komme ind, hvis jeg får et ja. Jeg får støtte fra kuratoren i alt dette, men han kan ikke tage mig forbi denne bremseklosse. Jeg vejede 142 kg sidste år og nu er det øget 10 kg. Ironisk nok var jeg på VC i dag om en anden sag, og den sidste læge fortalte mig, hvor "Ved du, at du kan få en gastrisk bypass?". Nå ved jeg det ... men dem der siger at operationen er kastet på en, har ikke lavet min tur.

**Martina081014**

Man 1 feb 2016 20:21 # 63

Hej! 
Jeg håber jeg kan deltage i dig i din gruppe.  
Jeg indgav en selvpræsentation til XXXX i begyndelsen af ​​januar og venter nu på at komme i kontakt. 
Jeg har ikke lyst til at få nogen i VG, når jeg bare er "BMI 37", og hvad jeg har læst, skal være svært med 40 kravet. 
Tror du jeg kan sende en henvisning til et hospital uden for XXXX ? Har sagt, at hvis jeg ikke får det, vil jeg gøre det privat. Men de ville have været rart at spare penge for måske fremtidig hudoperation.

**8**

Man 1 feb 2016 20:46 # 64

Informationsmødet var 17/12 - 15, og de kaldte mig allerede 4 / 1-16 gang med fedmeholdet, mødet var 26/1 og de ringede til mig for at fortælle mig, at jeg fik en "ja" den 28/1. Op i midten af ​​marts, har modtaget dato, bare venter på tid. Så alt har virkelig gået meget hurtigt for mig!

**8**

Man 1 feb 2016 20:50 # 65

Hej, hvor trist er det for dig, hvilket amt tilhører du?  
Jeg finder det underligt, at de er så glade for det, hvis det allerede talte i begyndelsen af ​​sidste år?  
Sende et højborg!

**8**

Man 1 feb 2016 20:57 # 66

Hej! Ja, selvfølgelig får du dem, som jeg sagde jo mere jo bedre :)  
Okay, er du blevet godkendt, at du får lov til at deltage i mødet? Er du på vej til iap!  
Okay, BMI 40 er fantastisk, her i XXXX har vi en grænse på 35 og jeg havde "kun" 35,4 i BMI, da jeg blev overført fra Vc.  
Ved, at du kan komme op, selvom du er under BMI-grænsen, men så skal du have sygdomme / lidelser som følge af overvægt. 
Ved ikke, om du kan gå til andre amter desværre.

**15**

Man 1 feb 2016 21:18 # 67

Ja, i mit brev siger de også, at anden hjælp kan tildeles som en 12-ugers pulvermassage ved hjælp af en diætist. De interesserer mig ikke, som jeg har prøvet mange gange med pulver, går jeg ned, men straks efter enden går jeg lidt op: (har været overvægtige i hele livet og forsøgt at gå ned i sen tidlige teenagere. Tror ikke, at kroppen fungerer godt af alle jojobantning måtte det gå igennem.

**14**

Man 1 feb 2016 21:57 # 68

Placeret på XXXX. Sikkert som eddike dette ...

**8**

Tue Feb 2, 2016 09:58 # 69

Okay, men du skal forsøge at stå på egen hånd og sige at du kørte pulverværdien et antal gange uden succes. Er det bare hvorfor du får hjælp via GBP for at slippe af med jojo-effekten, det er at gå ned og blive der.  
Prøv at være stærk og giv ikke op! Hold dine tommelfingre!

**8**

Tue Feb 2, 2016 10:02 # 70

Okay, de virker vanskelige der, tkr, men ... Men måske værd at kalde et par samtaler og finde ud af flere fakta, sundhedsgarantien skal komme hurtigere end det! Det føles okay at vente kun indtil august for at møde en kirurg.

**16**

Tue Feb 2, 2016 16:17 # 71

Hej, jeg hopper ind her. Vil læse tråden senere, når jeg sidder ved computeren. Havde en samtale med en læge i dag, og vi vil sende en henvisning til vægtklinikken om 29.e. 

Jeg er 148cm og vejer nu 79,9. Har faldet 7 kg men har stadig BMI på 36. 

Er fra XXXX og han kunne ikke engang garantere et hit, når halvdelen af ​​alle henvisningerne returneres: / latterligt tænkte han, så han håber at kæmpe for mig. 

Træt af at være overvægtig så håber dette er en begyndelse på en tur til et mere smukt liv.

**14**

Tue Feb 2, 2016 19:11 # 72

Du ved ikke, hvor meget jeg ringede ... har også skrevet til dem og bedt dem om at ringe til min kurator, da jeg afviste fra fortrolighed. Han og jeg har viet meget tid til hvad spisningen skyldes osv. Kuratoren hævder også, at dette er vanvittigt. Har svært ved at se, at alle andre ville være mere mentalt forberedt. Men de har fået noget i min historie og nu sidder jeg fast i nogle limbo ...

**14**

Tue 2 Feb 2016 19:17 # 73

Jeg ville bare tilføje, at jeg forsøgte at få hjælp til XXXX XVP for nogle år siden, men de henviste mig til min egen vc, de kunne hjælpe mig, og jeg ville ikke gå så langt. Jeg har det fint, tænkte jeg og forlod mig af sex. Vc derhjemme, vidste ikke, hvad XXXX betød. På den måde kunne de ikke hjælpe mig. Så denne tur ... Jeg er så træt og har et dødbringende højt ophold. Ja, tyngdeure lige nu. Har arbejdet før. Ønsker du at få den ekstra hjælp til kirurgi, så jeg kan holde vægten nede sent.

**10**

Tue Feb 9, 2016 09:48 # 74

Har læst, at rygning er strengt forbudt 4 uger før op og 4 uger efter. Så jeg har ryget i 4 dage nu. Tænker det bliver lettere, hvis jeg får en operation for at holde op med at ryge lige nu. Men jeg har ikke fundet nogen info om snus? Vil helt sikkert svare 25 på gruppemødet, men tænkte, hvis du ved det?

**17**

Sø 14 feb 2016 12:25 # 75

Hej alle! 
Jeg håber at komme ind her. Jeg er en XX -årig pige fra XXXX, som vil fungere i 2016. 

Min tur startede ved at sende en henvisning fra vc til XXXX (hvor de gør GPX her i XXXX ) den 30/9 2015. Jeg var på gruppemødet den 19/11 og mødte derefter kirurgen, som godkendte mig den 10/12. Ved kirurgen havde jeg et vægtmål, jeg var nødt til at nå, før jeg kunne få tid til operation.Skal falde 7% af min vægt, som var 10 kg (dog øgedes 1,3 kg i weekenden, hvilket betød, at jeg måtte gå ned 11,3 kg i stedet). Den 14/12 2015 startede jeg min kost, siden da er jeg gået ned 16,8 kg. Har således krydset grænsen. 

23/2 Jeg vil være på tilmelding. 1/3 jeg går ind og 2/3 sker min operation! 

Jeg startede på 144,3 kg til min 179 cm (ca. BMI 45) og vejer nu 127,5 kg (BMI 39,8). Jeg har en lang vej ned til 70 kg, men operationen vil helt sikkert hjælpe mig med at nå mit mål. 

Du er velkommen til at følge alles tur og vil hjælpe og støtte så godt jeg kan. 

Jeg har en blog, hvor jeg skriver om min tur, alle interesserede i at tjekke det er adressen XXXXXXX :) 

Held og lykke til alle!

**8**

Ons 17 feb 2016 12:38 # 76

6 uger før i henhold til mit papir :) Men de er meget gode til at afslutte allerede. Faktisk ved jeg ikke hvordan man snus Jeg ved ikke, hvordan de er, har ikke registreret så meget om dem, når jeg heller ikke ryger, men at benytte lejligheden til at afslutte alt er bedst, selvom de er vanskelige. Held og lykke!

**8**

Ons 17 feb 2016 12:43 # 77

Hej! Hvilken stor og effektiv tur har du haft! Og dejligt at du allerede har passeret vægtmål, jeg har ca. 1,4 kg tilbage til målvægten, og jeg kører 3/3, det er dagen efter dig! : Hvordan har du det med pulverføde?

Jeg startede min rejse på 111 kg til min 170 cm, nu ned 103 kg, men gå ned til 101,6 før op kan finde sted, men jeg bør håndtere dem!

Vil helt sikkert tjekke din blog! :)

Mange tak!

**18**

Ons 17 feb 2016 13:22 # 78

Hej alle sammen 

Håber at indsætte her. 

Gik på vc og venstre prøver i går og vil møde lægen i XXXX på onsdag. 

Skulle faktisk have betjent mig i begyndelsen af ​​2014 (af sundhedsmæssige årsager, men også for at blive gravid), men efter at jeg faldt 10%, kaldte og stoppede mig, begyndte jeg at føle mig dårlig om morgenen. I dag er jeg og mine samlevende forældre en dejlig lille dreng 

Men nu kører processen igen, og hvis jeg får en ok for operation, vil jeg helt sikkert gøre det. Forsøger at læse alt, jeg kommer over, og har også bestilt Odin's opskriftbog første gang efter operationen.

**8**

Vores 17 Feb 2016 20:08 # 79

Hej, du er så velkommen så :)

Hvor dejligt for dig at processen kører igen, håber det bliver effektiv og kort :)

Og tillykke med din lille fyr, hvad en belønning for dit 10% vægttab! 
Hvilken vidunderlig følelse det måtte have været, da du plussed efter at have troet, at fedme gjorde det umuligt!

**17**

Lør Feb 20, 2016 19:51 # 80

Ja, men virkelig super flot! Især som det ser ud til at have været lidt i denne uge. Du vil være sikker på, at du vil have en og en halv uge på dig. Der er ikke noget problem! :) Hvad sjov du arbejder den følgende dag, må jeg spørge i hvilken del af landet? :) Vi har ikke noget pulver krav, så jeg har lige drevet til at tælle kalorier. Jeg kan godt lide mad for meget for at give det op: P Hvordan har du det? 

Men så er du snart under trecifret! Hvilken vidunderlig en, du er vild, indtil jeg er der.

**8**

Sø 21 feb 2016 17:42 # 81

Jeg kører XXXX , XXXX :)  
Ja, men Gud er dejligt for dig, pulveret er ikke længere! Har foretrukket at tælle kalorier, men de arbejder har spist kosten halvvejs i dag, tage en dag ad gangen  

Ja, det er nær nu, længes indtil jeg er tocifrede    og det vil være rart at være i stand til at forblive på tocifret basis ved hjælp af operationen!

**17**

Sø 21 feb 2016 22:07 # 82

Nej, men hvad sjovt med en til XXXX ! : D Kan du spørge, hvor du er fra? Jeg er fra XXXX , men lige nu bor jeg i XXXX . Hvis det hopper mere fra området, ville det være sjovt at køre lidt gbp hit engang :) 

Held og lykke på onsdag, synes ikke at være nogen store problemer, der skal godkendes her, så det er nok godt :) Nå, jeg kommer til tirsdag for tilmelding, vil være en hel dag. Jeg ønsker også at tabe sig primært for at blive gravid, ikke fordi jeg selv har nogen fyr lige nu, men det er vigtigt at starte i tide. Har pcos som gør det sværere så :) 

Tillykke med lillkillen forresten! :)

**15**

Tirsdag 23 februar, 2016 09:45 # 83

hej piger!  
Håber, at de går godt for alle, jeg tænker på at sende en henvisning til noget mere end mit eget, da jeg har forstået, at de er så hårde med 40 grænsen her. Er de nogen, der ved, hvilket amt der er mere "venligst"? Jeg har bmi 37.

**17**

Tirsdag 23 februar 2016 20:34 # 84

Aha okay, XXXX (eller hvad hedder: P) eller? Jeg har hørt masser af gode ting om :) 
Ja jeg forstår det, jeg har aldrig været i stand til at gå på pudret kost for mere end et par dage, hvor jeg virkelig hader smagen af alle disse poser. Ud over Modifasts puddinger kan de ikke spises hele tiden. Held og lykke med resten af ​​strømmen :) 

Ja, jeg føler det samme, tror det til sidst, at du kan gå ned der og derefter faktisk forblive der. 

Jeg var på tilmelding i dag og derefter spurgt om jeg kunne åbnes tidligere, så nu er jeg på mandag 29/2 i stedet. Nervøs!

**8**

Vores 24 Feb 2016 16:39 # 85

Ja, men bare hvor skal jeg OPA'er Nah, jeg ved, det er overstået, men i dette tilfælde et must, så jeg har taget mig i kraven, men det er svært, vil jeg ikke lægge skjul på,! men nu er det kun 1 uge tilbage heldigvis! Nej, men vær cool! Tillykke, jo hurtigere jo bedre! Avis Jeg vil være men forstå, du er nervøs, men de vil gå så godt! Og GBP-møde ville være sjovt, selvom jeg ikke er fra dit område 
 

    

**18**

Vores 24 Feb 2016 16:40 # 86

Jeg bor i en by uden for XXXX , besat 10 år siden. 

Fik i dag at vide, at jeg ikke behøver at deltage i gruppen info, jeg vil nu gå ned 4 kg og så vil jeg ringe og bog. Må ikke være bekymret for det, fordi jeg ved, at en uge på kun suppe giver det resultatet. 

Nu blev alt pludselig så rigtigt !!

**19**

Tirsdag 25 februar 2016 10:26 # 87

Hej alt fint! 

Også troede at hoppe i her, efter mange års kamp med vægt, har jeg nu kommet til "ende" af denne kamp, og faktisk troede, at måske en GB sidste udvej for mig, den eneste chance for et normalt liv er vigtigt? 

Har nu fået lidt tid til VC 21. marts så nu er virkelig nervøs for, at jeg faktisk tog et stort skridt ved at bestille nogle gang vedrørende denne OP, 
har nok XXXX værste tillid (det føles alligevel), og er nu vokset træt af føler mig som et offer, ønsker ikke at skamme sig over mig selv og vil føle mig godt tilpas blandt mennesker. Og i XXXX sidste år fik jeg en søn, og jeg vil have et aktivt liv med ham, så min største motivation er bare min elskede lille søn. 

Har foretaget en enkelt operation i mit liv, en kejsersnit. Men der blev jeg ikke stukket, og dette var ædru skræmt mig meget, selvom jeg ikke var bange eller nervøs for det skære, der kom pludselig. Så skal du få en OP, hvor du ikke engang er vågen :) 

klem dig!

**12**

Tue Feb 25, 2016 11:34 # 88

Hej 

I går fik jeg endelig opkaldet til gruppemødet. Nu føles det virkelig som om det er rigtigt. Bare håber jeg er godkendt af kirurgen. Ved ikke hvad jeg ville gøre, hvis jeg ikke fik det. 

Så nu skal jeg til min VC og læse mange prøver og hvis det er mindre end en måned er det tid til gruppemødet ????

**18**

Mandag 29 Feb 2016 12:55 # 89

Hej og velkommen 19 Det er dejligt for dig at tage dette skridt, jeg ved selv, at jeg tænkte i lang tid at tænke på, at jeg måske ikke har kæmpet nok mig selv til at tabe sig. Min partner og jeg så også operationen som en mulighed for at blive forældre. Nu fik vi det uden kirurgi, men ligesom du skriver, vil jeg i stedet fokusere på at være en aktiv mor og være aktiv partner. Jeg vil også føle mig bedre og have mulighed for at forhindre fremtidige problemer, som overvægt betyder. Nu ser vi frem og håber det går godt. Held og lykke på VC !! Kram, klem 


**18**

Mandag 29 Feb 2016 12:58 # 90

Hold tommelfingeren for dig, hvis dine prøver ser godt ud, bør der ikke være nogen hindringer for en operation. 

Hvor skal du arbejde? (Du har sikkert skrevet det, men min computer er så) 

Held og lykke!

**18**

Mandag 29 Feb 2016 13:33 # 91

Hej alle i tråden. 

Hvordan har du det? Hvor i processen er du? 

Jeg selv startede min dag med supper. En chokolade til morgenmad og en grøntsagssuppe til frokost. Skal gå ned 4 kg og derefter ringe ind for at stå op i OPkö. Da jeg gjorde det, sagde min læge, at det er valgfrit, hvordan jeg fortsætter med at spise indtil operationen. Hun anbefalede at erstatte 1 til 2 måltider om dagen til suppe, selvfølgelig vil jeg ikke tabe igen. Jeg troede, før jeg mødtes med lægen, at jeg ville få kravet om at gå ned 10% og derefter fortsætte suppe, indtil det er tid til operation, men XXXX har stillet sine krav. Det føles rart at kunne variere lidt og fortsætte med at spise mad med lidt mere tyggemodstand  

**12**

Ons 2 mar 2016 08:46 # 92

Tak :) Jeg kører i XXXX ( XXXX ) og her er ventetiden omkring 1 år. Men efter at have tænkt over dette i flere år, føles det stadig så tæt

**18**

Ons 2 marts 2016 12:25 # 93

Hej XXXX . 

Hvordan er det gået? Hvordan går det efter operationen?

**18**

Tue 15. marts 2016 12:06 # 94

Hvordan er alle i tråden?

**12**

Fre 18 mar 2016 10:14 # 95

Hej alle !! Nu har jeg gået til gruppens information og mødt med kirurgen. Der var virkelig en masse oplysninger at tage ind og holde kysten nu for at tage i alt. Det føltes ret skræmmende, da du hørte om alle bivirkninger og noget, der kunne gå galt. Og kirurgen sagde, at det ikke er sikkert, at alle vil føle sig godt efter operationen, og risikoen er altid, men jeg føler så stærkt, at det er det rigtige for mig. 

Jeg sagde, at hag var mest interesseret i at få en duodenal switch, men ville også acceptere at lave en regelmæssig Gbp. 

Nu venter jeg på det næste skridt, som er at møde dietist og sen psykolog. 

Også fundet at ventetiden er omkring 7-8 måneder, så forhåbentlig arbejder jeg inden årets udgang. 

18 Hvor langt har du ankommet nu? 

Hug

**13**

Fre 18 mar 2016 13:10 # 96

Jeg vejer 2 gange om måneden. 
Har udslettet bølgen i mange år, da det bare forårsagede mig angst. 
Så jeg går til mit sundhedscenter og vejer mig hver anden uge. Føler sig rigtig 
at gøre det på samme bølge samme dag og tid. Så har jeg skrevet i min 
telefon min vægt tur. ser næsten altid frem til at veje mig nu.

**20**

Lør 19 Mar 2016 22:53 # 97

Hej alle! 

Vil du gerne læse om dine rejser. Jeg hopper også herinde! 

Jeg var en af disse GBP modstandere før ... Troede det var "den nemme måde" at alle slackers tog: / Jeg ved nu, hvor forkert det var af mig at tænke så! 

Efter mange års sultende og hård træning har jeg givet op. Jeg kan ikke tabe :( Efter nogle lægeundersøgelser viste, at jeg har stor hormorrubbningar og hypothyroidisme, som forklarede min manglende evne til at tabe sig. Sååå værdiløs !!!! 

Så efter at være blevet overtalt af min terapeut bør jeg OPA'er nu Lige nu går jeg på flyderen kost (Modifast) for at nå målet vægt 116 kg. Men efter 2,5 ugers pulver, har jeg kun tabt 1,2 kg, så selv der synes at arbejde. 

Men det er muligt, at det vil jeg blive drevet på 29/3! Hold dine tommelfingre!

**18**

Ons 30 mar 2016 10:07 # 98

Hej alle sammen. 

Ville virkelig mødet med en diætist, sjukgymnas, læger og narkoselæger på torsdag, men kunne ikke tage prøver før, fordi det var en nødsituation transport til sønnen med ambulance. Han blev åndenløs og blev bevidstløs på børnehaven. Han føler sig meget bedre, men han bliver nødt til at lave et par flere prøver, derfor slog jeg mig et stykke tid. Jeg talte med kirurgi planner, som informerede mig, at hvis jeg ringer til dig i slutningen af April, så jeg har tid endnu til at blive betjent inden sommeren, når XXXX pause alle operationer i midten af juni. Hun var lidt skuffet over mig, men jeg forklarede, at sønnen stadig går før alt andet, og at jeg vælger at udskyde mine tider. 

Så nu håber jeg, at Lillemanens prøver ikke viser noget, og jeg starter igen. Da jeg var parat til at kunne operere først i efteråret, kan jeg ærligt nok ikke skrive noget om det efter sommeren, men det afhænger af, hvordan tingene sker. 

Hvordan har du det med andre?

**16**

Ons 13 april 2016 18:01 # 99

Jeg fik mit svar en henvisning i dag, men desværre var jeg involveret i traumer før vi sendt det, men jeg ville så gerne have dette, især nu, at have noget at se frem til så ikke indstille tidspunktet for indbringelsen trods hændelsen. Jeg vil ikke gå til vægtklinikken før jeg "føler mig godt" i 6-12 måneder før ... suger. Havde så set frem til dette som en positiv ting i mit liv lige nu, men er ustabil, kan blive enige med, men ikke rigtig. Dette havde givet mig glæde. At en person kan tage så meget fra en.    

**21**

Ons 20 april 2016 16:54 # 100

Hej til dig! Gå ind i denne tråd, jeg er okay! 

Sendt i egen præsentation i midten af ​​marts o har nu modtaget et opkald til gruppeinfo på XXXX den 9. maj! Enhver, der kender til operationer "ligger nede" om sommeren eller hvis de løber som normalt? Hvordan har du i tråden?

**22**

Tors 21 april 2016 14:54 # 101

Jeg troede håber herinde. Jeg begynder at være nervøs for at blive nægtet OP ... Hvilke flere mennesker tilhører Lycksele? 

16-03-15 Tid på sundhedscentret 
16-04-05 Skriftlig bekræftelse fra XXXX 
16-04-14 Gruppemøde i XXXX 
16-04-19 Indkaldelse til kirurg 
16-05-17 Møde med kirurgen i XXXX

**21**

Tors 21 apr 2016 22:12 # 102

Ja, der er det hurtige bud! Fantastisk, at det ikke ser ud til at være så lang ventetid! Hvad er det der får dig til at tro, at du ikke får en operation? Skal du være et godt tegn, når du gik så lang tid i processen?

**22**

Fre 22 apr 2016 07:13 # 103

Det er nok bare min negativitet, der gør mig rolig, selvværd er noget, du er nødt til at arbejde på samt :) 

På gruppemødet sagde de, at de opererer to dage om ugen, og tid med 3 / dag 
i XXXX betjene dem, indtil midsommer Så bryder alt og genstarter i august

**18**

Mandag 25 Apr 2016 10:49 # 104

Jeg tilhører XXXX Spændende for dig, nu har gruppeslaget været, hvordan har det det? 

**22**

Tirsdag den 10. maj 2016, 18:14 # 105

Nu er det 1 uge tilbage til mit møde med kirurgen ..... så .. ca. 1 uge ved jeg, om jeg får et OK-stempel i ryggen eller ej.

**18**

Ons 11. maj 2016 21:41 # 106

Spændende. Jeg holder tommelfingeren for dig. 

Jeg vil møde en sygeplejerske, en læge, anæstetiker, diætist og fysioterapeut næste uge

**23**

Mandag den 16. maj 2016 18:49 # 107

Hej!

Min læge synes at være fuldstændig anti-GP, så jeg vender mig til dig nu. Hvordan skriver jeg en personlig kommentar ???

**22**

Mandag den 23. maj 2016 00:10 # 108 **+1**

wihooooo ... godkendt til OP, vil gå ned 5kg og derefter opkald planlæggeren og bog tid

**23**

Ons 25. maj 2016, kl . 19:10 # 109

Sendt en privat præsentation til XXXX og modtog et opkald den følgende dag. Modtaget et opkald til informationsmøde i midten af ​​juli ????

**24**

Tirsdag 7 juni 2016 11:53 # 110

Jeg vil også deltage i mødet i XXXX . Sikkert vil de være spændende? :)

**23**

Tors 30 Jun 2016 08:51 # 111

Ja helt sikkert !! Lidt nervøs, jeg er alt.

**25**

Tirsdag 6 Sep 2016 12:03 # 112

Hej, 

hvordan har det været for jer alle? 

Jeg er selv blevet reserveret til operation i november. 

Jeg vil gå ned 5,5 kg indtil da og flyde 2 uger før. 

Jeg vil fungere i XXXX . 

Vil gerne have kontakt til en person, der er blevet betjent eller på arbejde, så vi kan lave lidt mad med hinanden og er taknemmelige for alle råd og ideer om ting, som du kan tænke på både før og efter operationen.

**26**

Tirs 29 sep 2016 09:22 # 113

Hej! 

Jeg har tid til operation i slutningen af ​​oktober og begynder med pulver næste uge. Føler spændende og lidt skræmmende selvfølgelig! Har et BMI på 39 og længes efter et lettere liv! Enhver anden skal betjenes snart?

**27**

Sø 2 okt 2016 20:13 # 114

Hej! 

Jeg har tid i begyndelsen af ​​november. Hvor skal du blive opereret?

**27**

Sø 2 okt 2016 23:01 # 115

Jeg vil fungere i XXXX . På XXXX ! Du?

**26**

Man 3 okt 2016 08:18 # 116

XXXX , privat klinik. Jeg har et BMI på 33, så jeg skal betale mig selv. 

Har det taget lang tid for dig?

**26**

Man 3 okt 2016 12:40 # 117

Nej. Gik på gruppepause i juni, så jeg tror det er gået hurtigt! Jeg gør også min private. Kunne have fået det betalt, men det følte sig så meget glattere på denne måde. Jeg føler mig meget sikker og taget hånd om på XXXX , det er meget værd. Og min operatør er en af ​​storhederne i området i Sverige, så det føles godt.

**26**

Man 3 okt 2016 12:41 # 118

XXXX skal være. ????

**27**

Man 3 okt 2016 12:48 # 119

Spændende! Du kan love at fortælle mig, hvordan det går, så jeg ved hvad jeg har for mig.

**26**

Søn 9 okt 2016 19:01 # 120

Hej alle! 

Nu har jeg pulveriseret siden mandag. Er gået ned fem kilo siden da. Det er hårdt som helvede, men på nogle måder endnu lettere, fordi nu ved vi jo, der ikke gør det "unødvendigt". Fortæl mig, at dette er sidste gang jeg taber. Så bliver det lidt lettere ... operationsdagen nærmer sig. To og en halv uge tilbage nu! Føles meget nervøs, men jeg er klar! 

Hvordan har du det med andre?

**27**

Søn 9 okt 2016 21:05 # 121

Hej hej! Jeg behøver ikke at køre pulver eller flow, hvis jeg ikke vil. Jeg troede stadig, at det ville tage en uge før operationen at lægge et godt fundament og at tørre leveren for sikkerheden. Det gør ikke ondt alligevel! Jeg starter om et par uger. Tips om noget godt? 

Hvordan har du til hensigt at gøre med mad efter operationen. Køber du hjemme og planlægning?

**25**

Tue 11 Okt 2016 09:29 # 122

26 

De er virkelig godt færdige, at du allerede er gået sååå passere. 

Jeg tænkte på at flytte lidt tidligere, men så begyndte jeg to uger før. 

Ja, vær bange for ikke at give slip på, hvad jeg skal stå op for. 

Men jeg har kun 2 kg bevaret, og operationen er ikke indtil 1,5 måneder 

nu engang har min mand og min mor læste mit papir om gastrisk bypass, så enhver hjælp på støtte og frem for alt vide lidt om, hvad det betyder ??? ?

**28**

Søn 16 okt 2016 04:31 # 123

Hej! Jeg er ked af at komme ind her og forstyrre lidt, men jeg har meget om og men besluttede at lave en mavearm og undre om nogen kan tale om gode blogs eller instagram konti at følge? Må ikke rigtig lykkes med at finde dig selv; De blogs jeg fandt er ikke blevet opdateret om et par år, og hos Instagram søger jeg for det meste svenske konti

**25**

Søn 16 okt 2016 09:56 # 124

28 : 

Desværre har du ingen idé. 

Kan du spørge, hvordan man skal bestemme på ærmet?

**26**

Tirsdag 18 okt 2016 03:17 # 125

Har nu flyttet i to uger og faldet 8 kg. Havde en "pause" lørdag, da det var 40 års fødselsdag, men kører nu igen. Ti dage til kirurgi! De gav mig ingen specifikke kilo at gå ned, men jeg har forstået, at det bliver lettere for operatøren, hvis leveren er blødere. Har også studeret studier, der viser, at vægttab på længere sigt har tendens til at være mere vellykket, hvis du går ned, før du åbner op. Jeg håber at komme ned til 105 kg før åbningen. Vejer 111,7 nu til min 176 cm. Kan du spørge, hvad andre mennesker vejer / måler? 

I hvert tilfælde bemærker jeg en klar forskel allerede på dette lille vægttab. Har ingen smerter i dine ankelled og knæ længere og berør mig lettere. Tænk så slemt, det vil blive følt da !!! Længsel! Min målvægt er et sted mellem 70-80 kg. Men vi vil se, når jeg er der, hvordan det føles.

**25**

Tirsdag 18 okt 2016 10:08 # 126

26 : 

Hvordan føler du strømmen er gået, føler du hele tiden sulten, eller du kommer ind i det efter et par dage. 

Jeg havde planlagt at flyde med Modifast, så kan du spise pasta, supper og selvfølgelig rystelser. Så det vil fungere bedst for mig. Chokoladepudding Maj Ja, Glem ikke ???? Det er verdensklasse. 

Ja, det begynder at komme tæt på dig, spændende. 

Jeg er overrasket over, hvor hurtigt dette er gået fra tid til sundhed center til driftstid, jeg troede det ville tage meget længere tid, 

så er min tid gået ud

**26**

Tirsdag 18 okt 2016 14:44 # 127

Hey 25 ! 

Ja, det er spændende! Og skræmmende. Jeg forsøger at forestille mig selv, når jeg er bange for, at dette er det største, jeg kan gøre for mig selv. Denne tur er til mig for mig at føle mig godt. Det giver positive effekter for alle i min nærhed. 

Jeg har flyttet ved hjælp af itrims produkter (jeg gik deres program for nogle år siden og ved, at jeg kan lide dem). Chokolade Chop, Broccoli Soup og en færdig shake, der smager blåbær / vanille Jeg kan godt lide meget jeg har taget. Jeg har lige kørt på vlcd og ingen produkter snyder. Jeg er mest sulten hele tiden, men jeg forsøger at forestille mig, at det ikke er farligt at være sulten, og det er forbi. Drikker også meget vand, det hjælper lidt! Hvornår har du oppetid?

**25**

Tirsdag 18 okt 2016 20:58 # 128

Ja selvfølgelig gør du det selv, og dit helbred er vigtigt for hele din familie. 

Du vil være i stand til at spille mere med børnene, for at komme i gang med træning for at vise dem et sundere liv. Og desuden vil du føle dig godt og nyde dig selv. 

Og de vil gøre alle glade. 

Vi har prøvet alt før, så det er den sidste løsning, og med dette værktøj vil vi begge være sikre på at lykkes ???? 

Jeg bliver nervøs nu og da, men når ja, tror jeg, om jeg vil fortsætte som nu, nu kan jeg ikke tage. 

Jeg ser frem til mit nye mig og mit nye liv, kan næppe vente. 

Åh, du er så tæt på nu. 

Jeg vil ikke stå op til den 24. november. 

Tager de flere prøver sent, inden de er op, eller de er bare de prøver, som sundhedscentret tog i starten af ​​denne rejse?

**26**

Tirsdag 18 okt 2016 22:29 # 129

Jeg gør min operation privat (har ikke "nok" høj BMI), så jeg ved ikke rigtig, hvordan det virker for dig. Bestemt lidt anderledes, uanset hvor du bor. På mig tog de alle prøver om en måned før åbningen. Leverstatus, blodstatus, triglycerider, jernaflejringer, blodkoagulation og bastest traume, de ved. Eventuelt yderligere ....

**25**

Ons 19 okt 2016 22:21 # 130

De samme prøver du tog tog jeg også før jeg kom til infomotive. 

Faktisk talte til XXXX i dag, og der var ikke flere tests før. 

Mine prøver viste derefter lidt højt sukker, kan på lang sigt indikere, at de kan udvikle sig til diabetes. 

Samt høj leverværdier, der viste sig at skyldes galdesten. 

Så jeg bekymre lidt for galdestensanfald efter OP, 

men de sker som de sker 

, det kan vel ikke føle værre end fødslen ???? 

Hvor længe vil du være syg da, 

jeg spekulerer på, hvor slemt det er bagefter, 

jeg hørte, at de fleste mennesker har den mest smerte i akslen / tilbage på grund af gassen. 

Ja e-mail efter barselsorlov, så jeg vil gå hjem under alle omstændigheder, men min mand vil være fri fra arbejde i tre dage til at lindre mig. 

Jeg håber det er nok, at jeg kan tage Ann mig alle arbejdspladser siden de tre børn stadig: indsamling, aflevere i skolen, børnehaven og tage sig af den lille 

'll se, hvordan du føler efter og baseret på ???? 

Fantastisk at have nogen gøre det før, så det bliver lidt mere forberedelse.

**26**

Fre 21 okt 2016 22:59 # 131

Ja, det er spændende! Har nu pulveriseret i næsten tre uger og faldt 10,5 kg. Virkelig træt af det nu, men snart i mål! 

Enhver, der har tips om proteinpulver, der er godt? Jeg troede du kunne få det bagefter i smoothies / pandekager mv.

**25**

Lør 22 okt 2016 08:07 # 132

De e de gigantiske kudos til dig. 

Jeg målrette i to uger, er i stand nok ikke mere, jeg tror 

vi kommer til at XXXX i to uger, og jeg begynder min flydende efter en uge der. 

Så den første uge, så får godt spise nogle længe ventede churros derefter strengt flyde. 

Fik en anbefaling fra vores ernæringseksperter på XXXX på et proteinpulver, som vil blive brugt af op typen alt, hvad der kan være til at drysse på 

at kontrollere, hvad de kaldte 

Resource Øjeblikkelig Protein 

samme, hvis du spiser færdig suppe med op, så du bør tilføje mere protein. 

Hvis du køber færdiglavet kylling suppe, tilføje mere kylling på mix 

fiskesuppe, tilføje flere fisk osv osv 

Men proteinpulver bør tilsættes så meget som de gik eller blandes i vand og drikke. 

Kan være en ordreartikel hos nogle apoteker. 

Jeg bestilte min via nettet.

**25**

Søn 23 okt 2016 11:41 # 133

Der er sådanne proteinpuddinger af brand njie. 

Nogen, der ved, om det er godt for folk, der er vokset, tænkte bare på proteinet.

**26**

Ons 26 okt 2016 14:20 # 134

I morgen er det tid !!! Min søn fik maven i går (diarré) så nu er det lidt panik her. Forestil dig, om han smitter mig og operationen er annulleret?!? Vasket og sprinklet helt hjemme her. Og jeg bliver som regel ikke syg. Det ville have været forfærdeligt, hvis operationen blev stoppet! 

Er gået fra BMI 39 til BMI 34.5 under strømmen. -12,5 kg. Føles godt! Men jeg kan ikke se det selv. Mår maven det samme som før? Men jeg bemærker det på tøjet.

**27**

Ons 26 okt 2016 16:21 # 135

Ja de er gode sagde dietitianen.

**27**

Ons 26 okt 2016 16:21 # 136

Held og lykke, hvor spændende! Lova at rapportere her så hurtigt som muligt!

**29**

Ons 26 okt 2016 16:33 # 137

Hej. Jeg ville elske at springe ind i tråden! Har været på info i en gruppe og er også blevet kaldt en læge besøg 21/11. Dette er noget jeg tænker på dagligt og faktisk langt, men er bange for at blive stillet spørgsmålstegn ved og nægtet ... ved ikke hvorfor jeg tror det. Er der ngn i XXXX venter !?

**30**

Ons 26 okt 2016 17:06 # 138

Hej, jeg hopper lidt ind i tråden. Jeg har et BMI på ca. 45, men har aldrig seriøst betragtet en operation. Jeg ved det faktum, at jeg har en "spiseforstyrrelse", fordi jeg binge og sukker afhængighed, og hvis jeg gjorde en operation og gik ned partiet, det ville sandsynligvis bare ender med jeg (til sidst) går op hele igen på grund af mit forstyrrede forhold til mad og sukker. Det plus jeg er bange for operationer. Jeg forsøger at bekæmpe min vægt på andre måder. Så hvad ser du som operative er / skal du operere på dette? Er du bekymret for at holde vægten (omtrentlig) efter 1, 5 og endda 10 år efter operationen?

**31**

Ons 26 okt 2016 19:58 # 139

Jeg venter på dig! Fik i dag var via post, at jeg blev godkendt på en læge konference og i dag ringede til en dame, der sagde, at de blev kvittede ressourcer på XXXX i XXXX og spurgte om jeg var interesseret i at operere der, og jeg takkede dig! Skal hentes hjemmepapir om en uge så ved jeg mere. 

Min tur: 

6. juni VC besøg med prøveudtagning og henvisning til XXXX 

10. august Meddelelse til informationsmøde 

12. oktober Info møde XXXX 

Nu håber jeg, det går hurtigt, men hun talte med at troede, det var alligevel før jul! 

Nervøs men meget forventningsfuld!

**29**

Ons 26 okt 2016 20:57 # 140

Spændende, så er det tæt! 
Hvor lang tid med suppe inden du går op?

**27**

Ons 26 okt 2016 21:14 # 141

Det ser ud til at afhænge af, hvilken BMI man har. Jeg behøvede ikke at flyde, mens andre anbefalede 2 uger før.

**27**

Ons 26 okt 2016 21:16 # 142

Tillykke! Håber det går væk for dig da. 

Jeg ringede til klinikken den 29. september og fik tid til OP allerede i denne uge, men jeg takkede nej på det tidspunkt og vil blive betjent senere i november i stedet. Så det gik virkelig væk. Men det er privat og jeg skal betale for mig selv.

**31**

Ons 26 okt 2016 21:33 # 143

Tak! Hvor skal du blive opereret? Ved ikke så meget end bare, håber jeg får en dato kort så kan planlægge lidt. :)

**26**

Tirs 27 okt 2016 05:15 # 144

Hej alt nyt! 

Nu har jeg bruser min sidste dobbelte bruser og venter og venter. Klokken seks kommer taxaen! Nervøs men minded at dette vil gå godt. Hør mig, når jeg kan / kan.

**29**

Tirs 27 okt 2016 07:07 # 145

Her i XXXX flyder det i 4 uger før op ???? 

Held og lykke med din op i dag !!!!

**29**

Tirs 27 okt 2016 07:08 # 146

Betydning udråbstegn!

**25**

Tirs 27 okt 2016 08:39 # 147

26 

Held og lykke i dag, 

dejligt, du var nødt til at tænke dig selv om du var syg af familien. 

Masser af styrkende rammer til dej

**25**

Tirs 27 okt 2016 08:43 # 148

27 

Tak, jeg ved ???? 

Så kan du købe en sådan slags. 

Sælger de lidt billigere da jeg var på XXXX ???? 

Har du haft en ny tid, når du skal stå op?

**25**

Tue 27 Okt 2016 09:43 # 149

29 

Hvilken BMI har du, hvis du kan spørge. 

4 uger !!!! De lyder svært at flyde så længe. 

Jeg skal flyde i to uger før åbningen, som vil være i slutningen af ​​november. 

Jeg planlagde at flyde med Modifast, så kan du også spise pastaretter og Chocklad puddinger. 

Tænk, det bliver lettere for mig, hvis jeg får tygget et mål om dagen og ikke bare hælder i mig alt. 

Og slippe af med mig Chockled puddingen når søvn tændes. 

Så skal jeg gå ned 5.5kg op. 

Har allerede gået ned 4kg og startede ikke min strøm så det skulle gå uden problemer ????

**29**

Tue 27 okt, 2016 10:58 # 150

25 
Jeg har et BMI 37. 
Ja det er hård, men nødvendig i XXXX helt at reducere belastningen og størrelsen af leveren. Vil være en hård måned ???? 
Hvad har du for BMI?

**29**

Tue 27 Okt 2016 11:02 # 151

25 Hvor lang tid tog det for dig at få en ide om hvad du lavede på lægenes besøg?

**26**

Tors 27 okt, 2016 16:09 # 152

Hej! 
Nu er det gjort! Jeg var her kl. 18.45 og var klokken 8. Havde en dårlig tid klokken fire og fik morfin, men havde ikke brug for noget lige siden. Det moles og sårer lidt, men er klart håndterbart. Lidt groft i halsen og drikker vandet med vand. Har slet ikke vidst, at maven er særligt strakt, men har meget smerte i skuldrene. Min vidunderlige læge fik også min navlebrød, så det var to til en dag i dag. 
Har været helt træt (sove absolut intet uopmærksom), men de vågner en gang om ugen for at gøre en tur og blæse kæbe modstanden. Håber ikke at blive vågnet en gang om natten i løbet af natten. 
Jeg føler mig meget glad og stolt af mig selv, fordi jeg har forstået dette. Har ikke kunnet mærke dette før, så det er virkelig så dejligt! 
Alt i alt er dette en helt succesfuld oplevelse. Kæmp på andre!

**31**

Tue 27 Okt 2016 17:05 # 153

Åh, hvor vidunderligt er det nu gjort! 

Så jeg ønsker hurtig opsving hurtigt, og det vil være spændende at følge din første gang som nyopereret, hvis du vil dele med dig dvs. :)

**29**

Tirs 27 okt 2016 22:14 # 154

Åh så dejligt! Tillykke 26 ???? 
Håber du at spike på dig!

**25**

Fre 28 okt 2016 11:14 # 155

29 

Jeg havde 41,5 i BMI på tidspunktet for grædning. 

Vi var nødt til at møde kirurgen efter informationsmødet, som han derefter godkendte en og så fortalte han dig, hvor meget der skulle gå ned i vægten før åbningen. 

Han sagde, at jeg kunne bestille mig lige op, hvis jeg ville, men fordi jeg var i udlandet og rejste allerede, kunne bogen udsætte den. Du kommer ikke til at rejse til udlandet inden for 2 måneder efter åbningen. 

Så bookede du mig en uge efter ferieens afrejse ???? 

Sådan var min tur opdateret. 

Ring til sundhedscentret 14/3 

Fik en læge dag på sundhedscentret 7/4 

Hvor prøver blev taget. 

Opkald til ultralyd på grund af høje leverværdier. Ultralydet viste, at det helt sikkert skyldtes alle galdesten. 

Remiss sendt 7/6 

Bekræftelseserklæring 14/6 

4/8 Fandt ud af at XXXX ikke opfylder ventetid, XXXX overtager 

15/8 modtaget breve hjem fra XXXX og tid til infomøde 

31/8 tid informationsmøde på XXXX slutningen af november kirurgi 

**29**

Fre 28 okt 2016 17:05 # 156

Okay, så bliver det snart !! Spændende! 

XXXX siger, at de ikke sender videre til XXXX , selvom de ikke opfylder sundhedsgarantien, men gør de virkelig det? 
Det kan stadig være, at de kan klare plejegarantien ... Jeg ved det ikke. 
Går til en medicinsk vurdering 21/11, glæder mig til det. 

God weekend

**25**

Lør 29 okt 2016 08:14 # 157

Hvis de ikke accepterer sygesikringen, skal de videregive, hvis du ikke vælger at vente længere. 

Den medicinske vurdering, hvor er den, og hvad kan du finde derude? 

Jeg håber du behøver ikke at vente for længe. 

Når du først har besluttet dig for gbp, vil du gerne have det gjort allerede i går ????

**29**

Sø 30 okt 2016 13:08 # 158

25 : 

Vurderingen er vægtning og diskussion af metodik og opstilling af en til operation. Ja, du vil allerede blive betjent, så svært at vente! 

Har du startet flydende endnu? Eller var det første 2 v før? Nice is, så hårdt på float.

**31**

Man 31 okt 2016 18:55 # 159

I dag har jeg fået opkald til Pre-op tilmelding / læge besøg. Den 14/11 på XXXX vil det være næsten en hel dag med møder med læger, diætister, stofmisbrugere, prøveudtagning. Det føles som om det ikke kan være så langt tilbage til kirurgi! :)

**27**

Man 31 okt 2016 19:29 # 160

Hvordan har du det nu?

**26**

Tue 1 Nov 2016 22:29 # 166

Jeg er ret træt. Det er mest sandsynligt, fordi kalorieindtag er så lavt. Men også på operationen som sådan. Jeg sover meget, når børnene er i skole, men så er jeg sammen med dem. Nå, måske ikke skynde dig at overdrive alt for meget, men gå til legepladsen og spille hjemme er ikke noget problem. Jeg gik hjem den følgende dag. De ville se, at jeg kunne komme i væske og at jeg ikke var så dårlig. Det var dejligt at komme hjem, fordi jeg sov dårligt på hospitalet.

**26**

Tue 1 Nov 2016 22:31 # 167

Jeg har besluttet at kun veje mig en gang om ugen nu, om mandagen. Vægtet 119,5 dage Jeg begyndte at flytte, 108 på dagen og 106,7 i går.

**25**

Fre 4 nov 2016 13:53 # 168

26

De er rigtig godt arbejde.

**29**

Lør 5 Nov 2016 13:57 # 169

Du er velkommen til at høre, at du er okay! Ja, du har ikke for meget energi opbevaret efter at have flyttet tid og op. Tager sikkert et stykke tid, før du føler dig mere mousserende. 

Nu kommer det tættere på dig med 25 , spændende!

**29**

Lør 5 Nov 2016 13:59 # 170

Så er du gået meget, godt arbejde !!!!

**26**

Mandag 7 Nov 2016 08:21 # 171

Vejer i dag 
103,7 kg. -15,8 kg siden flystart! Føles absolut utroligt lige nu!

**32**

Mandag 7 Nov 2016 17:15 # 172

Jeg synes det er godt for dig at få hjælp, men desværre er ikke alle operationer tilbudt til alle. Jeg har selv gået ned fra 124,8 kg 168 cm juni 27, 2016 begyndte jeg vægt rejse i dag jeg vejer 98,4 kg, jeg føler, at indvejning på samme vægt som dig, så længe personen blev indskrevet på fedme på XXXX personen fik hjælp fra en læge, diætist og psykolog personen havde ingen kirurgi blev de alle kaldt til et møde, hvor informerede læger at kirurgi alene tilbudt nogle andre koster ikke kontrol sæt. Ventetiden til operation var tre år denne person begyndte at spise kost pulver under kontrollerede forhold og derefter normal kost med møder i diætist mm og begyndte at tabe sig og lykke med vægttabet.

**32**

Mandag 7 Nov 2016 17:19 # 173

Jeg har ikke fungeret med viljen bedre kost motion har taget mig, hvor jeg er i dag.

**26**

Mandag 7 Nov 2016 17:59 # 174

32 
Det er fantastisk for dig, men det er utroligt usædvanligt at lykkes, så godt som du har gjort. At tabe kan alt andet end opholder sig der er en anden fem. Jeg har en vilje af stål og har gået ned over 30 kg 4 gange ved hjælp af forskellige metoder. Det lykkedes aldrig at blive der. Man bør være opmærksom på, at det ikke er så enkelt som kalorier ind og ud. Der er et komplekst samspil mellem hormoner, følelser, stofskifte - der kan faktisk være helt forkludret af yo-yo slankekure. Som psykolog på XXXX sagde: "Fordi Usain Bolt kan løbe 100 meter på 9,58 betyder det ikke, at enhver der arbejder hårdt, vil kunne. Jeg kan være så utroligt træt, at der ser ud til at være en "god måde" at tabe sig - alene og en foul og fræk operation. Jeg bedre tager de odds, som operationen tilbyder - vedvarende vægttab og vægtstabilitet på lang sigt. Men jeg ville elske at undgå at blive skammet af mennesker, der ikke tror på metoden.

**31**

Mandag 7 Nov 2016 19:45 # 175

Dejlig 26 ! 15 kilo er fantastisk! Hvor længe flygtede du? I en uge møder jeg en kirurg, anæstetiker osv., Håber at få operationen snart !!

**26**

Mandag 7 Nov 2016 21:04 # 176

Jeg begyndte at flytte mandag 3/10. Opereret 27/10. Så lidt over tre uger. Vær på 40 års fødselsdagsfest og spist og drak lidt vin, så jeg gjorde nogle ekstra dage til dette. De gjorde ikke nogen begrænsninger eller behovet for mig, jeg ville gøre mindst to uger. Men jeg kørte lidt ekstra.

**32**

Mandag 7 Nov 2016 22:19 # 177

Nogle gange tager det tid, jeg gik også ned før, men gik op, da jeg blev gravid efter fødslen til fire børn, så træde kilorna ud over min tidligere fedme. Man kan klare at gå ned med hjælp fra psykologen dietist og lære at spise rigtigt handler det om at spise rigtigt og flytte. Selv kirurgi kræver, at du forsøger at komme op igen, hvis du laver fejl, alt handler om at ændre din tankegang, selvom du arbejder. Spise kost pulver ja så du går ned, men det har ikke ændret den tankegang, når du indtaster det samme barn igen tage på i vægt for mænd ikke har lært, hvordan man spiser ordentligt eller ændret holdninger til mad, har jeg fulgt folk i deres vægt kæmpe en kvinde der vejer 140 kg var lige så høj som mig, gik hun ned mig selv i dag, hun vejer normalt hun ændret hendes tankegang til mad og lærte at spise rigtigt.Din vrede Jeg forstår ikke jsg har ikke lød på operation. Jeg skrev bare, at alle ikke kan få det forstyrret, hvis du ikke kan betjene operationen selv privat. Den person, jeg kender, fik ikke operation med en diætist og psykolog, personen tabte sig og returnerede ikke alle sårene igen. Jeg valgte at gøre det selv, jeg ønsker ikke at drive jeg har lært at spise bedre og bedre mad bevæge mig mere. Det var mit valg, jeg ønsker ikke at gennemgå en operation. Derfor kæmper jeg alle, vi har et valg i vores liv, jeg valgte en anden måde. Jeg dømmer ikke nogen efter eget valg, men en ting er sikker på at veje 124,8 kilo, jeg vil aldrig gøre gladere for alle sammen med digDen person, jeg kender, fik ikke operation med en diætist og psykolog, personen tabte sig og returnerede ikke alle sårene igen. Jeg valgte at gøre det selv, jeg ønsker ikke at drive jeg har lært at spise bedre og bedre mad bevæge mig mere. Det var mit valg, jeg ønsker ikke at gennemgå en operation. Derfor kæmper jeg alle, vi har et valg i vores liv, jeg valgte en anden måde. Jeg dømmer ikke nogen efter eget valg, men en ting er sikker på at veje 124,8 kilo, jeg vil aldrig gøre gladere for alle sammen med digDen person, jeg kender, fik ikke operation med en diætist og psykolog, personen tabte sig og returnerede ikke alle sårene igen. Jeg valgte at gøre det selv, jeg ønsker ikke at drive jeg har lært at spise bedre og bedre mad bevæge mig mere. Det var mit valg, jeg ønsker ikke at gennemgå en operation. Derfor kæmper jeg alle, vi har et valg i vores liv, jeg valgte en anden måde. Jeg dømmer ikke nogen efter eget valg, men en ting er sikker på at veje 124,8 kilo, jeg vil aldrig gøre gladere for alle sammen med digJeg dømmer ikke nogen efter eget valg, men en ting er sikker på at veje 124,8 kilo, jeg vil aldrig gøre gladere for alle sammen med digJeg dømmer ikke nogen efter eget valg, men en ting er sikker på at veje 124,8 kilo, jeg vil aldrig gøre gladere for alle sammen med dig vægttab

**25**

Mandag 7 Nov 2016 22:43 # 178

32 

De 26 mener, at denne tråd handler om gastrisk bypass. 

En vej, som vi alle her vil gå og vil gå, når vi allerede overvejer, forsøgte vi alt andet. 

Og det er et hjælpemiddel til os. 

Vi ved godt, at dette er et værktøj, og at vi får arbejde hårdt. 

Disse er ikke beslutninger, vi har taget direkte fra luften, men den e gennemtænkt både 1, 2 og 3 gange 

havde vi ønsket at fortsætte vores yo-yo slankekure, så vi ikke har nogen inde i denne tråd :) 

Dejligt job ved dig og held og lykke med din kost. 

Jeg håber du klarer også at holde vægten bagefter.

**26**

Mandag 7 Nov 2016 22:52 # 179

Selvfølgelig bliver jeg pisset af, hvis du kommer ind i denne tråd og snakker (for det meste), at det handler om at ændre tankegang og spise rigtigt for at tabe sig. Tror du ikke, at vi alle her har testet det, hvor mange gange? Det er undertonerne om, hvordan du udtrykker dig selv, hvilket er ubehageligt. Lav din egen tråd om vægttab og diskutere med andre, der gør som du gør. I stedet for at komme ind her og få folk til at føle sig potentielt dårligt, at de har taget eller vil gøre denne livsbeslutning.

**32**

Tue 8 Nov 2016 06:05 # 180

Mange tak til dig selv.

**25**

Tue 8 Nov 2016 08:21 # 181

Ja, de nærmer sig, min strøm begynder i morgen. 

Kan du se, hvordan man håndterer det? 

forudsat at du vil være sulten generelt på et dårligt humør, men vi vil se ????

**31**

Tue 8 Nov 2016 21:04 # 182

Held og lykke med strømmen! Det vil nok gå godt, men selvfølgelig de første par dage (tror jeg). Hvornår bliver det operation?

**26**

Tue 8 Nov 2016 23:59 # 183

Held og lykke med 25 ! Det bliver fantastisk! Husk, at det er en begrænset tid. Så har du aldrig brug for mere pulver!

**31**

Ons 9 nov 2016 16:19 # 184

Nu har det været et par dage med influenza for dig, føler det sig okay eller er det vanvittigt?

**25**

Ons 9 nov 2016 17:21 # 185

Den første dag i flyvningen i dag. 

Druckit som Tusan og kyssede der efter endnu fået verdens migræne. 

Ved ikke, om de er fordi jeg flyder eller hvis jeg har det alligevel. 

Eran opmuntring hjælper virkelig meget, 

du bliver pepped. 

26 : 

Hvad kan du drikke under flow ??? Kan du drikke coca cola nul ved en særlig lejlighed? 

Eller bare vand, kaffe og te, der gælder? 

Vi er i XXXX på ferie, at hele familien blev sulten i indkøbscenteret, de blev pizza og varm makrel, og jeg sad der fint og mens dreglet løb langs mungotten,

**31**

Ons 9 nov 2016 19:47 # 186

Åh, stærk at være på ferie og starte! :) Tænk de første 2-4 dage er det værste, så det føles bedre og bedre .. :) Jeg møder en kirurg og diætist, og så på mandag, vær så spændt! Spekulerer på, om jeg kender dato for kirurgi da? Vil du få det gjort nu !!!

**26**

Ons 9 nov 2016 20:38 # 187

25 : Du kan helt klart drikke Cola nul! Indeholder ingen kalorier! Fokuser på det snart! Vidunderligt! Jeg bliver post-up kort efter to uger. Føler at tiden flyver.

**25**

Ons 9 nov 2016 21:34 # 188

31 : 

Jeg har været en uge med ferie allerede så fanget skubber mig nogle churros :) 

men nu e-mailen alvorligt. 

Du kan annoncere senere hvordan det går på mandag, 

Ja virkelig håber du får ud af tid, og at det bliver snart. 

26 : 

Åh, okay, ja, jeg har det godt, jeg kan i hvert fald smide mig selv. 

Modifast pasta er alt andet end godt, her i XXXX de har ikke Modifast, vil du spiser dem, jeg bragte hjemmefra som desværre eh alt for mange kasser med pasta 

jeg tæller ned dage 

Va allerede for to uger siden, din op, ja tid hår virkelig hurtigt. 

Er du fuldstændig restaureret eller stadig træt? 

Hvordan virker det med mad nu? 

Hvordan ser en dag ud på mad?

**26**

Tors 10 nov 2016 00:37 # 189

Jeg har det fint Stadig lidt træt, men det er fordi jeg spiser for lidt. Skal spænde mig med det. Jeg er ikke sulten, og så er det så nemt at glemme ... 
Jeg spiser normalt havregryngrød med sukkerfri syltetøj eller banan til morgenmad. Så kan jeg spise en crackle til frokost, og så når børnene spiser, spiser jeg med dem, hvis jeg kan. Vi spiste spaghetti og kødsauce i dag, så jeg gjorde succinin nudler og spiste saucen. Forsøger at få en masse avocado og makrel, der er energieffektiv. Spis en masse æg også og keso.

**31**

Tors 10 nov 2016 19:25 # 190

Skal være en mærkelig følelse endnu for at føle at spise LITE og have det svært at få nok! Så jeg har ikke kendt i mange år. Vil være spændende! :) Det er okay, begynder du at føle noget på din krop og tøj, som du går ned eller er den mest forfærdelige, der viser hidtil? :)

**26**

Tors 10 nov 2016 21:36 # 191

Ja, jeg får tøj, der ikke varer i lang tid. Men det er mærkeligt, fordi jeg kender mig selv så stor. Selvom jeg ikke er det. Det er godt hjernen, der er svært at holde op med, formoder jeg. Hvordan er strømmen 25 ?

**25**

Fre 11 nov 2016 07:01 # 192

Advokado til makrel var gode tips, de skulle lægge på hukommelsen. 

tænk at sen føler sig komfortabel ikke konstant sulten. 

Så i går var de lidt hårdere, ingen hovedpine, men stadig sultne til at tænke på mad. 

Når du laver børnenes mad så måtte du kæmpe, fordi der ikke ville være en lille smule ned. 

Du bemærker, hvor meget det falder ned her for at være, når du skal stræbe efter at få det til at ske.

**29**

Fre 11 nov 2016 21:42 # 193

Snart er du 25 ! Ja, du kan ikke forstå, hvordan det føles at være ikke sulten eller skør. Det er for ofte. 

Tager nok tid til selv at hænge ud med invitationen du leder efter. 

Hav en god weekend!

**26**

Lør 12 Nov 2016 12:06 # 194

Det har været to hårde dage. Det har været en god dag hidtil, men i går spiste jeg let stegte hamburgere (jeg lagde det i husholdningspapir bagefter for at slippe af med gryden) og kogte blomkål og broccoli. Gjorde meget dårligt bagefter. Og derefter har jeg følt mig meget dårlig efter måltidet. Jeg spiste røræg i går morgen og kogte æg om aftenen og følte pyton bagefter. Ulkade selv om aftenen. Lige nu er jeg ikke vild med noget. Vil ikke engang tænke på mad. Får vand uden problemer heldigvis. Jeg troede, jeg ville vende tilbage til at spise mere væske, skrive fil og så og se, hvordan det går. Bliver lidt iøjnefaldende, fordi det gik godt alligevel indtil i går, men jeg tror det kan gå op og ned. Har også læst, at mange bliver følsomme for kød, og at det er individuel, hvordan man modstår mad.Kan tage en pause fra æg og kød. Uanset hvad ... hvordan har du det? 25 ? Hvornår er det operation for dig?

**26**

Sø 13 nov 2016 00:14 # 195

I dag gik det bedre. Kunne spise både filen og den skarpe ost med ost. Pyha! Vent venligst et stykke tid med æg.

**31**

Sø 13 nov 2016 10:54 # 196

26 , usch, hvor svært! Rart at føle sig bedre nu! Antag at det er meget individuelt, hvad du kan gøre og ikke bagefter. Jeg håber du ikke får flere så dårlige dage nu! 

I morgen er det en kirurg for mig, spændende! Håber jeg vil vide mere, når operationen skal være og så .. Vidste du datoen, hvor du var der eller i bogstaver hjem bagefter?

**26**

Sø 13 nov 2016 11:42 # 197

Jeg reserverede min dato med receptionisten. Jeg gjorde min handling privat, så måske er det en anden ting. Jeg måtte vælge en dato, der passede mig.

**25**

Sø 13 nov 2016 17:33 # 198

31 

Hvordan gik det i dag? 

Jeg var nødt til at bestemme datoen selv, når ja, hvor der 

24 er klokken bare en måned før jul ???? 

26 

De kan have for tidligt for den mad? 

Fast ja, bare gætte ???? 

Kan være bedre, hvis du prøver dem senere, hvis du tør at udsætte dig selv. 

Eller som du sagde, må din mave ikke tolerere det længere

**25**

Sø 13 nov 2016 18:25 # 199

31 

Bare indså, at de var søndag i dag, indtil i morgen var du der ???? 

Ja, jeg kan sige ja, jeg er på ferie. 

Ser frem til IMORGON for at høre, hvad de sagde 

????

**27**

Sø 13 nov 2016 18:32 # 200

Nu er jeg også slewed, fungerede i den uge, der gik. Operationen gik godt, ikke underligt overhovedet. Jeg havde lidt smerte lige efter, og den første dag efter åbningen var lidt krydret, men det var meget lettere end jeg troede. Men jeg var heldig og led ikke af nogen mærkelige bivirkninger, gasskader osv. Jeg gik hjem efter to dage, og det har også været godt herhjemme. Jeg spiser ikke længere smertestillende eller tabletter mod kvalme og føler mig næsten vanvittig god. Så er det selvfølgelig ikke altid tilfældet. Jeg har været på "dumping", og jeg har ikke haft toppen hele tiden. Men i det hele tror jeg, jeg har det bedre end mange andre gør. 


 


Nu længes jeg efter udgangen af ​​næste uge, når jeg kan begynde at spise pureadfood, fordi jeg virkelig er ligeglad med supper og væsker. Næsten alt, der går uden problemer, er Proviva, men det er så himlen sødt. I dag har jeg drukket let og det var så godt og frisk og surt at jeg næsten blev religiøs på kuppet! 

Jeg vejede i sidste søndag og nu i dag igen, og bølgen viste mindst 5,0 kg.

**26**

Månen 14 Nov 2016 13:42 # 201

Hej 27 og tillykke! Sikker på, det er godt, når du er færdig! Kan du spørge, hvordan man bestemmer sig på ærmet? Interessant nok synes jeg at vide, hvordan beslutningsprocessen har været. På XXXX lover de stadig GBP (der er en undersøgelse, der sammenligner metoderne). Hvordan skete der, når du dumpede? Hvad dumpede du på / hvorfor dumpede du og hvordan fik det det? 
Okay!

**27**

Månen 14 Nov 2016 13:54 # 202

Hej og tak! Ja, det er rart at have det gjort og rart at jeg bliver nødt til at føle mig så god. 

GBP føles som en større operation med flere risici og bivirkninger. Det er det, jeg læste i det mindste. Lægen på klinikken var jeg sagt det samme, og for mig var der aldrig tanken om GBP uden at være en ærme eller slet ingenting. 

Dumping var ikke så alvorlig. Lidt blodtryksfald men ingen dykker helt ned så at sige. Lidt kvalme og diarré. Jeg har et par ting nu, at jeg absolut ikke kan spise og flyder ud på den anden side direkte (uden et dump, det krøller bare til maven og kommer ud som diarré). Tilgiv mig at være så grafisk, men jeg ser ingen grund til at elske sandheden;) 

Jeg er lidt bekymret over, at jeg føler mig så god, og jeg synes at være i stand til at spise ganske store mængder uden at blive såret eller føle sig nogen stop overhovedet. Selvfølgelig har jeg ikke testet i mig mere end 1,5-2 dl pr. Lejlighed men stadig. 

Jeg længer efter puréperioden. Jeg vil tygge! 

Tror du, at 5 kg lyder lidt i en uge?

**26**

Mandag 14 Nov 2016 14:13 # 203

Virkelig ikke! Det ligner meget! Jeg er gået ned fra 108 på dagen for drift til 102,2 i dag. Det er derfor om to uger og tre dage. Jeg synes det er okay. Ønsker huden at hænge ud så meget som muligt, og jeg ved, at jeg vil gå ned. Jeg jager ikke på alle kiloner på samme måde som jeg har pulveriseret og været på kostvaner. Det er meget flot. Kan du spørge, hvor meget du vejer / BMI? Jeg havde BMI 39 før pulverstart og er nu nede på 33.

**26**

Månen 14 Nov 2016 14:16 # 204

Forresten spurgte jeg min sygeplejerske om det, fordi jeg også følte mig meget god (et par dage var det en smertefuld irritation), men hun sagde bare, at du skulle være glad. Vi føler så godt som de håber at gøre. Og ligesom du siger, har du ikke spist mere, end du burde. Jeg føler ikke nogen klar sult eller mætning endnu, men hun sagde, hun kunne tage et stykke tid. Med tiden vil kroppen lære de nye signaler.

**27**

Mandag 14 Nov 2016 14:32 # 205

Jeg startede på 94,4 og vejer nu 89,4 nu. BMI 33.85 og nu 32.06 

Jeg ønsker virkelig ikke at jage vægten og i stedet for at tænke "smallere" tænke "sundere" for mit helbred er, trods alt, netop fordi jeg har betalt for det selv. Ikke desto mindre vil jeg gerne vide, at det er i "rigtigt" tempo, så intet har været forkert. Måske ville det have været lettere, hvis du skulle blive værre. Weird.

**26**

Mandag 14 Nov 2016 15:43 # 206

Jeg har også betalt for mig selv. Jeg føler mig ikke særlig stresset overhovedet. Jeg ved, at jeg vil tabe, forudsat at jeg tager mig af mig og spiser ordentligt. Og åh min Gud, du har faldet 5 kg på en uge! Det er utroligt meget!

**31**

Man 14 nov 2016 16:07 # 207

Nu har jeg været der! Er nu indskrevet og har mødt en kirurg, anæstetiker og diætist. Skal flyde i 4 uger, men har ingen dato end kirurgi. De tilhører. Forhåbentlig bliver det hurtigt! Kirurgen troede, at vi ville gøre GPB på mig (diskuterer begge muligheder), så nu er det besluttet! Alle var super hyggelige og det føles godt! 

????

**26**

Mandag 14 Nov 2016 16:22 # 208

Dejligt !!!! Skal du begynde at flyde nu?

**31**

Månen 14 Nov 2016 16:49 # 209

Ikke så snart jeg har driftstid, ved jeg, hvornår jeg skal flyde. Men håber det bliver snart !!! :) Vil du vide nu, så du kan planlægge ..

**25**

Mandag 14 Nov 2016 21:22 # 210

31 

Hvad godt, så går de på? 

Jeg var nødt til at vælge ærme eller GPS 

jeg valgte GPS på tanken han ja havde ret. 

Ærmer har ikke været så længe og er derfor ikke så meget et grundlag, hvordan de ser ud i fremtiden, 

ret der bestemt min beslutning. 

Men de er de samme, bare gør hvad der passer til dig selv. 

Men som tidligere nævnt kan der være flere bivirkninger på gps. 

26 : 

Jeg tror ja, bliver sulten for at blive sulten for at få sværere med strømmen hver dag, de bliver normalt ikke lettere længere. 

Eller ja ligesom e tilbage til bare ????

**26**

Mandag 14 Nov 2016 21:29 # 211

25 : 
Nej, jeg oplevede det ikke lettere og lettere. Jeg følte mig meget op og ned. En dag var jeg sulten på alle andre dage, jeg tænkte næsten ikke på mad. Hold på! Du er snart i mål!

**25**

Mandag 14 Nov 2016 22:03 # 212

26 : 

Så ja, så tager de kun en dag og tæller ned.

**25**

Tue Nov 15, 2016 11:11 # 213

Så har vi lavet et opkald efter en uges Modifast. 

Minus 7,1 kg om en uge !!!!! 

Syk glad 

Så i alt -10,6 siden jeg gik til informationsmødet og fik min tid.

**31**

Vores 16. november 2016 13:18 # 214

Nu har jeg haft driftstid! 20. december vil det være! :) Flying start mandag .. ????

**31**

Vores 16. november 2016 14:50 # 215

Hmm .. hvor kom alle spørgsmålstegn fra ??????

**26**

Vores 16. november 2016 15:22 # 216

Wow 25 ! Der var utroligt kilo! 

Dejligt med oppetid! Hvad en god julegave til dig selv! Held og lykke med strømmen!

**25**

Vores 16 nov 2016 17:26 # 217

31 

Haha de med ??? Har du nogensinde spekuleret på, hvor de kom fra, da du skrev. 

Hvor sjovt har du tid, lidt mere end en måned bare ???? 

Hvilket mærke vil du gerne flyde med?

**27**

Vores 16. november 2016 18:08 # 218

Hver gang du laver et smil, bliver det tvivlsomt i stedet. 

**31**

Ons 16 nov 2016 21:22 # 219

Jeg skulle køre med Modifast, jeg synes det er godt .. Føler skræmmende, men spændende! Men julen bliver sådan ... Haha!

**27**

Tue 17 Nov 2016 08:17 # 220

Jeg skiftede fra flydende mad til "let" tidligere end skemaet. Jeg var nødt til at abe på supper, det virkede virkelig ikke. Nu spiser jeg faktisk næsten som før operationen og får toppen. Beløbene er naturligvis betydeligt mindre og kalorieindtaget også. Og det tager et stykke tid at spise, men for mig var suppeperioden helt sjælkrævende. 

**19**

Tirsdag 17 november 2016 22:42 # 221

Hej! 

Enhver der lavede UP i XXXX , og muligvis en ærme måske?

**29**

Fre 18 nov 2016 06:55 # 222

Spændende at læse om dig og jeg så længe. Går på min læge besøg forud for mandag, så rejser jeg mig op og håber det vil ikke være for lang tid at gå op ???? 
Tog det 3 måneder for dig? 

Hav en god fredag ​​!!!

**27**

Fre 18 nov 2016 07:44 # 223

Min første kontakt var 29. september. Jeg er oppe: snart siden 2 v Men jeg betalte mig selv. 

**31**

Fre 18 nov 2016 09:20 # 224

Jeg blev først godkendt i medicinsk konference i oktober, så mødte jeg den kirurg, anæstesilæge og diætist på mandag og har nu fået den op-tid december 20! :) Hvilket hospital skal du?

**29**

Fre 18 nov 2016 19:10 # 225

Okay, det er væk! Så rart! 

Jeg bor i XXXX og kommer ind i XXXX .

**29**

Lør 19 Nov 2016 16:15 # 226

27 - fordi jeg spørger, hvad det koster at betale det selv? Hvis du ikke vil svare, er det okay.

**26**

Lør 19 Nov 2016 17:32 # 227

Snara: Jeg har betalt 89 000: - 
Dette inkluderer alt efterpleje og opfølgning i to år. Jeg valgte dette fordi kirurgen er en af ​​de bedste i Sverige på gastrisk bypass, og jeg ville bare have ham til at fungere. Jeg har også modtaget sit private telefonnummer og kan ringe til ham, hvis jeg har brug for det. Sikker på, at det er dyrt, men jeg er villig til at betale dyrt for den sikkerhed.

**27**

Lør 19 Nov 2016 17:37 # 228

Nu er der måske to svar, men jeg kan ikke se det andet svar, jeg sendte for 5 minutter siden. 

Jeg betalte 75tkr. Et års opfølgning.

**25**

Lør 19 Nov 2016 21:16 # 229

Min op blev flyttet eller flyttet tilbage måske bliver de kaldt 

Vil være onsdag i stedet for torsdag. 

Du, der allerede står op for noget andet, du synes, er ekstra vigtigt at pakke op med sygdommen? 

Jeg skulle pakke min taske allerede i morgen ????

**33**

Lør 19 Nov 2016 21:23 # 230

Hej! Jeg har stort set bestemt mig for en ærme. Men er meget bekymret. Føler så skræmmende at drive en frisk madpose. 

Har nogen hørt om eller kender nogen, der beklager sin ærme? Jeg har ledt efter, men det ser ud til, at alle er glade, men nogle har ønsket at gå ned mere selvfølgelig eller gå op i vægt efter et stykke tid.

**27**

Lør 19 Nov 2016 21:27 # 231

Jeg var på en privat klinik, og de havde døde senge, ægte dyner og puder, så jeg tog ikke noget med det. Hvis jeg havde lavet mit sædvanlige hospital, ville jeg have taget min egen pude alligevel. Hvor jeg kom op var der også en fluffy badekåbe. 

Men der var et par ting, jeg var virkelig glad for at jeg bragte med mig, og det var 
- uldstrømpe (knæestrømper). Jeg havde dem iført mig ovenpå, og det var så rart at stikke rundt i dem siden 
- En tynd og yderst beskidt fleecefilt, der var dejligt at have i seng 
- Mobil + Oplader (selvfølgelig). Men jeg havde også telefonen læsset med lydbøger, som jeg syntes var hyggeligt. 
- Virkelig behageligt, at mit tøj tæmmer rundt i dagene efter. 

Og selvfølgelig er dine sædvanlige lægemidler.

**26**

Lør 19 Nov 2016 22:42 # 232

Hej! 

Jeg synes det vigtigste er, at du er tilfreds med din beslutning. Der er risici og fordele / ulemper ved begge teknikker. Jeg følte mig sikker med bypass. Der er nu en undersøgelse i Sverige, der nu sammenligner bypass og ærme. Jeg troede, det var en større aftale med ærmet, mere drastisk at fjerne meget, og der er nogle andre uklare ting rundt om ærmet. Jeg læser her, hvor min læge ( XXXXX ) taler om metoderne: 
XXXX 
Men som jeg sagde, tror jeg det vigtigste er, at du føler at metoden passer til dig!

**25**

Sø 20 nov 2016 09:09 # 233

27 : 

Tak, ja, ja, lidt mere, hvad du skal pakke

**26**

Sø 20 nov 2016 09:37 # 234

25 hvad spændende! Forestil dig, det er snart tid! 
Jeg siger også mit tøj. Virkelig blød og stor. Maven er hævet og ømt overhovedet, tænk på bløde trusser og bløde bh. Jeg havde hjemmesko med også og iPad (de havde wi-fi) så jeg kunne netflixa. Nice luksus shampoo og hudcreme kan være rart. Du bliver super tør af descutanen, og dit hår bliver så godt som muligt.

**29**

Sø 20 nov 2016 13:03 # 235

Tak for info 26 ! Ja, det er absolut det værd, selvom du satse! 

Held og lykke indtil nu 25 !!! Så spændende! 

Jeg har selv min læge besøg i morgen, føler mig lidt nervøs. Bange for at sige, at jeg ikke kan komme op ... men samtidig synes jeg det er klart, de siger ikke! Har et højt BMI og mange risikofaktorer.

**31**

Sø 20 nov 2016 17:29 # 236

Held og lykke til 25 ! Spændende! Opdater os så snart du kan .. :) Jeg begynder at flyde i morgen .. ????

**26**

Mandag 21 Nov 2016 13:00 # 237

Vejer i dag! 100,5 kg. -19kg siden flystart!

**31**

Mandag 21 Nov 2016 17:03 # 238

Smuk !!! Helt fantastisk! I dag er min første flydende dag farvel, hvad jeg er sulten og døsig. Håber det bliver bedre i løbet af få dage!

**29**

Mandag 21 Nov 2016 17:56 # 239

Spændende 25 ! Held og lykke! 

26 - Godt job! 

I dag er jeg blevet oprettet til operation! Føles så godt! Vil gøre en ærme forhåbentlig v 4! Så glad!

**29**

Mandag 21 Nov 2016 17:57 # 240

31 , ja de første dage er forfærdelige, ser ikke frem til 4 v flow .... huamej.

**19**

Tis 22 nov 2016 07:58 # 241

Hej alle! Her er en, der ønsker at 

blive med i gruppen. Jeg sendte min egen henvisning via 1177 til mit hospital i midten af ​​oktober. Fik kold læsning i sidste uge for at møde lægen ved kirurgens modtagelse og til gruppefoden. 
Nu er jeg så nervøs, at de skulle sige nej, nu at alt er så rigtigt, og jeg har et opkald. Og meget bange for at få et ja, fordi så vil jeg helt sikkert aflivet Jeg er så nojjig til, haha: D vil gerne gøre et ærme, men læs videre, hvis de to interventioner er enige GBP også, men min vigtigste ønske er ærme. 

Pas på dig og håber du OPa er god!

**31**

Tirsdag 22 nov 2016 17:45 # 242

Dag 2 med flow, føles lidt bedre i dag faktisk! 

Har en presserende chef, der besluttede at sympatisere med mig, så vi kan anspore hinanden. Grav i kraniet, men lyver ikke sulten i dag! Kom selv, fordi jeg savnede eftermiddagsmålet, så jeg kan tage det i aften.

**25**

Tirsdag 22 nov 2016 20:40 # 243

Ja, jeg er så nervøs for morgendagen. 

Har været op hele dagen og tænker ikke på det, men nu hvor børnene sover, ja, det er så nervøst. Og bare tænker på det værste tænkelige. 

Må tænke på at tænke over alle de kramper, jeg har, og hvorfor jeg gør det her

**31**

Tirsdag 22 nov 2016 20:48 # 244

Åh, er det i morgen i morgen? Jeg ønsker dig held og lykke! Du er velkommen til at opdatere os om, hvordan det virker!

**25**

Tirsdag 22 nov 2016 21:29 # 245

Du er velkommen i morgen, mange tak. 

, vil holde dig opdateret

**27**

Tis 22 nov 2016 22:16 # 246

Velkommen her!

**26**

Ons 23 nov 2016 07:41 # 247

Velkommen 19 ! 
Held og lykke for nu 25 ! I morgen er det en måned siden min operation! Skør, hvor hurtigt går tiden!

**25**

Ons 23. november 2016 16:47 # 248

Hej, jer alle 

kom op klokken 12 i dag, 

jeg er uhøflig god. 

Læg og gnid lidt vand på 30 ml, som skal opdeles i tre gange meget små snegle. 

I morgen kl. 14-15 bliver du trykt 

så langt så godt. 

Glad for at de endelig blev færdige for at se mig foran mit faldende fald.

**31**

Ons 23 nov 2016 19:31 # 249

Dejlige 25 ! Rart at høre, at det gik godt! Glad du er ikke så slemt og så .. Jeg længer nu!

**25**

Ons 23 nov 2016 20:34 # 250

Jeg har mere smerte i mit hoved end jeg forestiller mig. 0 har heller ingen problemer med gassen. 

Men så snart du går, er det jo dårligt. 

Så de går ned i seng for at lægge sig ned igen. 

Jeg har 4 værelser kompis, så ja en pige får tid til at gå med vores tallerken så ee nice

**27**

Ons 23 nov 2016 21:13 # 251

Åh, hvad mareridt at dele med fire andre, ville jeg nok aldrig have gjort det. Håber du føler dig bedre snart, har du faldet nu? Bed om det ellers, jeg kunne virkelig godt lide det, men det blev bedre straks, jeg kom ind i min væske via en dråbe. 

**29**

Ons 23 nov 2016 22:51 # 252

Dejligt at høre det op gik godt ????

**29**

Ons 23 nov 2016 22:55 # 253

19 , være spændende. 

Jeg blev mødt læge på mandag - jeg er nu oprettet til en ærme. Vær ok at vælge for min del. Har tidligere været på gruppeinfo. 
Hvor skal du lave dig selv? 
Med venlig hilsen 
29

**19**

Ons 23 nov 2016 23:12 # 254

Hvor dejligt du skal vælge! Jeg skal gøre min operation her i XXXX, hvor jeg bor. Vid, at dem, der udfører ærme her, men ikke ved, om du kan vælge, eller hvis lægen beslutter, hvad der er bedst for mig, men jeg håber, at jeg får mit ønske igennem, ellers takker jeg simpelthen o modtage en bypass naturligvis  være på holdet info Den 6., o mødes med lægen samme dag. Håber på et JA!

**25**

Tirsdag 24 november 2016 01:03 # 255

27 : Ja, dråben er lige afsluttet, så lige nu, ja ingen, men se hvordan de føler sig i morgen. Jeg vil forsøge at sove, men e for krydret. 

For at dele værelser synes jeg faktisk at være godt, efter de sidste tre fødsler, når du altid har haft en værelsekammerat. Det er dejligt at tale lidt om, hvordan vi føler, hvor vi har problemer osv. Haha at antydse hvad vi skal spise og derefter bagefter. 

Jeg ser dig i hvert fald for youghurteb til morgenmad i morgen :) 

Hvor længe spiste du supper og kiggede op?

**25**

Tirsdag 24 Nov 2016 07:43 # 256

Vi er to her som gbp og to, der lavede ærmet i rummet. 

Vi er gbp at være glade, men ærmerne er bare ikke gode. 

Du skal bære så meget værre efter ærmet.

**31**

Tue Nov 24, 2016 21:23 # 257

Har du været hjemme end 25 ? Hvordan har du det

**25**

Fre 25 nov 2016 13:23 # 258

Ja, jeg kom hjem i går. 

Føles ret godt, tager Alvedon hver fjerde time. 

Men har været på en tur for at gå på en til sent, kun 15 minutters gang, indtil videre. 

De er et fuldtidsjob for at få al "mad" til væske, 

men ja, jeg er overrasket over at se dig så begejstret for at stå op på dine ben efter operationen. 

Den mellemste barn sagde ja hva sooooo cool for alle af plast på min mave hahaha 

Nu e supper 2 uger forude, før de så blive puré i 3 uger på siden begyndelsen af almindelig mad. 

Hvordan er dit flow 31 , 

Er det stadig svært, eller de flyder på.

**26**

Fre 25 nov 2016 15:35 # 259

Dejligt at alt går godt 25 ! Jeg var heller ikke dårligt. Pyha. Men det er rigtigt et fuldtidsjob for at komme ind og drikke! I betragtning af at du heller ikke skal drikke på måltider, er det en konstant kamp at komme ind som du burde. Jeg håber at være under den 100. linje på mandag. Havde været fantastisk. God weekend alle sammen!

**31**

Fre 25 nov 2016 18:27 # 260

Det er ret godt faktisk! Men i dag har jeg været på hesteshowen, så det er hvordan du blev skør ... men jeg har taget mig af fristelserne! 

Hvad synes du? Den næste lørdag er Sambons søn 18 år gammel, og vi har længe planlagt at gå ud at spise på hans yndlingsrestaurant. Hvis jeg ser, at jeg er hjemmeindrettet (-6,5 i 4 uger) tror du jeg kan tage en eller to retter da? Det er Tapas, så det er små retter, normalt spiser jeg normalt 5. Føles så kedeligt, at jeg ikke kan ledsage ham og han ..

**26**

Lør 26 Nov 2016 14:08 # 261

Jeg lurede mig selv i dag, men jeg har virkelig en vejdag om mandagen. Skalaen viste 99 kg! -20,5 siden 3/10. Så skynd dig, fantastisk!

**29**

Sø 27 nov 2016 07:52 # 262

Wow tillykke 26 , fantastisk god. Raging speed ????

**29**

Sø 27 nov 2016 07:52 # 263

Antag disse spørgsmålstegn

**27**

Sø 27 nov 2016 08:58 # 264

Det er fordi du laver emojis, de virker ikke

**25**

Sø 27 nov 2016 13:46 # 265

26 : 

Tillykke med at du nu er tocifret, 

Vægtet mig i dag 99,1 tocifrede her med. 

Føl dig vidunderligt 

Du er det første mål

**26**

Sø 27 nov 2016 14:03 # 266

Dejlige 25 ! Hvad er din målvægt? Min er et sted mellem 70-80, se hvor jeg lander.

**25**

Mandag 28 Nov 2016 08:11 # 267

Ved ikke, hvor min målvægt ligger. 

Kan se, når de føler sig godt til loven. 

Ja, jeg vil ikke være slank som en pind i det mindste. Så måske omkring 70 eller deromkring

**27**

Mandag 28 Nov 2016 08:36 # 268

Her går det langsomt, men langsomt har jeg også ret, antager jeg. 87,0 i dag så minus 7,4 siden operationen (3v). 

Bortset fra langsomt kommer jeg til toppen!

**25**

Mandag 28 Nov 2016 20:34 # 269

7,4 om 3 uger tror jeg det er godt. 

Kroppen skal tage den.

**26**

Mandag 28 Nov 2016 20:54 # 270

Ja, det er rigtig godt! Har faldet 9 kg siden da, og det har været mere end fire uger siden. Tænk det er vigtigt at have travlt med vægttab. Jeg synes, det er utroligt rart at ikke "jage" kilo som du tidligere har gjort på kostvaner ...

**26**

Mandag 28 Nov 2016 21:46 # 271

Nadver!

**26**

Mandag 28 Nov 2016 21:47 # 272

Forsøgte at uploade et billede, men det synes ikke at fungere. Ikke i appen alligevel. Nogle ost og skinke, kirsebærtomater, agurker og sukker ærter blev lavet i aften. Yum!

**25**

Tirsdag 29 Nov 2016 19:45 # 273

Stop med at 

udsætte en person, der kun skal spise flydende Hahaha, de så så godt ud. 

En uge til puree, som jeg næsten ikke kan bringe mig. 

Så joooooo, kom billedet op

**26**

Tirsdag 29 Nov 2016 21:46 # 274

Åh undskyld Mija! Troede ikke på det 
snart! Hold på!

**26**

Tue 1 Dec 2016 07:45 # 275

Det er tidligt end for dem, der har arbejdet, men tænkte på, om du vidste om dumping? Jeg har kendt (hvad jeg synes er dumping) to gange hidtil. Har ikke modtaget hjertebanken, som den ofte beskrives, men er blevet meget kedelig og træt og virkelig. Har følt mig så slemt, at jeg har brug for at få opkast, men selvfølgelig kommer der ingenting, når jeg bliver syg (meget mærkelig følelse forresten!). I begge tilfælde er det kommet en halv time efter maden, og det er utroligt ubehageligt. Første gang det skete, havde jeg spist kogte æg med pyttelite kaviar (måske sukker i kaviaren, det var årsagen?). Anden gang havde jeg spist flager (fitness) med lidt mælk. Der var ikke meget sukker i flagerne, men mælken var sandsynligvis et typisk begyndermåltid, fordi det var lidt som at drikke med maden, det blev skyllet for hurtigt i tarmene ... 

I går spiste børnene pizza og jeg bestilte en kebab salat. Jeg følte mig ikke dårligt om det, men jeg formåede ikke at spise særlig meget, så det blev tre portioner af en. Godt at vide, at du har muligheder for dig selv de få gange, du bestiller hjemme mad. 
Hav en god dag!

**25**

Fre dec 2, 2016, 18:47 # 276

27 og 26 hvor længe gik du ovenpå? 

Disse supper sætte mig op i min hals, jeg er ked af at tænke på dem, så i et par dage tilbage spiste kun mælk, mælk og hjemmelavet smoothie ingen varm mad overhovedet. 

Aldrig mere suppe for mig så længe jeg lever 

Så sjældent træt af det nu. 

Ikke spiser noget vittig i over 3 uger, men jeg ved, jeg vil ikke klage, men ja, de gør det stadig hahaha

**27**

Fre dec 2, 2016 19:14 # 277

Bare en uge faktisk. Jeg ringede diætisten og græd næsten, jeg klarte ikke det. Jeg var okay at starte ren mad, så det var fint, så det var ok

**26**

Lør 3 dec 2016 00:39 # 278

Bare en uge! Instruktionerne fra mit hospital var flow i en uge, blød i tre uger. Jeg følte mig så dårlig for supper af en eller anden grund. Det virkede bare ikke. Jeg levede på skummetmælk og vlcd under mit fly.

**25**

Lør 3 dec 2016 10:41 # 279

AHA !!!! 

Vi flyder i to uger. Og sen puré 3 uger. 

Kalding af diætisterne på mandag til ja virker virkelig ikke.

**27**

Lør 3 dec 2016 11:51 # 280

Nej, det var forfærdeligt at flyde. Jeg levede som 26 på lysfil. Hot supper fik mig til at spionere og kolde supper, da Proviva var så himlen sød. Jeg rev nogle gode parmesaner i Keldas tomatsuppe. Det gjorde under alle omstændigheder suppen bærbar.

**26**

Lør 3 dec 2016 15:10 # 281

Ja, se hos diætisten 25 . Det er nok godt at gå forbi lidt tidligere. I dag har jeg svømmet i en kilometer. Det var helt vidunderligt. Og det var så utroligt dejligt at bruge lang tid bagefter. Jeg hader at træne ud, men jeg elsker at svømme! Så det kan være min form for uddannelse ... Jeg bør også bruge styrken i gymnastiksalen. Er så utrolig svag i armene og ryggen / maven. 

Ønsker jeg kunne være den, der virkelig brydde sig om gymnastiksalen, men jeg kunne aldrig lide det. Synes det er rent skadedyr og smerte at træne.

**27**

Man den 5 dec 2016 19:20 # 282

Hvordan går det for alle? Jeg sænker langsomt og får toppen på alle måder . Jeg forestiller mig, at jeg begynder at føle en forskel i tøjet nu, men jeg ved det ikke. 

Hvor ofte vejer du? Måler du også? Hvor ofte, hvis så?

**26**

Mandag 5 Dec 2016 21:13 # 283

Hej 27 ! 

Langsomt for min del. Ønsker mig hver mandag, men måler mig ikke. I denne uge er jeg gået ned 1,5 kg. Føles som et godt tempo, tror jeg! Hvor meget går du ned en anden uge? 

Absolut mærker på tøjet. Får mange tøj på mig, som jeg ikke har kunnet bære i lang tid. Føles godt! 

Jeg svømmer for en kilometer i aften igen. Føles så let og sjovt i vandet.

**27**

Tis 6 dec 2016 07:46 # 284

Jeg vejer søndagen og havde planlagt at måle en gang om måneden. Her er det 1-1,1 kg om ugen, og jeg ved, det er ret godt, men det føles langsomt nogle gange. Men jeg har ikke kunnet træne af forskellige grunde, så det er nok formentlig fordi det er lidt langsomt. 

Det har været en måned siden operationen, og jeg er meget begejstret for mine nye dimensioner. Der tror jeg det vil være meget synligt nu!

**31**

Tirsdag 6 dec 2016 19:43 # 285

Her flyder vi på! 2 uger af 4 afsluttet. Nu er jeg utroligt træt af supperne !!. Mindre end 6 kilo hidtil. Målet for kirurgi -6,5kg så det er tæt, men de to uger tilbage, så der burde ikke være noget problem at tabe et par kilo til. Plys !!

**27**

Vores 7 dec 2016 08:09 # 286

Det var en masse centimeter, der er forsvundet siden operationen, sjovt at se! 

Ankel: -0.5 cm 
Hvad: cm-1 
Under knæ: 2 cm 
end lap -2 cm 
lår: -4 cm 
Hip: -4 cm 
Talje: cm -3 
Bryst: -8 cm 
Hals -3 cm 
humerale : -1cm Håndteret 
: -1 cm 
- - - - - - - - - - - - I alt -29,5 cm! 

**29**

Ons 7 dec 2016 08:47 # 287

31 : Hej du! Hvornår vil du blive betjent! 

Jeg venter på dig, nok v 4 !!! Så det nærmer sig !!! Få besked i morgen, når det bliver det. Snart suppe til mig med. Fy, ser ikke frem til den smule. 

Det er nok godt, men du vil have mere. 

Hug

**29**

Vores 7 dec 2016 08:48 # 288

Åh, mange cent 27 . Godt arbejde.

**31**

Vores 7 dec 2016 15:43 # 289

Tak! Jeg forsøger at tro, at mere end halvdelen er gået nu. Plys! Den 20. december opererer jeg. Lidt skræmmende, men ser frem til masserne!

**29**

Tue 8 Dec 2016 16:27 # 290

31 - Spændende og lidt skræmmende også. Great når svampen tid er overstået!

**26**

Torsdag 8 dec 2016 17:15 # 291

Jeg har stået stille i vægt siden mandag. Det er meget frustrerende (jeg ved, jeg burde ikke holde fast og veje ...) Ved ikke hvad det er, men jeg har det lige nu, måske er det spøgelse ....

**31**

Tirsdag den 8. december 2016, 17:37 # 292

Hmm .. sidste dage har vægten stået stille, selv jeg var gået op 300gram da jeg vejede i morges! Dette er ikke muligt! o

**26**

Mandag 12 december 2016 08:03 # 293

95 kg i dag. -2,5 kg i denne uge! Nu er jeg kommet halvvejs fra 119,5-70 kg!

**31**

Man 12 dec 2016 18:49 # 294

Tæl hvor hurtigt det gik! ???? En uge tilbage til operation for mig, begynder at blive nervøs!

**29**

Mandag 12 december 2016 20:18 # 295

Great 26 

31 , snaaks dax forstår, at du begynder at blive nervøs. Jeg venter ivrig efter mit opkald hver dag

**34**

Tirsdag 13 dec 2016 13:03 # 296

Hej! 

Nu har jeg læst halvdelen af ​​tråden i det mindste, og jeg er så glad for at jeg fandt det! 

Håber, jeg får dig. Her er de hurtige puckere! 

Jeg var på sundhedscentret efter 6 års fejring den 23. november og en henvisning blev skrevet. I går havde jeg et mødemøde, og IDAG kaldte sygeplejersken og spekulerede på, om jeg kunne komme til en vurdering allerede på fredag, hvilket jeg selvfølgelig takkede for. 

Nu er jeg selvfølgelig supernervøs over vurderingen og spekulerer på, om nogen kan lide at fortælle dig, hvordan det går, og hvad de spørger? 

Jeg er 163 og har mit livs vægt mindst 103 kg, men nu er jeg kun "95 kg". (Har svunget op på de 30 kg sidste 10 år), ligger jeg derfor lige på den BMI tærskel og har ingen sygdomme (endnu), og så bange, at jeg får et nej, ikke sige de forkerte ting. 

Nu er det sådan, du håber du kan læse, du synes at være en god flok lejligheder og super godt !!

**29**

Tirsdag 13 dec 2016 15:36 # 297

Selvfølgelig vil du tjekke. 

Jeg var på min læge i november, pch jeg er sund, men fedt. Har BMI 37 og behøvede ikke at forklare så meget mere, end jeg har mistet hele mit voksne liv og besluttet efter mange års trivsel ligesom dig. 
Håber Få Optimal Gastric Sleeve Uge 4 

Sjov at høre, hvordan det virker for dig.

**34**

Tirsdag 13 dec 2016 16:14 # 298

De sagde i går, at de ikke havde lange ventetider efter at være blevet godkendt her i XXXX, så jeg tror, ​​det bliver januar for mig også. Det ville være sjovt, hvis vi kom rundt på samme tid, fordi jeg også vil lave en ærme nemlig!

**29**

Tirsdag 13 dec 2016 22:30 # 299

Vent venligst ikke. 
Ja, så håber vi, det vil være på samme tid. Både spændende og skræmmende det med kirurgi, jeg er mest fascinerende til såningen .... men det går godt. 
Og 4 v pulver værdi før op ....

**35**

Tirsdag 13 Dec 2016 23:30 # 300

Du, der har lavet GBP eller DS, er så bare MODIGA !! Du vil være meget stolt af dig selv! Jeg er en fan stolt af mig selv! Og jeg har ikke engang foretaget min operation endnu !! Men nu på mandag 19/12 er det endelig dax. For nylig kom jeg til en T-kryds og måtte vælge en vej. Til venstre var det 

"DOOD IN SHORT" Og til højre var det "LEV LÄNGE" Og du behøver næppe at være en raketforsker for det valg !! Jeg er en simpel XXXX, og alligevel valgte jeg den rigtige måde !! Del dine oplevelser og oplevelser med DS. Både Positive og Negative. 

MVH fra XXXX vejer i dag 175 kg, men vejer ikke mere end 125 kg i 8 måneder !! Hvad synes du om det ?? 

KROSS TIL ALLE !! ????????????

**34**

Tue 13 Dec 2016 23:44 # 303

Har du været i fortiden? Jeg har været der to gange til alle e lige så søde at have det sjovt, så jeg er så høj som et hus, så det er bare dejligt at sove, jeg har følt mig sikker! 

Jeg ser pulverværdien mindre end den har gjort så meget. 3 måneder er pladen, da jeg gik til Itrim! Jeg har så hovedpine i de første par dage og er så vred at citroner skulle finde et nyt job :) Men efter fire dage forsvinder sulten, så bliver det bedre.

**35**

Tirsdag 13 dec 2016 23:52 # 304

Beklager, at det blev skrevet tre gange. Kan heller ikke fjerne. Eller måske går det. Men som jeg sagde, er jeg XXXX og ikke raketforsker ???????? 

Pas ALLE, og glem ikke at leve livet hver dag i de 365 dage om året, og hvert fjerde år 366 dage !! ????????

99,9 NU !!

**29**

Vores 14 december 2016 07:06 # 305

Dejlige XXXX !!! Ja, det er vejen at gå og tro på at være den bedste !!!! Held og lykke på din operation!

**29**

Vores 14 december 2016 07:08 # 306

34 

Jeg er slukket, da jeg var yngre, men følte mig ikke skræmmende, når jeg husker. Men nu hvor du er mere opmærksom på risikoen, er jeg lidt bange for bare den smule, men ikke så bange for, at jeg ikke er dristig. Vil bede om beroligende, hvis jeg ikke får det på rutine ...

**25**

Ons 14 dec 2016 12:50 # 307

27 og 26 Nogle tips hvad skal du spise sådan 3 uger efter operationen ?? Hvad så du på? Træt af puré, så de har tygget noget, men tygget let, som tygger ja, indtil der ikke er noget tilbage knapt. Maven har ikke reageret på det faktum, at ja er kommet godt i rutinen med at spise drukket som før det var et fuldtidsjob. Men som sagt, træt af puree har ingen god fantasi om hvad ja kan spise. Vil være en masse æg alligevel. De vejer i dag, så godt ned 15,2 kg sent, og begyndte med min strøm, så de vil være om 5 uger. Ikke mere ondt i mine knæ, når ja, ja, jeg føler mig så glat som en panter på trods af min 95 kg 

**27**

Vores 14 dec 2016 14:26 # 308

Jeg hoppede hurtigt over puree og gik lige for at spise alt andet end tygget ordentligt. Ved "alt" mener jeg godt, god mad. Hvad jeg stadig er forsigtig med er fed mad, stegt / breaded mad, brød og selvfølgelig slik, chips, sodavand osv. 

Men som normalt spiser jeg meget små portioner.

**25**

Vores 14 dec 2016 14:39 # 309

27 : 

De er begyndt en lille smule, men så var de bekymrede for at det kunne være for tidligt. 

Men så fortsætter jeg som ja nu for at introducere mad lidt omhyggeligt til kun meget let tygget mad. 

Tak :) 

Et spørgsmål til, 

hvor længe har du trykket på dine ar i din mave?

**25**

Ons 14 dec 2016 14:40 # 310

Endnu et andet spørgsmål 

Spiser du råbrød endnu?

**27**

Ons 14 dec 2016 14:49 # 311

Jeg tror, ​​du ved klart, om din mave er ubehagelig med den mad, det bliver, så får du lidt tilbage. SSK sagde, at jeg ville være 6 måneder gammel for at gøre arene så hyggelige som muligt. Jeg ved ikke rigtig, om det er rigtigt. Det vil beskytte solen første år alligevel, men måske er det nok til at tape de 1-2 måneder? Mit bånd kan tage et stykke tid, det generer mig ikke. Jeg har testet lidt groft brød og det fungerer godt. Tygge, Tygge, Tygge kun! 

**34**

Fre 16 dec, 2016 11:25 # 312

Jeg blev godkendt! Jisses så nervøs! Jan 16 er tiden

**27**

Fre 16 dec, 2016 11:49 # 313

Tillykke!  

Hvis jeg ved hvad jeg kender i dag, ville jeg ikke have været en dugg nervøs, bare forventningsfuld. Håber du får en lige så let og ukompliceret tur som mig!

**25**

Fre dec 16, 2016 13:24 # 314

Tillykke 

Ja hva oxa- nervøs, gad vide , om de var værd, formodes at X børn vokser op uden deres mor osv osv 

Havde reelle angst på ikke vidste hvis ja gjorde det rigtige valg. 

Da de senere kørte os på et rullende bånd, 6 stk på en dag. 

Hvor godt er du nødt til at måle bagefter. 

Samme dag hvad gjorde du? 

3-4 dage efter operationen, hva næsten som sædvanlig, 

Min mand, på ja stadig tale om, at MSN ikke kan tro, at du havde kirurgi, 

Du var engang god på livet fortsætter som normalt ???? 

Sig de 27 , du har intet at være nervøs over, bare forventer, at fremtiden nu har at byde på

**29**

Fre 16 dec, 2016 16:01 # 315

34 Tillykke, jeg har det fint, indtil videre.

**29**

Fre 16 dec, 2016 16:48 # 316

Jippie jeg har fået tid !!! 26/1 !!!!! Opkaldet var i postkassen i dag !!!! Jeg blev nervøs. Pulversuppe fra 28/12 ....

**34**

Fre 16 dec, 2016 18:00 # 317

Mange tak! Du har det fint !! 

Tillykke med snara79!

**26**

Fre 16 dec, 2016 19:35 # 318

Tillykke med snara og sdbd !!!

**29**

Lør 17 dec 2016 13:41 # 319

34 hvor lang tid vil du drikke pulversuppe før din op?

**29**

Lør 17 dec 2016 13:44 # 320

31 , Dax for dig nu om et par dage !! Har du gode tips om ngn ok pulver suppe ?? Nå, hvis du prøver lidt anderledes, så ved jeg, du bliver træt af dem lige hurtigt. 
Held og lykke med din op !!

**29**

Lør 17 dec 2016 13:45 # 321

Mange tak! En spændende tid fremad!

**34**

Lør 17 dec 2016 15:21 # 322

To uger! Så jeg starter det 2/1. Jeg spurgte om jeg skulle gøre mere for at gå ned så meget som muligt, men jeg fik det ikke, noget der ikke skulle tørres ud. Det var kun dem med BMI <45, der ville køre 4 uger. Jeg er nødt til at gå ned 6% omkring 5,5 kilo før

**31**

Lør 17 dec 2016 15:57 # 323

Tak 29 !! Tirsdag klokken 9 er det tid! ???? Jeg arbejder som nummer to den dag. Bliver meget nervøs 

De eneste supper jeg kan lide er Modifast's Jordbær, Vanille, Kartoffel og Lækre og Svampesuppe. :) Men jeg er kræsen .. 

Hvornår er det tid for dig ??

**29**

Lør 17 dec 2016 16:30 # 324

Godt for dig. Her i XXXX er der 4 v for Alle ... men ligesom situationen. 
Jeg skal gå ned 5kg, så det bliver ret nemt 4v tror jeg. Ja, du lærer at være helt loset, når du har arbejdet siden. 

/ XXXX

**29**

Lør 17 dec 2016 16:32 # 325

Jeg forstår, at du er nervøs, jeg vil være. Men det går godt. 
26 / Lad mig stå op. Tilmelding 12/1. 

Tak for de tips, jeg er ivrig efter de skræmmende supper.

**34**

Lør 17 dec 2016 19:02 # 326

Ja, fem kilo har du 1 uge vlcd! Jeg forstår at fire uger føler sig tunge, på den anden side får du en rigtig start med vægttab!

**25**

Søn 18 dec 2016 08:14 # 327

Jeg flød med Modifast men vi selv fik lov til at spise chokolade og vanille budding og de forskellige pasta varianter og selvfølgelig den ryster. 

Havde de været en suppe, ja, de ville aldrig have gjort det

**27**

Søn 18 dec 2016 09:56 # 328

-10,2 kilo i dag! Når det er sagt, går det ikke hurtigt, men det går :)

**29**

Mandag 19 Dec 2016 14:40 # 329

Åh, har ændret min tid til at være 7. Tilmeldt mig på arbejde. Men det føles stadig ok. 

Hav en god uge!

**31**

Mandag 19 Dec 2016 18:32 # 330

Jahapp, i morgen klokken 9 er det tid! Begynd at blive temmelig nervøs, er mest bange for operationen selv, men det bliver fint .. Hua!

**34**

Mandag 19 Dec 2016 22:34 # 331

Held og lykke! Det vil gå til toppen, du vil se

**34**

Mandag 19 Dec 2016 22:35 # 332

De er meget gode! Tillykke

**31**

Tirsdag 20 dec 2016 18:37 # 333 **+1**

Nu har jeg vågnet op. Alt er virkelig gået godt! Bare lidt dårligt, men det værste er, at du er så tørstig. I morgen bliver det bedre. De er virkelig fantastiske søde her på hospitalet! :)

**29**

Tirsdag 20 dec 2016 21:46 # 334

Rart at høre! Håber du har det godt i morgen! Hvor længe vil du være på et hospital? 

Med venlig hilsen 
29

**31**

Ons 21 dec 2016 07:58 # 335

Jeg ved ikke hvor længe, ​​prøv at få vand herinde og få mig mindst 1,5 liter, før jeg kommer hjem. Efter at have haft en dårlig tid, men ikke så farlig som min værelseskammerat her, lavede en ærme, hun har haft en god nat hele natten, så hun har lider smertelindring hele tiden. Stackarn .. Jeg spurgte sygeplejersken, hvis det er dårligere opsving med ærmet og det er hvad hun sagde. Jeg føler mig meget ked af min værelseskammerat .. Ellers er jeg ret cool, jeg må sige!

**29**

Tors 22 dec 2016 13:34 # 336

Åh ja, det lader til, at Sleeve Up har mere smerte, at forberede sig på det. Hvordan gør man det nu ???? 
Håber du kommer i din væske, så du kommer snart hjem.

**27**

Torsdag 22 december 2016 17:54 # 337

Jeg var ikke særlig såret. Det var lige efter OP opvågningsstuen, når min nål sæt slemt som smertestillende medicin ikke kom ud som det skal, så den første nat. Men så om natten var det ikke værre end jeg var op og gik 3-4 gange omkring afdelingen.

**25**

Fre 23 dec 2016 07:08 # 338

Så har den præcis en måned siden yes gjorde kirurgi og har den måned gået ned 7,4 kg, men flydende er godt nu tabt 17,3kg 

17,3 kg i én måned på to uger, så syg tilfreds. 

Jeg følte også, at de, der lavede ærmet, kunne føle sig værre. 

Vi huh 4, der gjorde GBP på alle lige deroppe på risers 

På højre fire, der gjorde ærme, to af dem, der gjorde det højre ærme var følelse syg og måtte blive længere på grund af dette. Man følte virkelig dårlig, at man havde smerte hele natten. 

Du skal sørge for, at du bevæger dig så hurtigt som du kan og kan, og forsøger at få væsken jo hurtigere bliver du bedre. Ved denne slange i næsen, ja, så snart jeg var for meget til at føle mig dårlig, troede de, at de hjalp. 

Det samme for at blæse røret du får, de hjalp ikke meget, når du følte dig dårlig. Det gjorde en vejrtrækning af rolige lixom. 

29 

Så vi håber du er en af ​​dem, der føler sig bedre og hurtigt på dine ben hurtigt.

**31**

Fre 23 dec 2016 09:13 # 339

Nu har det været 4 dage siden operationen, og det er så svært at få, hvad jeg skal !! Det gør ondt, når det kommer ind i maven, og jeg er slet ikke sulten eller sulten! Hvornår bliver det bedre? Jeg har heller ikke kunnet gå på toa og føler i mine tarmer, at den er fuld af gas, så jeg er syg på grund af det. Hvordan var det for dig andre?

**31**

Fre 23 dec 2016 10:24 # 342

Jeg korrigerer, 3 dage er gået. :) 

Så ved jeg ikke hvordan man kan slippe af med mine dobbelte ting over ..

**25**

Fre 23 dec 2016 11:54 # 343

Jeg gjorde nr. 2 den anden dag. Åh ja, det er svært at få mad til vand. De er stadig i dag, hurtig lysere med vand nu. 

Kan indstille uret til at spise i overensstemmelse med de instruktioner, du har modtaget. 

Hvis du ikke har bedt efter 4 dage, kan du købe en kløver hos Apoteket. De har vores instruktioner. 

Men min gas kom ud dagen 2-3, så de kom ind i maven. 

Men du er blevet kysset alligevel

**26**

Mandag 26 Dec 2016 14:37 # 344

Hej! Håber du har haft en god jul! Jeg har arbejdet hele julen, men nu er jeg endelig fri. Har spist mange godbidder i julen, men kun meget lidt af alle ting. Har ikke målt dårlig eller dumpet på noget. Havde stadig faldet næsten to kilo denne uge. Har nået en milepæl i dag, når mit BMI er nu 29,9 !!! 
Føles vidunderligt! Vil min første opfølgning i en uge ????

**29**

Mandag 26 Dec 2016 14:52 # 345

Tillykke Bradbury! 
Fantastisk, at du er gået ned over julen. Vidunderligt, ja. Jeg er ikke vant til det med det samme.

**36**

Tue 27 Dec 2016 18:28 # 346

Hvad er forskellen mellem gatric bypass og sleeve? hvem vælger hvilken man skal tage eller gøre det selv?

**31**

Tors 29 dec 2016 17:19 # 347

I bypass fjerner du næsten hele maven og forbinder tyndtarmen. I en ærme fjerner du en stor del af maven, så det er kun en smal passage tilbage.

**26**

Sø 1 jan 15:47 # 348

Hej alle! 

Hvordan har det været for dig i weekenderne? Hvordan har du det? Jeg har spist mest men meget lidt af alt. Selv drak nogle vin. Fortsætter langsomt nedad og vejes 91,1 i dag. BMI 29,4.

**27**

Sø 1 jan 18:51 # 349

Hej hej og held og lykke! 

Det har været godt for mig. Jeg har også spist, hvad jeg ville have, men det betyder ikke noget mere. Jeg var på et julebord, som var fantastisk, men det var lidt spild af penge i søen, fordi du næppe kan spise noget overhovedet, haha! 

Jeg er gået ned til 82,4 (12 kg totalt) og har nu et BMI på 29,55. Jeg tror stadig, det bliver skørt langsomt, men er stadig tilfreds, så længe det går rigtigt

**25**

Tue 3 Jan 09:02 # 350

Nå, det har været godt. 

Ligesom dig andre, spist af alt, men meget lidt. 

Faldet ned 19,5 kg sent ja startet med strømmen vejer i dag 91 kg 

Så de vil være meget tilfredse med på lidt under to måneder

**31**

Tue 3 Jan 12:51 # 351

Juleaften var kun få dage efter operationen, så det blev bare en suppe. Nu har det været to uger efter operationen. Libraen har stået stille i et par dage, men antager at det er normalt. -15 kg siden flyvning starter 21. november. 

Tester mig endnu og har endnu ikke dumpet alvorligt. :)

**37**

Tirsdag 3 jan 20:50 # 352

Hej! 

Meget interessant tråd, så jeg hopper ind! ?? 

Jeg sendte en selvpræsentation i mit amt, 12/12. Men tror, ​​at det kan være en meget lang ventetid. Så du, at XXXX blev nævnt her, at det ikke er så lang kø der? Kan jeg sende en henvisning til et andet amt og anmode om operation der, via amtsrådet? Vil det koste mere isfald? Og i XXXX kunne du ikke sende en personlig kommentar? Eller ... 

Tusindvis af spørgsmål ??

**31**

Ons 4 jan 19:50 # 353

Nu har jeg dumpet rigtigt .. onkel! 

Til nogle ost udsnit og efter en halv time, jeg fik så syge mavesmerter og diarré verden .. blev svimmel, koldsved og følte syge. Vær ikke sjov nu, jeg vil være forsigtig.

**38**

Ons 4 jan 20:30 # 354

Hej, jeg bor i XXXX . Har været i sex siden foråret blev indkaldt til et gruppemøde i oktober. Men kunne ikke skyldes mine uddannelseskrav. Nu venter jeg ivrigt at blive kaldt igen. Læs igennem og fundet det andet i XXXX, da det gik hurtigt. Jeg er meget bange, men stadig meget motiveret. Har valgt ikke at gå ud med dette til mere end familie og nogle venner. Hvordan har du gjort det, har du fortalt mange?

**29**

Vores 4 Jan 20:53 # 355

Hej der! 
Jeg bor i XXXX ( XXXX ) og står op uge 7 og venter på opkaldet, så jeg får datoen. Spændende og skræmmende på samme tid. Jeg har valgt ikke at gå ud over, hvad jeg skal gøre mere end til familie og kolleger, for at være gradvis. Jeg var på gruppe i oktober. Jeg har ventet i lang tid, men på grund af Aspire-metoden ønskede jeg at ændre mit sind. Var for lang til at vente der. 
Sendt i min egen præsentation Feb-15.

**38**

Ons 4 jan 21:02 # 356

Så blev vi sandsynligvis opfordret til det samme gruppemøde :) Det har været meget hurtigt sent, da du var der. Kan jeg ikke fortælle hvad der skete med gruppemødet og efter?

**29**

Ons 4 jan 22:05 # 357

Ja efter det er det gået hurtigt. Informationsmødet var 4 timer. God information om metoder og komplikationer. Føler jeg var mere peppered end bange, hvad jeg bekymrer mig om er stoffet ... lidt skræmmende. Men det er det, det kræver. Ser frem til det, men udfasning af pulvertopperne i 4 v ... begynder hurtigt v. 3. 
Efter gruppemødet opfordrede læge besøg 21/11, besluttede jeg mig at rejse mig op. Opført på venteliste. Føltes godt. Lægen var stor. 
Fik optaget 26/1 først, men på grund af arbejdet sat op, tag senere, det vil sige v 7 desværre. Men ikke så lang tid at vente.

**29**

Ons 4 jan 22:06 # 358

38 - Hvad laver du?

**38**

Ons 4 jan 22:22 # 359

Var du på doktors besøg? Jeg læste til XXXX, vi havde øvelse og fik kravet, hvis vi savnede mere end en vis procentdel, så vi måtte tage et sabbatsår. Så tør ikke risikere. Så håber jeg bliver kaldt inden udgangen af ​​april. Før det går jeg til mit andet praktikophold. Hvad arbejder du med? Er med stoffet, der skræmmer mig. Men mødte en, der arbejdede i oktober i XXXX , sagde hun, at stofmisbrugeren havde fortalt hende, at hun kunne være rolig, når de var tilsluttet.

**39**

Torsdag 5 jan 13:47 # 360

Hej! Ny her ... ?? I midten af ​​december sendte jeg en selvbetænkning. Kommer til januar / februar informationsmødet engang. Fik en besked pr. Telefon, da jeg ringede i går og kontrollerede, om meddelelsen kom rigtigt?! ???? (Venter på opkald via mail nu.) ???????? 

Er der nogen her, der modtog en operation via landstinget, med en bmi mellem 30-35? (Lidt bekymret ...) Jeg har brug for hjælp med min fedme, som jeg har brugt i 25 år. Har forsøgt alle træningsmetoder, slankekure osv. - uden langvarige resultater. Jojjo smuttede frem og tilbage i alle disse år. Har nogle arvelige sygdomme i familien, der ikke er omfattet af at være overvægtig, så jeg er meget ivrige efter at hjælpe med vægttab gennem kirurgi. Min sidste udvej ...!

**26**

Torsdag 5 jan 14:36 # 361

Jeg tror ikke, du kan få hjælp fra amtsrådet, hvis du har en BMI over 35. XXXX er rock hårdt på BMI 40. Undtagelsen er BMI over 35, med svære medicinske tilstande (de ikke tager højde for arvelighed) såsom diabetes og højt blodtryk. Har ikke hørt om noget amt, hvor du får operation med lavere BMI end 35, men jeg ved ikke, hvordan det virker hvor som helst.

**39**

Tue Jan 5, 14:44 # 362

Tak for svaret 26 ! 

Så får jeg hjælp på en anden måde, formoder jeg. Via kostråd fra en diætist måske.

**29**

Tue Jan 5, 23:04 # 363

Ja, jeg gik der selv, troede, at skaberen gik, men senere gik jeg. Nu kan jeg forstå, du vil ikke læse om deres uddannelse. Jeg arbejder som jordemor. 
Ja, jeg er blevet kastet en gang før i mine teenagere, men stadig lidt bange, men samtidig ved jeg, at de er gode. I dag har jeg fået min oppetid !! 13/2 opstår, og tilmeldingsfejl 31/1 blev nervøs nu.

**38**

Tue Jan 5, 23:47 # 364

Hvad menes med tilmelding? åh så spændende! ' Jeg er nervøs for muligvis at få et nej.

**29**

Fre 6 jan 11:43 # 365

38 
Du møder anæstetiker, diætist, fysioterapeut, læge forbereder opadrettede.

**29**

Fre 6 jan 11:44 # 366

38 , du har bmi> 35 du vil ikke få et nej.

**27**

Fre 6 jan 14:30 # 367

Nu har jeg haft en måledag igen. Sidste gang det så ud som dette: 

Ankel: -0,5 cm 
Hvad: -1 cm 
Under knæ: -2 cm 
Over knæ: -2 cm 
Lår: -4 cm 
Stubbe: -4 cm 
Talje: -3 cm 
Bryst: -8 cm 
Hals: -3 cm 
Øvre arm: -1cm Håndteret 
: -1 cm 
- - - - - - - - - - - - I alt -29,5 cm! Sådan ser det ud til denne gang (dimensioner i parentes er totalt antal cm minus siden starten). Vinkel: -0,5 cm (-1 cm) Hvad: -1,5 cm (-2,5 cm) Under knæ: -0,5 cm (-2,5 cm ) Over knæ: -0 cm (-2 cm) Lår: -2 cm (-6 cm) Stud: -5 cm (-9 cm) Talje: -4 cm (-7 cm) Bryst: -3 cm (-11 cm) Hals: -1 cm (-4 cm) 


Øvre arm: -1 cm (-2 cm) 
Håndtag: -0,5 cm (-1,5 cm) 
- - - - - - - - - - - - I alt -19 cm (-48,5 cm, næsten en halv meter mindre af mig nu!) Næste måleværdien er ca. en måned dvs. 6/2 

**38**

Fre jan 6, 20:43 # 368

Har meget høj BMI. Var lægen, der skrev henvisningen til mig. Så håber på et ja. Skønt jeg er bange. Men jeg skal gøre det for min skyld, har knæ og hofteproblemer (abort tilbage efter graviditet), som jeg synes er påvirket af fedme. Hvordan går det for dig at begynde at flyde? Hvilken slags har du til hensigt at tage?

**29**

Fre 6 jan 23:23 # 369

Åh så godt klaret !!!! Kanonbra jue, masse cm. Jeg glæder mig til det.

**29**

Fre 6 jan 23:24 # 370

Nej jeg er ikke begyndt endnu, jeg har forladt den næste uge med almindelig mad, da kosten starter ... Jeg vil tage lidt anderledes som jeg ved, jeg hader dem ... lidt modofast, nutrilette, allvo .. se hvad der er mindst forfærdeligt.

**40**

Sø 8 jan 22:12 # 371

Anbefaler alle at læse XXXX blog om dette emne, fordi hun har opereret to gange. 
XXXXX

**26**

Man 9 jan 07:53 # 372

Vejer i dag Kom under den 90. linje! Vejet 89,5 i dag. BMI 28,9! 
Hej alle kæmper for det! Det er det værd!

**29**

Tue Jan 10, 17:47 # 373

Tillykke Tillykke! Godt arbejde !!! Ønsker ikke at være der nu !!! Hvordan har du det med mad og så nu? Er der noget du ikke kan spise, eller hvordan spiser du bagefter?

**31**

Tirs. Jan 10, 21:04 # 374

Ni gbp-opade. Hvad er dit gennemsnitlige daglige kalorieindtag? Nu har det været en uge siden bølgen flyttede nedad. Jeg har praktiseret, spiste mellem 700-900kcal om dagen, men intet sker overhovedet. Jeg ved, at du kan blive stillestående, men allerede så tidligt? o

**26**

Tue Jan 10, 22:07 # 375

Jeg synes faktisk at kunne spise alt. Men ikke meget af alt. Har spist alle slags mad og også en lille smule chips og slik. Jeg har følt mig dårligt undertiden. Men så har det været efter regelmæssig mad. Jeg tror, ​​det skete, da jeg spiste for hurtigt eller for meget. Er stadig fattige på at spise langsomt ... bedt om på to-måneders opfølgning, hvis der var noget galt, fordi jeg ikke kunne spise det hele, men de sagde, at jeg skulle være taknemmelig for det, og det er, hvordan de håber, at det er.

**38**

Ons 11 jan 13:56 # 376

Hej, hvordan har du det, fordi du er begyndt at flyde? Ville bare bede om denne gruppe af info, er det bare at gå der og lytte? eller går du i da?

**29**

Ons 11 jan 20:15 # 377

Hej der! Jeg starter med strømmen mandag 16/1. Intet møde er kun info i 4 timer, så stort set info, men godt. Hvornår går du til mødet?

**29**

Ons 11 jan 20:16 # 378

Så rart og ja det er bare og nyd! Godt at høre.

**38**

Ons 11 jan 22:29 # 379

Ja bliver hårdt med 4 timer, vil tage mærke til mig. 24/1. Fik brevet i dag. Var meget nervøs! kort tid efter. :) nærmer sig operationen med :)

**41**

Tors 12 jan 20:46 # 380

Hej der! 

Går ind her. Deltag information om XXXX den 16/2. Føle, at alt gik godt. Har du været overvægtig i det meste af mit liv, men altid fortalt mig, at jeg kan gå ned selv? Men så har jeg haft problemer med min hofte og resten af ​​mit ben (slidgigt, betændelse og ischias), og lægen på den ortopædiske krop troede, at jeg havde brug for at tabe sig. Da jeg ikke kan udøve ordentligt på grund af smerte, vil det være en smule 22. Så nu vil jeg virkelig have det her, så jeg får et liv igen, men nu er jeg nervøs for at jeg får et nej. Har et BMI på over 37. Hvad synes du om mine chancer?

**42**

Tors 12 jan 23:39 # 381

Hej. At blive indkaldt i februar på informationsmødet, BMI 35.4 og lider af dercum o backproblemer. Enhver, der drives eller går på et møde, er lidt nervøs.

**42**

Tors 12 jan 23:40 # 382

I XXXX ???

**29**

Fre jan 13 07:03 # 383

Hej der! 
Velkommen! 
Jeg tror, ​​at de har BMI-grænse 35, så dine chancer for at komme op er nok gode, jeg tror.

**42**

Fre jan 13 10:55 # 384

Håber, fordi jeg har gjort alt fra diætister til ofrene for tab af læger. Dercumen bremser vægttabet. ????

**25**

Sø 15 jan 21:14 # 385

31 

Ved ikke, hvad min kalorieindtag, 

men ja efter at have spist tidsplan for at have 

middag 2 gange om dagen 

100g kød / protein, 

30 g kulhydrat 

På 20g grøntsager 

2 snacks om dagen 

en sprød bund smør på æggene 

så grød eller fil med hindbær på blåbær 

Bliver 5-6 Målet 

rundt om dagen Stadig er det altid vigtigt at holde stille intet end overhovedet.

**25**

Tue 17 Jan 12:06 # 386

Nogen som skal opereres og måske er interesseret i at købe denne bog af mig? 

Efter gastrisk bypass operation 

udviklings- og opskrifter for første gang 

af: Marie Odin, 

jeg troede det var meget interessant samt været nyttig, men den tid til at vandre yderligere 

Nypris 300kr

**29**

Ons 18 jan 18:22 # 387

Tip om smag på supper .. de er for triste. Så gå videre på mandag, dag 3. Føles det ok, lidt i kraniet o men men ellers okay.

**29**

Sø 22 jan 09:17 # 388

Hej til dig. Nu har jeg boet på flow 1 v snart o det virker bedre end forventet. Helgen er gået godt, men lidt kedelig, men jeg har det godt at lave det. Men tænker det er sidste gang jeg gør dette, og jeg overlever det her. Har gået et par kilo, men vil give mig i morgen 1 gange / v for ikke at veje hver dag. Synes at have gået ned iaf ???? Hav en god uge!

**27**

Man 23 jan 08:28 # 389

Nu er jeg under 80 kilo, 79.8 mere specifikt. "Just" 20 kilo venstre joke på hovedet, jeg har sandsynligvis ingen fast målvægt. Jeg håber, jeg føler mig "hjemme" tidligere, og jeg tror jeg vil gøre det også.   

**29**

Man den 23 januar kl 16:52 # 390

Godt! Er det langsomt eller hvordan har du det?

**26**

Man jan 23, 17:36 # 391

87,8 i dag. -31,7 kg i alt. 
Det er så svært at forstå, at jeg har brugt mere end 30 kilo mere end tre måneder siden. Jeg forstår det ikke. Når jeg løfter min ældste, der vejer omkring 23, er det så tungt. Hvordan skyndte jeg mig?!?

**26**

Man den 23 januar kl . 17:37 # 392

29 : Jeg ved ikke, hvordan det er for dig andre, men jeg gennemsnitligt 0,8 kg om ugen.

**27**

Man den 23 januar 19:20 # 393

Ja, det handler om det samme her, 0,8-1,1 kg om ugen. Det har været 11 uger siden, og jeg er gået ned 14,6 kilo (den første uge efter åbningen meget mere end ugerne efter). Jeg var aldrig nødt til at flyde, så mit fald begyndte på dagen af ​​dagen. 

**31**

Tirsdag 24 jan 17:45 # 394

For mig har det nu været en måned siden operationen, og jeg har nu faldet 20 kg i alt. 8 kg under strømmen og resten af ​​denne sidste måned. Bukserne begynder at rive! Mærkelig følelse men så rart!

**29**

Tue Jan 24, 18:41 # 395

Jeg er glad for at høre at du går ned godt alle sammen !!! Godt arbejde. Det er svært nok at forstå det, når du har været fedt i lang tid, men en vidunderlig følelse. 
Flyver du 1-2 v bagefter? Hvornår begyndte du at introducere puré mad og fast?

**43**

Ons 25 jan 15:59 # 396

Hej alle kæmper. Jeg venter i øjeblikket på min driftstid efter at være blevet godkendt! Forhåbentlig bliver det marts / april! Vejer 106 kg 1:63 lang. Jeg skal lave en gastrisk ærme. Som alle her har jeg prøvet ALLE, så det føles som min sidste chance. Svimmelhed i hofter, knæ, ankler. Har også hjerte-kar-sygdomme i familien. Tidligere var det mere for mit selvværd. Jeg kæmpede med min vægt, men i øjeblikket er det mest for sundhed. Har en frygt for fejl, fordi du har gjort så mange gange, men måske en fælles følelse? Netop dette med sugningen !! Har nogen haft samme frygt bare med suge før og har gode tips til at give op?  Jeg vil have det så meget, og jeg vil gøre det, men tag mig ikke af med disse tanker.

**44**

Ons 25 jan 21:52 # 397

Vil gøre min op 21/2! Men kan ikke beslutte, ærmer el bypass ??? 
Hvordan syntes du? Hvis du havde mulighed for at vælge.

**25**

Ons 25 jan 22:59 # 398

Ja, valgte ved pass fordi det har været længere og mere forskning mv er tilgængelig på denne teknik. 

De er afgørende for mig

**29**

Tirsdag 26 Jan 16:03 # 399

CJJ skrev 2017-01-25 21:52:13 følgende:

Vil gøre min op 21/2! Men kan ikke beslutte, ærmer el bypass ??? 
Hvordan syntes du? Hvis du havde mulighed for at vælge.

Jeg valgte Sleeve for at undgå bypassen, og det føltes lidt mildere med bivirkningerne. Men havde ikke desto mindre gjort forbi, hvis lægen anbefalede det. Jg havde også mulighed for at vælge, da ingen risikofaktorer relateret til BMI eller diabetes for at få slæden. Vanskeligt valg men være på farten for lægen.

**45**

Tue Jan 26, 23:05 # 400

Hej. 

Kan jeg banke her og kigge lidt på døren? 

Jeg er 31 år og har været overvægtig igennem hele mit liv. Jeg begyndte at gå op da jeg var 6 år gammel. I det mindste har jeg vejet lidt over 80 kg til min 1,67 cm. Nu vejer jeg omkring 110, jeg tror. Jeg har forsøgt ALLE. Men faldt aldrig mere end maksimalt 10 kg. Jeg har problemer med at gå ned. Jeg ved, at nogle siger, at du gør forkert og spiser for meget, men for mig er det faktisk sådan. Jeg forsøgte Cambridge, sæber og shakes. Ifølge deres info skal du tabe mindst 8 kg om 2 uger. Jeg spiste kun supper (500 kalorier om dagen) og gik 5 km hver dag. Jeg gik ned 6 kg om 3 uger ... 

Derefter har jeg givet op håb. 

Jeg er begyndt at få ondt i mine knæ og kan ikke gøre noget sådan. Har også en konstant smerte i mine skuldre. Fordi jeg er stillesiddende. Jeg er 3 bedstemor, men kan ikke være ude med mine børn om vinteren, fordi der ikke er noget overtøj. Ja, du ved ... 

Jeg har tænkt på kirurgi i lang tid. Men det er lidt skræmmende. Jeg er mest bange for, at jeg vil gå igen efter nogle få år igen. Men jeg tænker på at kontakte det sundhedscenter, hvor jeg bor. Er det den rigtige vej at gå? 

Tilgiv for lang indlæg, men jeg har brug for dig. Dette er så følsomt for at tale med folk, der har normal vægt.

**29**

Fre 27 jan 19:30 # 401

Velkommen! 
Ja, gå gennem dit sundhedscenter eller skriv en personlig anmeldelse (jeg gjorde). Havde også tænkt på mange år og endelig omkring 2 uger vil jeg være op! 
En mulighed der er god til at få et godt liv i fremtiden.

**45**

Sø 29 jan 00:02 # 402

Åh held og lykke!

**46**

Sø 29 jan 00:18 # 403

**45**: Jeg har tænkt på det i flere år, havde allerede en gbp til september, 2015 men slog ud og tør ikke. Jeg kender flere mennesker, der har gjort det, og det har gået rigtig godt for alle, men jeg får mørke tanker og tøven. Nu har jeg tilbagekaldt og ringet til min sundhedscenter for at bestille en læge aftale for at sende en henvisning til gbp-enheden. Føler irriterende, og jeg håber virkelig, jeg vil ikke komme ud denne gang. Nu er jeg over 30, så det er begyndt at føle knæ og fører til, at fedme har sat sine karakterer, selvom jeg vejer så meget siden jeg var 16 år gammel. Held og lykke og peberpeber, at alt går godt: D

**47**

Sø 29 jan 00:42 # 404

Fast 6 kilo om 3 uger er meget! Det er 2 kilo om ugen! Sådan fungerede det for dig! Deres mindst 8 i 2 uger kan ikke stole på, det afhænger helt af startvægten, og alle er vanskelige at tabe sig. Så jeg forstår ikke hvorfor du gav op og ikke troede det fungerede.

**27**

Sø 29 jan 13:02 # 405

Hvornår varer vægttabet faktisk? Nogen der har været i lang tid kan svare, da alt begyndte at stagnere. Var det når du var nede på din målvægt (hvis det var realistisk) eller tidligere? Senere? 

Jeg har beregnet, at hvis jeg går ned stadig 0,8 kilo om ugen, vil jeg være "klar" til midsommere om. Men hvad sker der, hvis du fortsætter med at gå ned? Jeg vil heller ikke være for snæver. Jeg har ekstremt tynde knogler og ser syg, når jeg vejer for lidt. Enhver, der ved? Jeg har "kun" 9 kilo tilbage til normal vægt nu, det føles dejligt!   

**29**

Sø 29 jan 17:38 # 406

Har nogen en vægt blog? Vi vil gerne læse!

**45**

Tirsdag 31 jan 17:15 # 407

Der. I dag har jeg været på sundhedscentret og modtaget en henvisning sendt til operation. Hvad en ting! Fortæller også nærmeste familie og alle er så støttende. Pyha. 

Jeg vejer 114 kg og er 1,67 høj. Har et BMI på 40,9. I Sundsvall (hvor jeg skal arbejde) skal du have mindst 40.

**27**

Man 6 feb 09:25 # 408

Lørdage igen! 
. 
**6/12**
Talje: -0,5 cm 
Hvad: -1 cm 
Under knæ: -2 cm 
Over knæ: -2 cm 
Lår: -4 cm 
Stub: -4 cm 
Talje: -3 cm 
Bryst: -8 cm 
Hals: - 3 cm 
Øvre arm: -1 cm 
Håndteret: -1 cm 
- - - - - - - - - - - - I alt -29,5 cm! 

. 
**6/1**
Øre: -0,5 cm (-1 cm) 
Hvad: -1,5 cm (-2,5 cm) 
Under knæ: -0,5 cm (-2,5 cm) 
Over knæ: -0 cm (-2 cm) 
Lår: - 2 cm (-6 cm) 
Støv: -5 cm (-9 cm) 
Talje: -4 cm (-7 cm) 
Bryst: -3 cm (-11 cm) 
Hals: -1 cm (-4 cm) 
Øvre arm: - 1 cm (-2 cm) 
Håndtag: -0,5 cm (-1,5 cm) 
- - - - - - - - - - - - I alt -19 cm (-48,5 cm, næsten en halv meter mindre af mig nu!) 

. 
**6/2**
Ankel: -0,5 cm (-1-5 cm) 
Hvad: -0,5 cm (-3 cm) 
Under knæ: -1 cm (-3,5 cm) 
Over knæ: -1,5 cm (-3,5 cm) 
Lår : -3 cm (-9 cm) 
Sting: -2 cm (-11 cm) 
Talje: -4 cm (-11 cm) 
Bryst: -1 cm (-12 cm) 
Hals: -1 cm (-5 cm) 
Øverste arm : -1 cm (-3 cm) 
Håndtag: -0,5 cm (-2 cm) 
- - - - - - - - - - - - I alt -16 cm (-64,5 cm, **lidt over en**halv meter mindre af mig nu!) 

**29**

Man 6 feb 19:09 # 409

Åh stor tillykke !!! 
Jeg kan forestille mig smukt smukt. 
Det kommer hurtigt ned. 

Jeg er på champignon uge 4, gået ned 8 kg !! Driften foregår mandag 13/2 !!! Det bliver nervøst nu, men ser stadig frem til det.

**Dementoria**

Man 6 feb 19:27 # 410

Ja, jeg ved ikke, om det går så *hurtigt,*men det er okay, og så er alt fint! 

Din operation vil gå godt, snart er du på den anden side!

**48**

Ons 8 feb 17:59 # 411

Hej. Jeg har været på VC **25. januar**og modtaget en henvisning sendt til XXXX i XXXX . Jeg hedder på gruppemødet den **23. februar**og vil møde en læge den **13. marts**.

**Mit spørgsmål**er, om der er nogen her, der ved eller hørte nogen **bliver nægtet en operation?**Jeg har et BMI på omkring 45, så meget højt. Min læge på sundhedscentret ønskede ikke at sende henvisningen først, da jeg var 27 år gammel og ifølge dem lidt for ung til at gøre en sådan operation. Påmindelsen blev imidlertid sendt, da lægen troede, jeg testede alt andet.

Jeg er lige så bekymret for ikke at få denne mulighed. Jeg er så ked af det nu, jeg har mentalt bestemt, og det føles som om der ville være en sådan tilbageslag, hvis jeg fik et nej.

**26**

Torsdag 9 feb 12:14 # 412

Hej! 

Jeg er meget svært at tro på, at du nægtes operation med et sådant højt BMI, forudsat at du opfylder kriterierne. Du er bestemt slet ikke for ung. Der er nu gennemført en undersøgelse i Sverige, hvor teenagere har haft overvægtige fedme og ikke er gået ned på trods af andre bestræbelser. Med meget gode resultater også. 
www.sydsvenskan.se/2017-01-06/goda-resultat-nar-unga-fetmaopereras 

Held og lykke!

**26**

Mandag 13 feb 20:09 # 413

83,8 kg i dag. Det er så svært at forstå, hvor tung jeg har været ... det er lige nu! Bare et par måneder siden! Hvordan har du det med andre? Hvordan har du det? Hvordan går vægttab?

**45**

Tue Feb 14 15:58 # 414

Helt fantastisk 26 ! Vil være så mærket. Næsten svært at forstå, at det er muligt. Hvordan er livet nu? Hvad ser en almindelig dag ud i maden?

**29**

Vores 15 februar 09:16 # 415

Hej alle sammen! 
Nu er jeg op: annonce (ærme)! Alt gik godt, kranier går hjem fra hospitalet i dag, og det føles godt! 
Føles ret godt nu, men har været et par hårde dage. 
Hug

**26**

Vores 15 feb 19:48 # 416

Jeg har det rigtig godt. Jeg tror jeg spiser noget som før, men meget mindre ad gangen. Jeg har stadig svært ved at spise langsomt så ind i landet. Jeg føler mig meget dårlig, fordi jeg spiser for hurtigt, og det er som strømpe i halsen. Så mange ting, der pludselig er lette, gå uden at svede, læg sko og sokker og mange andre daglige ting. 
Jeg fryser meget og mister MASSA hår. Dette er ulemperne. 
Dejlige Snara! Håber du har det fint!

**25**

Fre 17 feb 21:05 # 417

26 

Jeg taber noget sygeligt hår. 

Tab 3 kvartaler nu, forfærdelig heldig, jeg havde rigtigt tykt hår til ellers ja, jeg ville ikke have haft noget tilbage. 

Hvor længe vil dette vare? 

Har købt Silicea nu, så jeg må muligvis holde noget halm. 

Begynd at blive lidt bekymret. 

Vejer nu 83,9, så det går fremad. 

Min startvægt før strømmen var 110,5 kg Efter flow 100,6 kg 

og 23. februar er det præcis 3 måneder efter op, 

reduceret 20 cm rundt om taljen 

og er mere krydret end noget, det bedste jeg gjorde. 

Kan nu hoppe, skyde danserunde med børnene,

**49**

Tirsdag 23 februar, 22:33 # 418

Hej! Jeg var på gruppens informationsmøde 26/1 og derefter oplyste lægen, at alle ville få opkald til lægehøring inden for 2 måneder. Uheldig som det er, føles det som om dagen går langsomt. Nu til mit spørgsmål til dig. Tror du, at jeg skal ringe og tjekke placeringen i receptionen, så de ikke har glemt mig? Har læst om flere ting, der er blevet "glemt". Men du vil ikke være menig som ... det har kun været en måned hidtil. Jeg vil gerne have operationen færdig før sommeren, når jeg begynder at arbejde i efteråret efter forældreorlov ... ville være nemmere at gøre det nu, når jeg er hjemme.

**29**

Lør 25 Feb 13:50 # 419

Du kan tydeligvis ringe og spørge! Det ville jeg have gjort. Held og lykke ????

**29**

Lør 25 Feb 13:52 # 420

Dag 14 efterhånden begynder jeg at føle mig som før, føler mig glad og glad. Hvad jeg laver er puree og tygge maden omhyggeligt, dejligt at spise og tygge igen! Ved at tage det i små trin i overensstemmelse med den mad, jeg modtog fra diætetikeren. Stingene vil blive fjernet i morgen, og jeg længer efter det, så du kan flytte igen.

**45**

Sø 26 feb 21:31 # 421

Hej. 

Jeg skal bare høre, hvad du synes om en ting. Måske et dumt spørgsmål .... 

Men jeg har så dårlig smerte i mine knæ, fødder og hofter. Hver dag. Knæene er de værste. Har haft smerter i mine knæ fra tid til anden i 3 år. Men aldrig så slemt som jeg har nu. Tror du, du kan få operationen tidligere, hvis du ringer og fortæller? Som det ser ud nu, vil jeg ikke blive betjent til efter sommeren. Det føles så længe at føle sig så slemt ... 

Tror du, at jeg skal ringe og skubbe processen lidt? Eller tror du det er payless. Der kan være mange, der hele tiden er ondt.

**29**

Tirsdag 28 feb 14:41 # 422

Du kan altid ringe til og høre dem! Forstå det føles lang tid at vente til efter sommeren. Håber !!!

**38**

Tirsdag 28 feb 19:17 # 423

Hvordan har du det med påmindelsen? Hvilket amt tilhører du? Jeg har været på et gruppemøde i XXXX for os, de sagde tidligst efter sommeren på grund af manglende tid og personale. Jeg føler utålmodig :(

**45**

Tirsdag 28 feb 21:40 # 424

Samme her. Føles som evigheden ...

**45**

Tirsdag 28 feb 21:41 # 425

Tak. Jeg tror jeg vil gøre det. Kan ikke såre som ... 

Hvordan har du det med alt? Begynder du at snyde på dig?

**29**

Ons 1 marts 16:38 # 426

Når du altid kan spørge! Håber! 

Nå, jeg synes, det går godt, det føles godt og ligner før om. Lidt meck med mad (lidt mad) når jeg spiser for hurtigt. Intet jeg kan lære - spis langsomt. Jeg synes, det er lidt svært at vide hvad man skal spise og hvad jeg kan spise. Kør diætprogrammet, jeg har, men lad dig blive træt, når det er så ensidigt. Spiser puree eller godt tygget mad. 

Ngn, der har en bog til at sælge om mad / recept osv efter gastrisk ærmet (eller endda efter bypass)!?

**25**

Lør 4 Mar 18:33 # 427

Ja, man må sælge af Marie Odin 

Efter en gastric city pass operation kaldes det. 

Tjek det ud online for at returnere, hvis du er interesseret. 

Du kan købe den til 150kr, halv pris :) 

Med venlig hilsen Maria

**49**

Tirsdag 7 Mar 20:17 # 428

Hej til dig! Jeg har endelig modtaget et opkald til en læge besøg så 20/3 er tiden. Nu er jeg så nervøs. Er bange for at blive nægtet. Enhver, der er blevet nægtet eller kender nogen, der er blevet? Af hvilken grund er det? Hvor længe har det taget for dig som drives fra lægebesøg på ovenpå. dag?

**26**

Tirs 14 Mar 14:04 # 429

Hej! 

Håber du har det godt! 
Jeg har haft et par hektiske uger og har ikke engang afvejet et stykke tid. Men i dag var det tid. Under 80 !!! 
Vejet ind på 79,1! Har således tabt over 40 kg og er normal næse med et BMI på 25,5. Ved at du ikke skal snige sig ind på tallene, men se alligevel helt på at tro !!! 

Håber, det går godt for jer alle derude og for dig, der tøver: Det er den afsondrede bedste jeg har gjort, jeg har mit liv tilbage! 

Hug

**29**

Tirs 14 Mar 16:08 # 430

Hej, hvilken tur har du lavet! 

Jeg har reduceret 15,2 kg (siden flyve start 16/1, op 13/2) så jeg er på vej ned, men jeg er utålmodig, og jeg er lidt træt, men jeg er så træt. Efter min vægttab plan (som jeg bragte med mig hjem til hospitalet) som jeg vil. 
Føles godt, men nogle gange kan du ikke spise noget mad (ved ikke hvad eller hvorfor) det bliver bare stop. Måske er jeg, at jeg spiser for hurtigt nogle gange, hvis jeg er sulten. 
Start med at arbejde igen på mandag, og det bliver sjovt! Klar til det sædvanlige liv igen.

**26**

Tirs 14 Mar 18:01 # 431

Dejligt! Godt arbejde! 
Så det er også for mig, det tager nogle gange en kryds strejke. Ofte når jeg spiser for hurtigt som jeg stadig gør desværre. Kan spise mest af tiden, men det ændrer sig meget fra dag til dag, hvad maverne kan lide. Nogle gange er jeg svært at føle mig sulten over noget og kan tvinge mig til at spise. 

Ønsker ikke kager, is og chokolade mere, men føler lidt forlangt efter søde slik og popcorn. Og jeg kan få meget trang til sprøde grøntsager, som salat, agurker, sukkerterter og radiser. Mærkeligt!

**50**

Fre 17 mar 22:18 # 432

Hej, jeg har et spørgsmål, som jeg håber du kan være i stand til at hjælpe mig med. Jeg har modtaget en henvisning for at møde en specialistlæge ( XXXX ) og tale om en operation. Jeg mindede min praktiserende læge, jeg har talt med kirurgens modtagelse, sendt i mere information, og de sagde i receptionen, at det tager omkring 2 måneder, før jeg får tid til et møde med lægen. Du kan leve med det. Men flere i denne tråd synes at være blevet indkaldt til et informationsmøde om kirurgi, inden de møder specialistlægen? Jeg har ikke været tilbudt nogen oplysninger overhovedet? Er det bare nogle landsting, der har dette, eller? Jeg begyndte at forsøge at få en henvisning i januar og fik en i februar, jeg tror alt tager så lang tid! Vil du gerne modtage hjælp før sommeren?

**38**

Lør 18 Mar 21:20 # 433

I XXXX bliver du kaldt et gruppemøde. Jeg var i januar og fortalte os, at vi ikke bliver kaldt en læge efter sommeren på grund af mangel på tid. Men tag et par måneder før du bliver kaldt til gruppeinfo.

**50**

Sø 19 mar 21:18 # 434

Tak for svaret, ja, bare vent og se. Men så mærkeligt, at kirurgens sygeplejerske ikke sagde noget, da jeg ringede ind for at forlade detaljer, hvilke uger jeg ikke kunne møde lægen på grund af arbejde andetsteds. Hun sagde lige, at de indspillede og ville ringe til lægen på et tidspunkt, da jeg rapporterede mig til rådighed. Ingen oplysninger om noget om noget møde. Men vent bare og se hvad der sker.

**38**

Sø 19 mar 23:04 # 435

okay, men du må måske gå en anden vej. :)

**29**

Man 20 mar 22:16 # 436

Ja i XXXX er det gruppemøde og dvaletilstand. (Samme for mig var det). Altid meget mere, end du vil have før sommeren, men så prioriteres andre. Du vil bare gøre dig klar. Optaget med al ventetiden. 

38 , ved du, at din op bliver efter sommeren? 

Kram til alle

**38**

Tirsdag 21 Mar 22:09 # 437

Ingen anelse. De sagde, at vi ville have en læge dag derefter efter sommeren. Så vil vi se, om du er godkendt. De sagde lige, at der er mangel på tid nu, og vi bliver ikke kaldt i efteråret. Hvilket passer mig godt. Skal gå ned, før jeg endda skal gendanne hvis for højt BMI. Så kæmper med det nu. Det er hårdt. Hvordan har du det?

**50**

Tors 23 mar 11:16 # 438

Forvent ikke at vente til efter sommeren. Efter megen snak og spore alle kirurgiske klinikker i XXXX Region (som XXXX Amtsråd har en aftale med) fandt ud af, at ingen kan arrangere en tid inden for 90 dage .. jeg allerede har sat i kø i 2 måneder, og nu har jeg bedt kirurgen modtagelse i K-port for at sende min henvisning henvender sig til XXXX i stedet. Der er også en rigtig lang kø. Men jeg kommer til kø der i stedet. Der er i det mindste en mulighed for at få en pleje før sommeren, hvis jeg er heldig.

**29**

Tirsdag 23 Mar 19:40 # 439

Hold dine tommelfingre for dig! 

Tak for mig! 1 1/2 måned siden op. Spis mest nu og tabe, hvilket er fantastisk! Har reduceret 16-17 kg. 

Hug

**26**

Man 3 Apr 14:49 # 440

Hvordan har du det hele? 
Jeg nåede en ny milepæl i dag! 

Føles fantastisk og vidunderligt! (Selvom jeg ved, BMI er et meget stumt værktøj). Har også startet træning, som jeg aldrig har gjort før. Føler det vigtigt at bygge op, ikke bare tabe.

**45**

Man 3 Apr 21:39 # 441

Tillykke! Hvad stort.

**26**

Tirs 4 april 00:01 # 442

Tak 45 ! Ja, det føles så dejligt. Det er bedst at føle sig godt tilpas ... men det er skræmmende, hvor hurtigt du glemmer. Jeg kan ikke forestille mig, at jeg vejer 40 + kilo mere end jeg gør i dag. Det er uforståeligt ... men jeg kan huske, hvordan jeg var forpustet ved den mindste anstrengelse, hvor svært det var at bare sætte på sokker og komme op fra sofaen ... gyldige nok til at huske alt dette. Det, jeg er mest bange for, er at jeg bliver forvist eller hvad du kan kalde det. Alt har været så utroligt nemt hidtil, så jeg er lidt bange ... måske er jeg bare vellykket nu i starten og om to år er jeg så stor igen? Så forfærdeligt

**29**

Tue 4 Apr 19:35 # 444

Fantastisk 26 ! 
Nej, jeg tror ikke, du gør det! 

Elsker at se dig ned! Har snart nået -20 kg og det er så smukt! 

Føles godt for det meste! 

Hold tommelfingeren for dine andre at stå op og det bliver ikke for lang tid.

**45**

Ons 5 april 09:35 # 445

Er endelig blevet indkaldt til et møde om 2 uger. Føles så spændende! Nu begynder det endelig at bevæge sig. 

Forstår din følelse 26 , det er min største frygt også. Men jeg har aldrig været tynd, og jeg håber, at følelsen af ​​den overgår viljen til at spise ubrugeligt igen.

**51**

Man 10 Apr 09:07 # 446

Jeg skal lave en gastrisk ballon i XXXX i næste uge. er der nogen der har erfaring med det ??

**52**

Sø 16 apr 11:05 # 447

45 , hvornår blev du kaldt et vægtmøde, ventede det længe o hvad sker der efter det? Går du op tid planlagt med det samme eller vil det være en ny ventetid? Er det den samme planlægning for ærme og gbp?

**27**

Sø 16 apr 11:28 # 448

Jeg er også nede på normal vægt. Jeg troede det var meget langsomt i et stykke tid, men så gik det lidt tilbage. Jeg er faldet over en meter i rækkevidde (regnet på hele kroppen). Jeg er gået fra BMI 33.8 til BMI 24.8 og vil fortsætte med BMI 21,1, dvs. 10 kg til. Så er jeg glad! 

Du, der har arbejdet for et par måneder siden, skal du returnere besøg / møder? Hvornår i så fald?

**45**

Man 17 Apr 08:10 # 449

Jeg synes, det er lidt forskelligt fra landsting til landsting. Hvor jeg skal arbejde, skal du gå på et vægtmøde, og så skal du vente et stykke tid, før du møder en kirurg. 

Jeg var på sundhedscentret og modtog en henvisning sendt i slutningen af ​​januar og blev indkaldt til et møde i ugen omkring tre uger siden. Så det virker ikke så hurtigt.

**52**

Man 17 Apr 11:54 # 450

Åh, hvad venter .. Går du op tid, når kirurgen elr vil vente på dette efterår? Føler, at plejegarantien ikke holdes ved vægttab.

**45**

Man 17 Apr 13:52 # 451

Du kan ikke få kirurgens tid, men er sat på venteliste. 

Jeg tror, ​​jeg bliver nødt til at vente på sommeren, der skal drives. Forfærdeligt at vente så længe. Jeg føler mig så slemt i mine fødder, knæ og hofter, så jeg går hurtigt sammen.

**27**

Man 17 Apr 17:22 # 452

En variant er at betale for operationen selv. Jeg gjorde og fik så hurtig tid, at jeg bad om en senere.

**52**

Man 17 Apr 20:11 # 453

Tak for dine svar 45 .. håber du får en hurtig tid o slippe af med din smerte .. forstår dig! Følg dig med spænding, da jeg ikke er 100% sikker på, om dette er min vej at gå, selv om jeg har forstået, at ærmet ser ud til at være et større vægttab. 

Dejligt, at du fik sådan hurtig tid 27 .. vi er nok mange, der ønskede, at vi havde den samme mulighed .. ikke mindst fordi aflæsningerne i dagens gange ????

**45**

Man 17 Apr 20:18 # 454

Du er velkommen til at følge mig " 52 ". Jeg er 100% sikker og ser frem til mit nye liv. Spørg mere, hvis du vil, eller hvis du vil tænke på nogen. 

Jeg ville ønske, at jeg selv havde mulighed for at betale for en operation, men at pengene ikke er tilgængelige. Tænk også på, at jeg må betale for en abdominal kirurgi for at fjerne løs hud på min mave medmindre min amt gør.

**52**

Vores 19 april 00:17 # 455

Ja, jeg håber jeg kan komme med en beslutning om et første skridt. Tror alle er så modige. Tror, at frygt kan være fordi jeg er bange for ikke at tabe mere end under strømmen, åh så ville føle sig helt mislykket.

**45**

Ons 19 april 21:38 # 456

I dag har jeg været på vægtningen session. Jeg er feberagtig med feber og hoste, så det var en pære at køre 10 miles til hospitalet og derefter sidde der i to timer og lytte. Men det var meget interessant. Jeg tror ikke så meget, som jeg ikke læste online endnu, men nogle spørgsmål blev rettet. Jeg er stadig helt sikker på, at det er det jeg vil have. 

Vi fandt imidlertid ud af, at operationen ikke vil vare indtil årets udgang, og at vi ikke ved, om vi vil blive opereret indtil efter sommeren. Føler godt arbejde. Ønsker jeg havde penge til at gøre operationen privat. Men i morgen vil jeg ringe til de nærliggende hospitaler og se om de har en kortere ventetid.

**53**

Ons 19 april 21:44 # 457

Ja, på XXXX !. Top gode.

**52**

Tors 20 april 00:02 # 458

Men år 45. Det ville betyde at du måtte vente i næsten et år!? Fik du en chance for at fortælle om dine smerter? Eller var den slags det samme for alle .. ved ikke, hvilke rettigheder der er, men jeg håber, at NNN har mulighed for at modtage dig tidligere. Kan du vælge hele landet eller skal du bo i dit amt?

**54**

Tue 4 maj, 13:14 # 459

Hej! 

Sendt i privat præsentation til XXXX Hospital i XXXX samt XXXX på søndag. 

Bliver 20 år gammel og overvægtig siden jeg var 8 år gammel. 

Er 163 cm høj og vejer 120 kg. Hvordan synes du det ligner min del? Har et BMI på 45, og kan tilføje, at jeg har PCOS. 

Og hvornår tror du jeg får svar på henvisningerne? 

// en utålmodig pige

**49**

Fre 5 maj 00:22 # 460

Hej alle! Jeg har endelig fundet ud af, at jeg får min gastric bypass inden sommer. 7/6 er det tid. Begyndte at flyde på mandag og jeg synes, det er rigtig godt. Men jeg vil gå fra 130 kg til 118 kg .. Således 12 kg (!!!) i lidt over fire uger ... Lige nu føles det som det vil aldrig gå selvom fluency er øverst. Enhver, der har formået at tabe så meget på så kort tid? Hvad hvis du ikke kan, selvom jeg ikke snyde? Tror du operationen vil blive udskudt, hvis jeg ikke går ned præcis alle de pounds? Var så meget trist at skulle vente til efteråret.

**55**

Søn 7 maj 18:17 # 461

Hej alle! 
Har fulgt tråden i et stykke tid uden kommentarer, men nu da jeg fangede et stykke af min egen rejse til GBP Jeg vil gerne hoppe ind i tråden :) Bor i XXXX og indsendes min egen kommentar i slutningen af januar. I begyndelsen af april havde jeg tid til medicinske aftaler på bariatrisk klinik, så et informationsmøde i begyndelsen af maj. min  

Henvisning til kirurgen blev sendt omkring en uge siden. Køerne til fedmeoperationer er naturligvis kortere end nogensinde i XXXX . Lægen, som jeg var i begyndelsen af ​​april, sagde at måske jeg kunne have tid til operation før sommeren, men jeg forventer stadig, at det alligevel skal være :)

Hvor længe har det været for en anden at sende en henvisning til kirurgen, indtil du havde besøg og / eller operationstid?

**55**

Søn 7 maj 18:20 # 462

Husk ikke, hvor det var, men jeg ved, at jeg har set eller læst om en pige, der ville drive sig selv, som ikke var gået ned, hvad hun ville gå ned, men det havde ikke været et problem, men hun fungerede som planlagt.

**56**

Man 8 maj 11:21 # 463

Kan du komme ind her? Har lige booket tid på sundhedscentret for en henvisning. Hvad er den mest almindelige grænse for at komme gennem landstinget? Min BMI er 34. 

**27**

Mandag den 8. maj, 16:55 # 464

Hej og velkommen! 

Ifølge 1177 er dette: 
*" BMI tærskel for fedme kirurgi er 40, medmindre du ikke har en underliggende tilstand, såsom diabetes type 2. Sænk derefter BMI grænse 35. Dette fordi de sundhedsmæssige fordele derefter så stora.Vidare bør være flere gange og ved hjælp af flere forskellige metoder, forsøger at opnå permanent vægttab. det er patienten, der afgør, om det er tilfældet. "*jeg ved ikke, om det er anderledes i forskellige amter? Jeg havde lige under 34 og betalt for min egen op.      

**56**

Mandag den 8. maj 18:53 # 465

Tak. 
Jeg har ingen af ​​disse sygdomme, men har type 2-diabetes både hos mor og bedstemor. 

Jeg har ikke deltaget mine tanker med min mand indtil i går. Han var overrasket og havde nogle tanker, vi gik ind for at prøve amtet først og ellers betale for os selv. Bor uden for XXXX, så det bliver enten XXXX eller XXXX . Ville være meget skuffet, hvis jeg ikke får denne hjælp overhovedet, selvom vi betaler. Der er mange penge, men vores hus er steget i værdi, og vi vil stadig låne en renovering, så vi kan tilføje, hvad det koster at drive privat.

**27**

Tirsdag den 9 maj kl. 15:26 # 466

Hej! 

Jeg kørte på XXXX, og jeg kan virkelig anbefale det. Følelse så utroligt omsorgsfuld og sikker. Jeg valgte også rette XXXX for at jeg ønskede at XXXX ville gøre min operation. Han er efter min mening den bedste i Sverige i forhold til GBP. Jeg havde BMI 37, da jeg arbejdede. For seks måneder siden nu og nu har jeg BMI 24. Købte nye jeans idag, str 42 var for stor. Har aldrig købt et par bukser i størrelse 38 i mit liv. Kan ikke tages. Held og lykke!

**56**

Tirsdag 9 maj, 14:51 # 467

Åh, hvad sjovt at læse. Har indset, at jeg ikke vil få nogen operation gennem amtsrådet, fordi jeg ikke er tung nok. Forpasset dem i går aftes og fik et godt svar i morgen. Skal udfylde sundhedscertifikatet og indsende med det samme. De har fritid allerede i juni !!!! Hvor længe har du været syg siden og hvor længe har du følt dig berørt af operationen? Tænker på, om jeg arbejder i slutningen af ​​juni og derefter har en ferie, jeg vil ikke have det til at påvirke familien for negativt. 

**27**

Tue 9 maj, 15:36 # 468

Hej! 

Jeg var syg i tre uger bagefter, og jeg synes det gik meget godt og smidigt. Fandt ikke dårligt bagefter, men det var lidt svært først ved at få nok. Tror ikke det påvirkede familien en masse, jeg fortalte mine børn 5 & 6 Jeg havde opereret mig i maven og måtte spise meget nøje, og de var meget engageret og spurgte, om jeg kunne spise dette eller hint ... ( første uge af flow, derefter blød mad og langsom eskalering). Nu spiser jeg den samme mad som de er, men selvfølgelig meget mindre. I øjeblikket føler jeg mig meget godt, og det er egentlig ikke noget jeg ikke kan spise, men det handler om hvor meget. Er gået ned fra 119,5 kg til 74. Som sagt er det svært at forstå ... 
Det eneste negative jeg ser er, at jeg ofte fryser (i modsætning til før jeg svedte, hvad jeg gjorde), og at jeg tabte meget hår. Men jeg var forberedt på det.

**56**

Ons maj 10 14:22 # 469

Tak for at dele med dig! Heldig jeg har tykt hår.

**56**

Tue 11 maj, 17:09 # 470

Har indsendt et sundhedscertifikat til XXXX og blev kontaktet i dag! Hurtig jerk! Lægen bør se på det og også hente poster fra SU fordi jeg mistede noget blod i forbindelse med fødslen og en anden operation. Håber det stopper ikke! Hvis jeg bliver godkendt, kan jeg betjenes allerede i juni, hvis jeg vil! Bliver næsten sjov, det går hurtigt! Samtidig glad og forventningsfuld. Har brug for at planlægge en del af jobbet, hvis det bliver i juni, men det vil løse!

**26**

Fre 12 maj 02:59 # 471

Spændende! ????

**57**

Fre 12 maj, 16:41 # 472

Hej! 
Har læst hele tråden snart, men har nogle tanker. 
Håber det er okay? 
Du, der har sendt dine egne papirer og blevet afvist på grund af lavt BMI, prøvede du igen senere? Oplever du, at det er nemmere at få et ja med en doktorsafhandling end med et selvværd? Håb for svar! Jeg er 170, vejer 107, BMI 36,6 

**27**

Sø 14 maj 22:31 # 473

Nu er jeg snart i mål, det føles fantastisk! Kun 7,7 kilo tilbage til målet. Næsten 30 kilo er vågen og over en meter i cm rundt om kroppen er forsvundet. Det er rigtig ballt faktisk! 

Nu har jeg lige været træning. Jeg har ikke vovet før, af frygt for at blive fuldstændig diskrediteret i hvor svag og dårlig jeg er. Jeg løb ikke rigtig længe, ​​men jeg løb hele tiden op en rigtig cool tilbage, og jeg var ikke helt færdig, da jeg også var færdig. Løft også nogle vægte. Og ja, jeg er svag, men ikke **så**svag, og jeg **kan**løbe og kroppen føles **god**og sund. Dejligt!

**56**

Mandag den 15. maj kl . 09:50 # 474

Dejligt !!!!

**58**

Mandag den 15. maj kl . 13:54 # 475

Hej! Har læst alle 47 sider i denne tråd, meget spændende! Har sendt en wgentemisd til XXXX og har fået at vide, at den er blevet vurderet og godkendt, og nu venter jeg på en læge! Er så nervøs, men ønsker virkelig dette! Vil du lave en ærme. 

Hvordan laver du alle, der har gjort dette nu bagefter? Har du nogen daglige komplikationer? Forsyreafgivelser eller noget andet?

**27**

Mandag den 15. maj kl . 14:31 # 476

Jeg har det fint, har gjort det hele tiden. Jeg tror, ​​jeg havde det nemt, men det kan ændre sig, det er ikke engang et år siden jeg lavede op.

**58**

Mandag den 15. maj kl . 15:39 # 477

Hvor rart! Håber det fortsætter,  du kan ikke se før og efter billeder her? (Hvis nogen ville have forstået) eller har nogen en inspirerende instagram / blog om den, du kan følge?

**56**

Tue 16. maj, 16:31 # 478

Lige mig informeret om, at lægen godkendte mig til operation! Gå på en konsultation og kan være i drift 8/6 !!!!!! Åh, så hurtigt som dette går!

**58**

Tue 16. maj, 17:15 # 479

Gladhannapanna skrev 2017-05-16 16:31:45 følgende:

Lige mig informeret om, at lægen godkendte mig til operation! Gå på en konsultation og kan være i drift 8/6 !!!!!! Åh, så hurtigt som dette går!

Wow, hvad sjovt !! Hurtigt bud  opdatere os! Hvornår skal du møde en læge?

**56**

Tirsdag 16 maj 18:56 # 480

Mandag!

**56**

Ons maj 17 11:22 # 481

Sidder og forsøger at planlægge min jobbalmanacka efter sygefravær i tre uger. Hvad synes du om muligheden for at arbejde halvtid i den tredje uge? Ellers går min sygefravær lige ind i min ferie, og jeg vil være væk i meget lang tid! Fungerer som XXXX og skulle klare klientmøder, inden de går på ferie. Så har der ikke noget fysisk anstrengende arbejde. Dette føles spændende, skræmmende og utroligt sjovt på én gang! Manden spurgte i går, hvad jeg har tænkt mig at sige til folk, kan du næsten ikke holde på en hemmelighed, men jeg vil nok ikke trompet det ud på Facebook, enten. 

 

**27**

Ons maj 17, 18:30 # 482

Det er sandsynligvis meget anderledes, hvad du kan klare, og det er svært at fortælle. Det anbefales ikke, men hvis det er en enkelt uge, og du skal have en ferie lige efter det, er det okay. Dette er på grund af at lære at tage sig af dig selv, prioritere dit eget helbred og give dig tid til at starte op.

**58**

Sø 21 maj 19:35 # 483

Tæt på noget 56 ! Du vil måske skrive og fortælle dig, hvordan det gik, og hvordan tankerne gik!

**56**

Sø 21 maj 20:11 # 484

Tak! Føler skræmmende, men på samme tid, så fantastisk! Har lige indsendt en ansøgning om at udvide vores realkreditlån. Vil betale for dig selv.

**58**

Mandag den 22. maj, 14:07 # 485

Det er klart, at det er nervøst! Men vil gå så godt. Hvor skal du blive opereret? Jeg har glemt, hvis det står her i forummet et par sider tilbage! Og hvad betaler du? Fortæl mig hvad der skete med lægen! Haha er nysgerrig

**56**

Mandag den 22. maj 18:31 # 486

Nu har jeg mødt min læge, XXXX ngnting. Åh, hvad klogt og beroligende er han, og jeg følte mig ikke mindst dømt eller dårligt som et menneske. Jeg begyndte endda at græde lidt ... Vi besluttede at få en gbp, ikke en ærme. Jeg var klar over, at mine grunde til at gøre dette primært er for mit helbred, at jeg ikke bør lide af diabetes osv. Han sagde, at han forstår hvad jeg mener. 
Start flyder på mandag og tilmelding 31/5 og drift 8/6. 
Fortalte min ældste datter for et stykke tid, brød hun op i alt og mener, at det er skræmmende og endvidere afsluttes operationen i skolen. 
Hvis jeg bringer en USB, vil jeg få en film på min operation. Har nogen fået det? Har du tjekket? 
Han sagde, at det måske virker lidt i sidste uge med sygeorlov.

**56**

Mandag den 22. maj 22:08 # 487

Glemmer at besvare spørgsmålet om prisen, 89000: -.

**26**

Tirsdag 23 maj, 02:14 # 488

XXXX drev mig. Han er helt fantastisk. Men jeg fik ikke nogen film på min rekord. Havde været sej.

**56**

Sø 28 maj 18:52 # 489

I morgen er det tid til at starte strømmen. Jeg føler mig ikke særlig sjovt, men jeg forstår, at det er sidste gang, og det bliver fint. 

Har tilbragt weekend med mandens bror med familie i deres kabine. Hyggelig og meget god mad. Manden fortalte sin bror, at det overhovedet ikke synes at være en god ide. Faktisk er det ligegyldigt, men det føles stadig trist, vel vidende at han ikke vil være den eneste. Mine forældre støtter mig på den anden side til 110%, føler mig dejligt!

**58**

Mandag den 29. maj kl . 18:33 # 490

Jeg er også bange for at få så nedlatende kommentarer. Det er for mig, men det er aldrig sjovt, når folk vil have meget at sige, hvis noget er negativt, er det godt at dukker op! Men jeg glæder mig til dig og vil gerne følge din tur her!

**56**

Mandag 29 maj 19:59 # 491

Tak! 
Første dag på pulver. Rid Modifasts havregryngrød til morgenmad, ok ok faktisk. 
Har vejet og mødt mig, var grådig følelse at se hvor stort jeg er, men tror det vil føles godt at se, hvor meget jeg er faldet! 
Målt hals, overarm, bryst, talje, bryst, lår, knæ og hvad. Jeg målte mine arme og ben på venstre side. 
Ser frem til at føle bedre tøj snart, har nogle favoriser, der er lidt små nu, jeg vil snart kunne få dem uden at frygte, at knappen er i bevægelse!

**56**

Ons 31 maj 22:06 # 492

Tilmelding i dag, tredje dag på pulver. Nu kommer det tættere på stormen!

**58**

Tue 1 Jun 07:12 # 493

Åh, hvad spændende! Held og lykke,  har du problemer med at powdering eller går det godt? En uge tilbage kun!

**56**

Tue 1 Jun 09:40 # 494

Faktisk er jeg sikker på, jeg er sulten hele tiden, men det er op til dig. Efter at jeg er gået i pulver før, er det omkring 500 kalorier om dagen fordelt på tre gange. Nu skal jeg "spise" fire gange om dagen og omkring 800 kalorier. Meget lettere! Talte med diætist i går og spurgte, om jeg kunne tygge på nogle knækbrød eller måske en halv gulerod, når jeg bliver for sulten, og det er OK, så længe jeg ikke spiser mere end 1.000 kalorier. Må sige, at pulveret bliver meget sjovere siden jeg sidst gjorde det, modifasts spiser havregryn til morgenmad hver dag, og det virker fint, ellers kører jeg på deres chokolade drink og drejebænke med naturdiets chokolade sorter, de har flere gode! 


Jeg spurgte om, hvad der er rimeligt vægttab af pulveret og i fremtiden, sygeplejersken tror jeg vil tabe omkring 4 kg pulver, og så er det almindeligt at tabe 1-1,5 kg / uge. Jeg har ingen håb om at gå ned mere end måske 10 kg om sommeren, vil gerne holde mine forventninger lave. Samlet set vil jeg gå ned mindst 25, helst 30 kg. Min største bekymring er, at jeg ikke vil gå ned .....

**58**

Tue 1 Jun 13:51 # 495

Godt! Nu er det sidste gang du har pulver også, hvilket også gør det lidt lettere at skille sig ud! 

Selvfølgelig vil du tabe dig, men det er tanker som mig også. Tænk hvis du gør det for skyldigt, men det føles urimeligt! Selvfølgelig vil du tabe  , men godt at holde lave forventninger til mål, så vil du ikke være så skuffet, hvis det ikke går som du har planlagt, kan man ende op på et plateau i flere måneder, selv før det er slukket igen, så tåladmod er stadig a og åh, selvom jeg ikke har noget haha ​​..

**56**

Mandag 5 Jun 07:59 # 496

En uge med strøm og minus 2,5 kg. Har snydt lidt, så det kunne have været mere. Derudover vil jeg have til enhver tid og så tænker jeg altid på en eller to kg. Ikke mange dage tilbage nu. Spændende, irriterende og skræmmende på samme tid. 
Både mine søstre og forældre støtter mig til 110 procent, føler mig godt. Sørg også for! Hans forældre er neutrale, og hans bror var lidt negativ sidste gang vi mødtes. Mine kolleger, der ved, om det er entusiastisk, det er ferie tid, så jeg håber at have ændret lidt indtil august, når alle er på plads igen.

**58**

I går 20:10 # 497

Held og lykke i morgen Hvordan føles det ?? Vil du gerne have en slags kirurgisk historie, så hvis du kan føle dig  så nysgerrig og længes efter min egen operation!

**56**

I går 21:39 # 498

Jotack, det føles godt. Men selvfølgelig begynder jeg at blive lidt nervøs! Har bare showered descutanen og ændret lagnen. Pak tasken også, kun telefon, iPad og oplader venstre til i morgen. Tag straks den sidste ryste inden operationen, i morgen bliver det bare juice! Vil veje mig i morgen med tydeligvis. Skrive igen bagefter!

+

**Første forskning**
